# Covalent Ag-holey MXene link at interface boosting electrochemical dechlorination

**DOI:** 10.1038/s41467-026-75560-1

**Published:** 2026-07-14

**Authors:** Hao Zhang, Chenxu Liu, Hao Wang, Xin Ma, Ruochen Wang, Jiansheng Li, Jiayin Yuan, Miao Zhang

**Affiliations:** 1 School of Chemistry, Xi’an Jiaotong University, Xi’an, PR China; 2 Key Laboratory of Jiangsu Province for Chemical Pollution Control and Resources Reuse, School of Environmental and Biological Engineering, Nanjing University of Science and Technology, Nanjing, PR China; 3 Department of Chemistry, Stockholm University, Stockholm, Sweden

**Keywords:** Two-dimensional materials, Pollution remediation, Porous materials

## Abstract

Incorporating Ag species into conductive matrices can mitigate volumetric expansion and stabilize electrode structures in capacitive deionization (CDI). Yet, this approach may suffer from severe charge transfer resistance at their heterogeneous interface featuring physical contact or weak intermolecular link. To tackle this challenge, we linked Ag single atoms and nanoparticles covalently to holey MXene (HMX) via an in situ etching-and-reduction strategy, in which atomically dispersed Ag in covalent bond with HMX is generated and serves as pivots to guide growth of Ag nanoparticles. The elaborate covalent link promotes Ag-to-HMX electron transfer and creates electron-deficiency on Ag that facilitates faradaic dechlorination. Meanwhile, the perforated HMX nanosheets with rich in-plane nanopores assemble into thin films possessing a spatially interconnected porous matrix, enabling fast cross-film transfer of both electrons and ions. Consequently, the as-made hybrid electrodes exhibit enhanced dechlorination capacity (156.63 ± 2.6 mg g^−1^), charge efficiency (91.2 ± 4.5%), and cyclic stability (>90% retention in 50 cycles) compared to the control samples. This synthetic strategy integrates Ag-mediated covalent interfacial bridging with pore-engineered MXene, providing a paradigm for designing CDI dechlorination electrodes.

## Abstract (web summary)

This study develops an in situ etching-and-reduction strategy to covalently anchor Ag nanoparticles onto conductive holey MXene matrix using Ag single atoms as growth pivots, enabling synergetic ion intercalation and faradaic dechlorination.

## Introduction

Chlorine pollution arising from industrial effluents has been an increasingly pressing environmental issue^1^. Chloride anions (Cl^−^) at sufficient concentration can cause soil salinization in natural environments and corrode stainless steel in industrial facilities, thereby threatening ecological health and production safety^2,3^. These concerns have sparked a persistent pursuit of clean and efficient technologies for capturing and/or storing excessive Cl^−^ in water, which is also critical in desalination of both seawater and brackish water, as Cl^−^ accounts for a significant portion of the total salinity^4^. Capacitive deionization (CDI) has emerged as a promising electrochemical technology for ion removal, owing to its high operational efficiency, low energy consumption, easy regeneration, and environmental friendliness^5–7^. The CDI process leverages a low-voltage electric field (generally < 2.0 V) to drive ion migration and storage in oppositely charged electrodes, highlighting the critical role of electrode materials^8,9^. Although conventional carbon-based materials are widely used in CDI, they inherently rely on the electrical double layer (EDL) mechanism and suffer from issues like co-ion repulsion effect and adverse side reactions, leading to a limited adsorption capacity^10–12^. Advances in the field of electrochemical energy storage over the past decade, particularly in the area of alkali metal ion (Li^+^, Na^+^, and K^+^) batteries, have spurred significant interest in faradaic materials as promising alternatives to conventional CDI electrodes. So far, most studies have focused on cathode materials for cations capture (e.g., transition metal oxides^13,14^, transition metal dichalcogenides^15,16^, and Prussian blue analogs^17,18^), leaving behind the matching faradaic anode materials for dechlorination scarcely reported.

The Ag/AgCl electrode, employing a redox conversion reaction of Ag + Cl^−^ ⇌ AgCl + e^–^, holds considerable promise for CDI dechlorination owing to its high theoretical capacity of 248 mAh g^−1^, superior Cl^−^ selectivity, and good electrochemical reversibility^19,20^. Nevertheless, the drastic volumetric expansion (ca. 250%) of Ag-based materials during repeated Cl^−^ capture and release processes inevitably results in kinetics decay, cyclic instability, and ultimately mechanical pulverization of the Ag-based electrode^21–23^. Furthermore, the poor electrical conductivity of AgCl resists charge transfer and impedes its reconversion to Ag nanoparticles (NPs)^24,25^. To tackle these challenges, reducing the particulate size and integrating the Ag/AgCl into conductive substrates have been widely adopted^26^. For instance, porous carbon materials, such as activated carbon and graphene, have been tailored to host Ag NPs to buffer volumetric change and facilitate ion migration^27,28^. Nevertheless, the Ag NPs on these carbon substrates are typically insufficiently confined and retain certain freedom of mobility, resulting in structural destabilization during prolonged electrochemical cyclic tests. Recently, Wei et al.^29^ prepared an Ag-based metal-organic framework (MOF) electrode via solvothermal treatment to coordinate Ag nodes with organic ligands. While this electrode achieved molecular dispersion of Ag and enhanced interfacial stability, the MOF’s intrinsically low conductivity hindered charge transfer. Collectively, these limitations call for new architectures in Ag-based electrodes for faradaic dechlorination that concurrently accommodate volumetric change and minimize interfacial charge-transfer losses.

Two-dimensional (2D) transition-metal carbides/nitrides, known as MXenes, have emerged as promising supports for confined nanoarchitectonics of Ag NPs due to their hydrophilic surfaces, ultrathin morphology, and high electrical conductivity^30,31^. The monodisperse MXene nanosheets can assemble into an interconnected conductive network to accommodate and immobilize Ag NPs, and their mechanical strength and flexibility enable freestanding film electrodes without conductive additives or polymeric binders. Despite these merits, simple physical contact between Ag NPs and MXene is insufficient to address the fundamental limitation of high charge-transfer resistance at the heterogeneous interface, thereby resulting in sluggish reaction kinetics^32^. Meanwhile, the tortuous diffusion pathways through the interlayer nanoconfined channels together with limited ion-accessible slit-like gaps along the edges of MXene nanosheets impede the rapid ion penetration from the surface to the interior of the electrode^33–35^. Therefore, the simultaneous realization of strong Ag NPs−MXene interfacial coupling, efficient ion-transport pathways, and robust structural and chemical stability is essential for high-performance CDI dechlorination.

Herein, we leverage Ag single atoms (SAs), introduced via an in situ etching-and-reduction strategy, as atomic-scale growth pivots to tackle the interfacial connectivity issue between Ag NPs and Ti_3_C_2_T_*x*_ MXene. Triggered by a spontaneous redox reaction between Ag^+^ cations and the unsaturated Ti sites in MXene, Ag atoms first deposit onto the MXene surface in a monoatomic state and subsequently evolve into a nanoparticulate state, accompanied by the simultaneous generation of Ti vacancies and in-plane holes on the MXene nanosheets. By precisely regulating the metal growth kinetics, the formed Ag NPs, together with Ag SAs, can firmly anchor to holey MXene (HMX) through interfacial covalent linkage, thereby accelerating charge redistribution and electron transport at the heterogeneous interface. In this case, HMX can fully serve as a conductive support and flexible volume-buffer layer for Ag NPs, thereby facilitating the optimal utilization of redox sites and alleviating volumetric changes. Concurrently, the nanopores engineered into individual HMX nanosheets were interconnected upon stacking of HMX into vertical transport highways for ion migration, in addition to the common horizontal interlayer transport channels. As a result, the as-optimized film electrode delivers improved dechlorination performance for simulated brackish water and circulating cooling water. This study provides a paradigm for the precise design of Ag/MXene hybrid films with a defined interfacial chemistry and enhanced ion-transport kinetics.

## Results

### Material synthesis and characterization

**Fig. 1:**
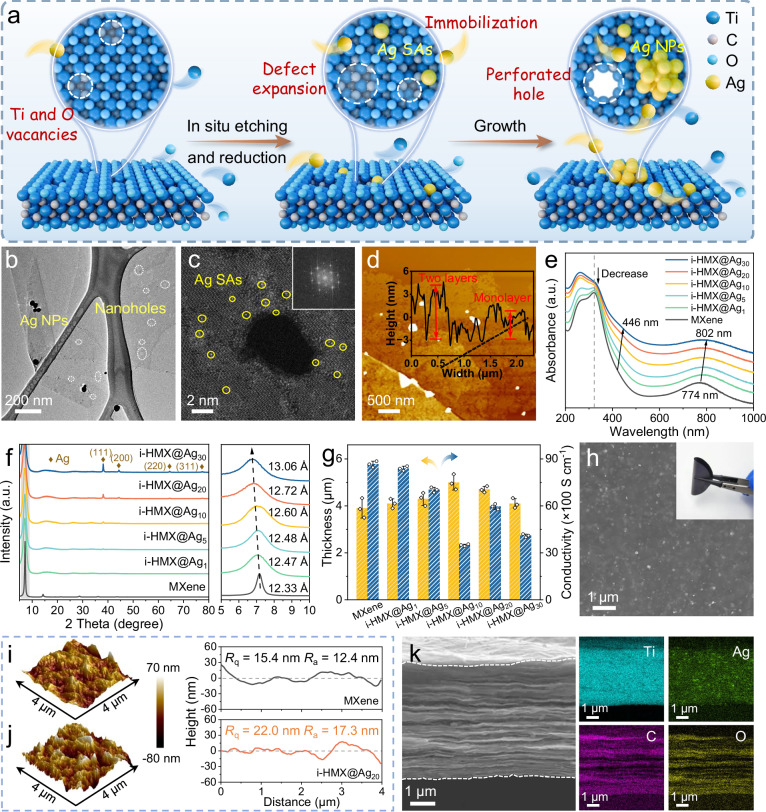
Synthesis and structural evolution from pristine MXene to i-HMX@Ag_*x*_. **a** Schematic illustration of the i-HMX@Ag_*x*_ formation by in situ etching-and-reduction method. Ag single atoms (SAs) are immobilized and grow into nanoparticles (NPs) accompanied by the formation of nanoholes. **b** Transmission electron microscopy (TEM) image, (**c**) high-angle annular dark-field scanning TEM (HAADF-STEM) image (inset is the corresponding fast Fourier transform pattern), and (**d**) atomic force microscopy (AFM) image of i-HMX@Ag_20_. The white circles in (**b**) represent nanoholes and the yellow circles in (**c**) represent Ag SAs. **e** Ultraviolet-visible (UV-Vis) spectra of MXene and i-HMX@Ag_*x*_ dispersions. **f** X-ray diffraction (XRD) patterns, (**g**) thickness (left y-axis) and conductivity (right y-axis) of MXene and i-HMX@Ag_*x*_ films. **h** Surface scanning electron microscopy (SEM) image of i-HMX@Ag_20_ film. The inset shows a digital photograph of the freestanding film, demonstrating its flexibility. AFM images and surface roughness of (**i**) pristine MXene film and (**j**) i-HMX@Ag_20_ film. **k** Cross-sectional SEM image and corresponding energy-dispersive spectroscopy (EDS) elemental mapping of i-HMX@Ag_20_ film. Data in (**g**) are presented as mean ± standard deviation; error bars represent standard deviation; *n* = 3 independent films.

Ti_3_C_2_T_*x*_ MXene, synthesized by the liquid exfoliation method^36,37^, was employed as a reductive support for in situ reduction and immobilization of Ag SAs and NPs. As illustrated in Fig. 1a, the low-valence Ti species (Ti^2+^/Ti^3+^) on the MXene surface can undergo spontaneous redox reactions with Ag^+^ and deposit Ag atoms alongside the generation of Ti and O vacancies. These generated vacancies, particularly the Ti vacancies, serve as anchoring sites that enable covalent bonding between the Ag species and the surrounding C/O atoms of the MXene lattice. As the reduction reaction progressed to accumulate Ag atoms, the initially immobilized Ag SAs on the MXene surface gradually grew into Ag NPs, simultaneously with the evolution of defects on MXene into in-plane nanoholes, yielding HMX. These resulting Ag SAs and NPs were uniformly anchored on both sides of the HMX nanosheets via covalent interactions, forming hybrid materials termed i-HMX@Ag_*x*_ hereafter. Of note, in order to visually represent the design of the gradient experiment, the subscript “*x*” in i-HMX@Ag_*x*_ indicates the mass percentage of the added AgNO_3_ in relation to MXene, rather than the actual Ag content. Distinct from conventional Ag/MXene composites that often rely on multilayered or non-porous MXene and contain appreciable AgCl residues^30,31^, our approach yields an HMX framework from monolayer nanosheets, creates covalent Ag−C/O interfacial bonds, and leaves no detectable AgCl residue, thereby ensuring enhanced dechlorination performance.

The morphological evolution of MXene nanosheets from the unpunched to the holey state is tracked along this process. After completely etching the Al atom layers away from bulk Ti_3_AlC_2_ MAX phase (Supplementary Fig. 1) and the subsequent delamination, the as-prepared MXene nanosheets exhibit a flat and smooth surface (in a unpunched state) with a uniform thickness of ca. 2 nm (Supplementary Fig. 2). Of note, the oxidation of low‑valence Ti species during the in situ etching-and-reduction process inevitably produces TiO_2_‑type by‑products, as demonstrated by the spindle‑like TiO_2_ in transmission electron microscopy (TEM) image and a characteristic band at approximately 150 cm^−1^ in Raman spectrum (Supplementary Fig. 3). Therefore, L(+)-tartaric acid was employed as a mild scavenger to eliminate these impurities and ensure the purity of the i-HMX@Ag_*x*_. In contrast, the TEM image of i-HMX@Ag_20_ (Fig. 1b) shows numerous in-plane nanoholes with Ag NPs firmly attached to the clean surface of HMX after the acid washing process. The use of a higher dosage of the Ag^+^ in this process increased the density and size of both Ag NPs and in-plane holes in the HMX nanosheets (Supplementary Fig. 4); however, excessive Ag^+^ dosage results in cracked flakes and compromised structural integrity of the HMX support. The high-angle annular dark-field scanning TEM (HAADF-STEM) image in Fig. 1c displays a number of bright spots corresponding to atomically dispersed Ag SA sites (marked with yellow circles), along with nanoscale defects and mesoporous voids across the entire HMX basal plane. It is worth noting that this mild in situ etching process did not disrupt the inherent crystallinity of MXene, as demonstrated by the corresponding intact fast Fourier transform (FFT) pattern (inset of Fig. 1c). The energy-dispersive spectroscopy (EDS) mapping further confirms the homogeneous distribution of Ag species on HMX (Supplementary Fig. 5). The atomic force microscopy (AFM) image (Fig. 1d) visualizes the hole-rich HMX nanosheets with a larger thickness (ca. 3 nm) than that of pristine MXene (ca. 2 nm), which can be attributed to the surface-decorated Ag species. The coexistence of Ag SAs and NPs on HMX arises from a kinetically controlled two-stage growth process. Ag SAs are expected to be first immobilized by strong coordination with surface atomic vacancies on MXene, thereby establishing covalent interactions with MXene and serving as anchoring sites. These Ag SAs subsequently act as nucleation centers for the growth of Ag NPs under continuous reduction of Ag^+^ and thus a steady supply of Ag SAs, while the spatial confinement and the limited reactive sites prevent aggregation of Ag NPs. This continuous, dynamic growth path yields a stable hybrid architecture in which both Ag SAs and NPs coexist synergistically.

The i-HMX@Ag_*x*_ samples are colloidally dispersible in water, and their lateral size distributions at the micrometer scale are comparable to that of the pristine MXene (Supplementary Fig. 6). In the ultraviolet-visible (UV-Vis) absorption spectra (Fig. 1e), the progressive attenuation of the absorption peak at 322 nm, characteristic of MXene’s interband transitions, indicates a gradual modulation of the MXene electronic structure driven by charge redistribution upon interfacial covalent bonding. Concurrently, a gradually noticeable bulge peak appears at 446 nm, signaling the onset of Ag NPs formation. The mean size of the Ag NPs in HMX@Ag_1_ was measured to be approximately 20 nm, which is sufficient to induce localized surface plasmon resonance^38^. Upon continuous Ag^+^ dosage, the peak at 774 nm undergoes a gradual red-shift to 802 nm, which provides direct spectroscopic evidence for the progressive growth of Ag NPs. To underscore the essence of the in situ etching-and-reduction method, a control sample (denoted as HMX/Ag) was prepared by physically blending the pre-synthesized HMX and commercial Ag NPs, maintaining the same Ag mass ratio as that in i-HMX@Ag_20_. The HMX framework was produced ex situ via a H_2_O_2_-assisted etching treatment^33^. It should be noted that both Ag^+^ and H_2_O_2_ create in‑plane holes in MXene through the selective oxidative etching of Ti species, accompanied by the removal of surface terminal groups, but their reaction kinetics differ. By fine‑tuning the etching conditions of H_2_O_2_, the produced HMX exhibits in-plane porosity and thickness comparable to that of i-HMX@Ag_20_ (Supplementary Fig. 7). After mixing HMX with commercial Ag NPs, the obtained HMX/Ag shows comparable dispersibility and lateral size to i-HMX@Ag_20_ (Supplementary Fig. 8). This structural similarity between i-HMX@Ag_20_ and HMX/Ag ensures a fair comparison, thereby allowing us to attribute the difference in performance to the interfacial chemistry, rather than morphological discrepancy. Intriguingly, both HMX and HMX/Ag retained the characteristic UV-Vis absorbance peaks at 322 and 774 nm, with no noticeable peak shift (Supplementary Fig. 9a). This observation starkly contrasts with the spectral shift observed in i-HMX@Ag_*x*_, revealing that the electronic structure of HMX was modulated not by the simple physical attachment of Ag NPs to HMX, but specifically by the interfacial interactions and charge transfer enabled by the in situ-formed Ag species.

All prepared samples exhibit highly negative Zeta potentials (more negative than −40.0 mV) in a neutral medium (Supplementary Fig. 10) due to the abundant −OH and −O functional groups on MXene. Notably, the Zeta potentials of i-HMX@Ag_*x*_ and HMX/Ag are slightly lower than that of pure MXene (−42.2 ± 2.0 mV) due to the etching of the unsaturated Ti atoms at the edges^33^. As the etching intensity increases, this value gradually decreases from −43.6 ± 0.9 mV of i-HMX@Ag_1_ to −48.5 ± 3.3 mV of i-HMX@Ag_30_. Overall, the high absolute zeta potential values of all dispersions provide strong electrostatic and colloidal stabilization against aggregation, thus ensuring the successful fabrication of uniform, crack-free films. These dispersions were then assembled into a series of flexible, freestanding round films (4 cm in diameter) via vacuum filtration (Supplementary Fig. 11). An inductively coupled plasma-optical emission spectrometer (ICP-OES) was employed to quantify the actual Ag loading after digesting the i-HMX@Ag_*x*_ films with nitric acid. As displayed in Supplementary Table 1, the Ag content gradually increases from 0.22 wt.% in i-HMX@Ag_1_ to 13.32 wt.% in i-HMX@Ag_30_ but all remain below the theoretical value, which may be ascribed to the incomplete reaction between Ag^+^ and MXene and partial nanoparticle detachment during the washing process. X-ray diffraction (XRD) analysis was conducted to characterize their phase structures and interlayer spacings. As shown in Fig. 1f, with increasing Ag^+^ dosage, the (002) diffraction peak of i-HMX@Ag_*x*_ progressively shifts to lower angles (from 7.2° to 6.8°), indicating an increase in the interlayer spacing from 12.33 to 13.06 Å as derived from the Bragg equation (2*d*sin*θ* = n*λ*)^39^. Simultaneously, the i-HMX@Ag_*x*_ exhibits distinct diffraction peaks at 2*θ* = 38.1°, 44.3°, 64.4°, and 77.4°, indexed to the (111), (200), (220), and (311) crystal planes of metallic Ag (JCPDS #04-0783), respectively^40,41^. Both the intensity of the Ag diffraction peaks and the interlayer spacing scale progressively with the Ag⁺ dosage. To further explore the growth process of Ag NPs, a transition sample with a very low Ag dosage was prepared by adding AgNO_3_ and MXene at a mass percentage of 0.1% (termed i-HMX@Ag_0.1_). As exhibited in Supplementary Fig. 12a, the HAADF-STEM image of i-HMX@Ag_0.1_ reveals several scattered bright spots, ascribing to the immobilized atomically dispersed Ag SAs through in situ reduction. This reaction process also led to nanoscale defects on the MXene surface. It is worth noting that no characteristic peaks related to metallic Ag were observed in the XRD spectrum of i-HMX@Ag_0.1_ (Supplementary Fig. 12b), suggesting that these small amounts of Ag could not form NPs. These results further indicate that a small amount of Ag^+^ is preferentially reduced and fixed as monatomic form on the surface of MXene, and then gradually grows into NPs as the concentration of added Ag^+^ increases. In contrast, the interlayer spacing of HMX decreases to 11.62 Å (Supplementary Fig. 9b), likely due to enhanced interlayer interactions induced by the loss of −OH groups during H_2_O_2_ etching^42^. Although the physical incorporation of Ag NPs in the HMX/Ag expanded the interlayer spacing to 12.37 Å, this value remained substantially lower than that of the i-HMX@Ag_*x*_, resulting in inferior ion transport kinetics. The i-HMX@Ag_*x*_ film exhibits a volcano-type evolution in thickness, with an initial increase followed by a decrease (Fig. 1g). This trend can be attributed to the initial gallery expansion induced by the incorporation of Ag NPs, followed by partial nanosheet fragmentation caused by excessive Ag^+^-driven etching. Similarly, the electrical conductivity first decreases, consistent with MXene etching, and then rebounds markedly in the i-HMX@Ag_20_ film. This conductivity recovery is presumably attributed to the synergistic contribution of etched HMX flakes and abundant Ag NPs, which collectively established a highly interconnected, conductive network for electron transport.

Scanning electron microscopy (SEM) was carried out to investigate the morphology and microstructure of the films. As shown in Fig. 1h and Supplementary Fig. 11, the pristine MXene film has a smooth, wrinkle-free surface, while samples with increasing Ag loading (from i-HMX@Ag_1_ to i-HMX@Ag_30_) show a progressive increase in both the density and size of Ag NPs. This evolution in surface topography is further corroborated by AFM measurements (Fig. 1i, j, and Supplementary Fig. 13), which reveal a continuous growth in surface roughness. Moreover, the intrinsic toughness and flexibility of HMX nanosheets render the i-HMX@Ag_20_ film freestanding and flexible (inset of Fig. 1h). The variation in roughness is also closely related to the surface hydrophilicity of the films. As depicted in Supplementary Fig. 14, i-HMX@Ag_1_ film exhibits a larger water contact angle (WCA) of 70.8° than pristine MXene (41.2°), which can be attributed to the etching-induced elimination of some hydrophilic groups (e.g., −OH) on the HMX surface. Interestingly, the further rise in surface roughness decreased the WCA to 30.2°, which is consistent with the Wenzel model given the intrinsic hydrophilicity of both MXene and metallic Ag^43^. In contrast, the HMX/Ag film shows higher surface roughness yet lower hydrophilicity, both of which are attributable to the severe agglomeration of Ag NPs due to simple physical blending. This agglomeration phenomenon creates disordered surface sites and consequently disrupts the wetting uniformity achievable by the in situ etching-and-reduction method. The cross-sectional SEM images demonstrate a well-aligned, compact, and layered architecture across all films (Fig. 1k and Supplementary Fig. 15). The elemental mappings reveal that the Ag species of i-HMX@Ag_*x*_ are uniformly interspersed throughout the MXene galleries, whereas coarse, agglomerated particles dominate in HMX/Ag. The enlarged cross-sectional images further corroborate this structural contrast (Supplementary Fig. 16 and Supplementary Fig. 17). The homogeneously distributed Ag NPs can function as intercalated pillars to prevent HMX nanosheets from self-stacking and increase local curvature, thereby facilitating Cl^−^ accessibility and charge transfer.

The Raman spectra of these films exhibit representative vibrational bands ascribed to MXene, including *A*_1g_ (out-of-plane) vibrations at 198 and 717 cm^−1^ and *E*_g_ (in-plane) vibrations at 288, 390, and 620 cm^−1^ (Supplementary Fig. 18a)^44^. Compared to pristine MXene, the intensity of these bands is slightly reduced following in situ etching. Moreover, the *G*-band at 1585 cm^−1^ shows a pronounced enhancement in i-HMX@Ag_*x*_, suggesting the presence of residual carbon near the in-plane holes^33^. The Fourier-transform infrared spectroscopy (FT-IR) was employed to trace changes in surface terminal groups. As shown in Supplementary Fig. 18b, the band assigned to −OH ( ~ 3500 cm^−1^) gradually diminishes during the in situ etching process, accompanied by the emergence of C−H signals (2850 and 2915 cm^−1^), demonstrating the detachment of oxygenated groups as the corrosion of Ti atoms^45^. These phenomena are consistent with those observed during the ex situ H_2_O_2_-assisted etching process (Supplementary Fig. 9c, d). Collectively, these spectroscopic results confirm that our in situ etching-and-reduction approach is mild and reliable, successfully introducing in-plane holes and Ag species without compromising the structural integrity of the MXene framework. The generality of this strategy was further explored by replacing Ag^+^ with Pd^2+^ under identical conditions. As shown in Supplementary Fig. 19a, the TEM image of the prepared i-HMX@Pd reveals that numerous Pd NPs are attached to the HMX. The high‑resolution TEM (HRTEM) clearly shows lattice fringes corresponding to metallic Pd (Supplementary Fig. 19b). Moreover, the XRD pattern of i-HMX@Pd (Supplementary Fig. 19c) exhibits distinct diffraction peaks of Pd along with the (002) peak of MXene, which is similar to the i-HMX@Ag_*x*_ samples. The HAADF-STEM and corresponding EDS elemental mapping (Supplementary Fig. 19d–i) further demonstrate the uniform distribution of Pd across the entire nanosheet. Collectively, these results demonstrate that this in situ etching-and-reduction strategy can be extended to other noble metals, such as Pd, that can undergo spontaneous redox reactions with the low-valence Ti species on MXene, which offers a general platform for constructing covalently linked metal/MXene heterointerfaces for diverse electrochemical applications.

**Fig. 2:**
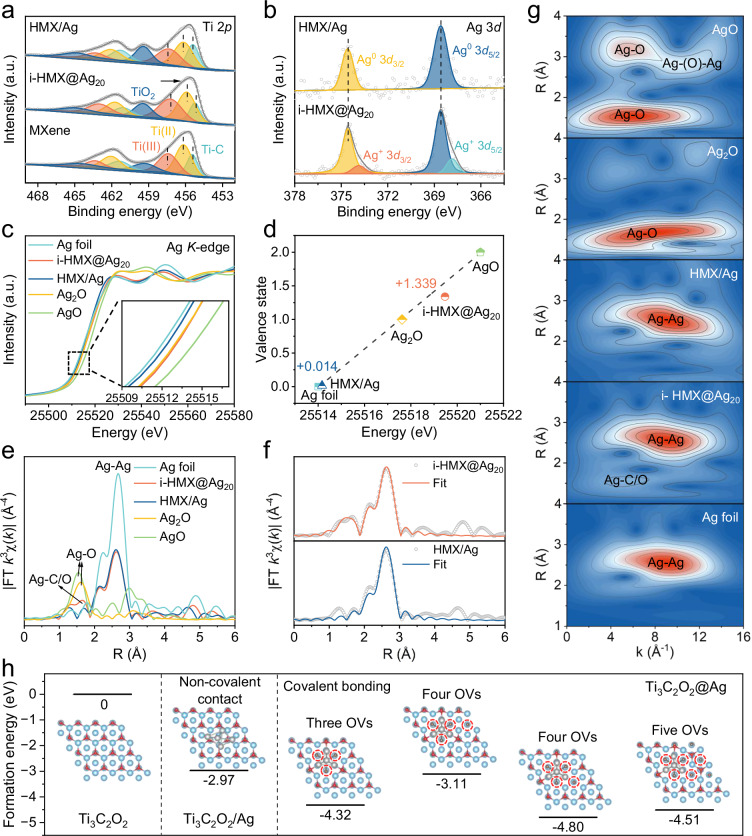
The chemical state and electronic structure characterizations. **a** Ti 2*p* X-ray photoelectron spectroscopy (XPS) spectra of MXene, HMX/Ag, and i-HMX@Ag_20_. **b** Ag 3*d* XPS spectra of HMX/Ag and i-HMX@Ag_20_. **c** X-ray absorption near edge structure (XANES) spectra at the Ag K-edge (inset is the partial enlarged XANES plots from 25509 to 25517 eV), **d** the corresponding oxidation state (linear fit, R^2^ = 0.9992), and **e** Fourier-transform extended X-ray absorption fine structure (EXAFS) spectra of Ag foil, Ag_2_O, AgO, HMX/Ag, and i-HMX@Ag_20_. **f** EXAFS fitting results in R space of HMX/Ag and i-HMX@Ag_20_. **g** Wavelet transform (WT) of Ag K-edge EXAFS spectra of Ag foil, Ag_2_O, AgO, HMX/Ag, and i-HMX@Ag_20_. **h** Formation energy diagram of different metal-support contact models with different surface oxygen vacancies (OVs).

The chemical state and local coordination environment of the samples were probed by X-ray photoelectron spectroscopy (XPS) and X-ray absorption fine structure (XAFS) analyses. The XPS survey spectra show the main peaks of Ti 2*p*, C 1 *s*, O 1 *s*, and Ag 3 *d* (Supplementary Fig. 20). The high-resolution Ti 2*p* XPS spectra in Fig. 2a reveal that the i-HMX@Ag_20_ features a negatively shifted binding energy (455.2, 455.9, and 457.2 eV for Ti−C 2*p*_3/2_, Ti (II) 2*p*_3/2_, and Ti (III) 2*p*_3/2_, respectively) in comparison to that of the pristine MXene (455.5, 456.2, and 457.5 eV)^46^. However, no peak shift was observed for HMX/Ag, revealing that no interfacial electron transfer occurred between Ag and HMX. Therefore, the electron density around Ti atoms does not increase, and the Ti 2*p* binding energy remains unchanged in physically mixed HMX/Ag. This result demonstrates that the in situ etching-and-reduction process induced the electronic redistribution at the heterogeneous interface. Furthermore, the high-resolution Ag 3 *d* XPS spectrum of i-HMX@Ag_20_ exhibits two emerging peaks at 367.8 and 373.8 eV compared to HMX/Ag (Fig. 2b), corresponding to the Ag^+^ 3*d*_5/2_ and Ag^+^ 3*d*_3/2_^29^, indicative of the coexistence of different valence states of Ag species. These results indicate that electrons are transferred between Ag NPs and the HMX support, likely through the covalently linked Ag−HMX interface, resulting in an interfacial built-in electric field and an active Ag surface. As expected, excessive Ag^+^ dosage will cause overgrowth of Ag NPs and thus weak covalent connection to HMX, shifting the peak position back toward its initial value of binding energy (Supplementary Fig. 21). Similar spectral evolution can be observed in the high-resolution C 1*s* and O 1*s* XPS spectra (Supplementary Fig. 22). To further explore the charge separation behaviors between Ag and HMX, Kelvin probe force microscopy (KPFM) was performed on i-HMX@Ag_20_ and HMX/Ag using silicon wafers as the conductive substrate. As shown in Supplementary Fig. 23, the surface potential difference between HMX and Ag in i-HMX@Ag_20_ is ca. 3.2 μV, which is markedly smaller than that of HMX/Ag (ca. 7.4 μV). The larger potential difference in the physically mixed HMX/Ag sample indicates hindered electron transfer due to the large interfacial resistance at the point contacts. In contrast, the covalent Ag−HMX interface in i-HMX@Ag_20_ facilitates efficient electron flow, thereby reducing the potential difference, consistent with the XPS analyses. Collectively, these findings underscore the critical role of the covalent crosslinks between Ag species and HMX, which not only create an electron-deficient Ag surface but also establish a highly efficient charge transport interface for Cl^−^ capture.

The Ag K-edge X-ray absorption near-edge structure (XANES) spectrum of i-HMX@Ag_20_ exhibits a significant positive shift relative to that of HMX/Ag (Fig. 2c), indicating a higher oxidation state of Ag in i-HMX@Ag_20_ and thus stronger interfacial electron transfer between Ag and HMX. The calculated average oxidation state of the Ag element is +1.3 and close to 0 in i-HMX@Ag_20_ and HMX/Ag (Fig. 2d), respectively, which is consistent with the XPS results of Ag 3*d*. The extended X-ray absorption fine structure (EXAFS) further provides insight into the local coordination environment of Ag sites (Fig. 2e and Supplementary Fig. 24a). For both i-HMX@Ag_20_ and HMX/Ag, a scattering peak at ≈ 2.62 Å is observed, which corresponded to the Ag−Ag bond (Fig. 2e). Notably, the distinct scattering peak at ≈ 1.66 Å in i-HMX@Ag_20_ is slightly longer than the Ag−O bond in Ag_2_O (≈ 1.62 Å), suggesting the formation of Ag−C/O coordination^47^. This elongation is ascribed to the local lattice distortion induced by the generated heterogeneous interface, indicating that the Ag on the HMX substrate was not only bonded to surface oxygen functionalities but also directly to the HMX lattice. The fitting results of EXAFS (Fig. 2f, Supplementary Fig. 24b, c, Supplementary Fig. 25, and Supplementary Table 2) support the coexistence of both Ag SAs and Ag NPs in the i-HMX@Ag_20_, with the SAs occupying Ti vacancies and bonding to C atoms, and the NPs bonding to surface O atoms. The wavelet transform (WT) of Ag K-edge EXAFS was further adopted to elucidate the coordination environment of Ag in a spatial resolution. As shown in Fig. 2g, the intensity maxima observed in the i-HMX@Ag_20_ can be attributed to Ag−C/O and Ag−Ag coordination, validating that Ag SAs and Ag NPs are anchored at HMX in a synergetic interfacial coordination mode. Taken together, the synchronous covalent bonding of Ag to the HMX lattice and surface groups in i-HMX@Ag_20_ enables efficient charge redistribution, consistent with the XPS analysis.

Density functional theory (DFT) calculations were performed to further elucidate the metal-support interactions. Taking Ti_3_C_2_O_2_ (Supplementary Fig. 26a) as a representative MXene model, a Ag cluster was directly placed on its surface to simulate the HMX/Ag control with weak interfacial contact (Supplementary Fig. 26b), due to the absence of a Ag−O signal for HMX/Ag from experimental observations (Fig. 2e). In contrast to the HMX/Ag model, we constructed covalent crosslinking models by substituting a Ti atom with a Ag atom in the Ti_3_C_2_O_2_ lattice, a process that inevitably introduces varying distributions of surface oxygen vacancies (OVs) near the Ag cluster (Supplementary Fig. 26c-f). Of note, the optimized mode with four adjacent OVs shows a more negative formation energy of −4.80 eV than that of the non-covalent contact model (−2.97 eV) (Fig. 2h), demonstrating that the Ag NPs covalently bonded to the MXene contribute significantly to stabilizing the metal-support interface. Furthermore, the electron density of the surface Ti atoms in the optimal Ti_3_C_2_O_2_@Ag is significantly higher than that in Ti_3_C_2_O_2_ and Ti_3_C_2_O_2_/Ag (Supplementary Fig. 27), confirming the electron redistribution between Ag and MXene, consistent with the XPS results. Overall, both experimental and theoretical findings support a structural picture in which embedded Ag atoms bind to the HMX lattice, while outer Ag species coordinate with surface O atoms, thereby stabilizing the heterointerface and enhancing electron transport efficiency.

### Electrochemical performance

**Fig. 3:**
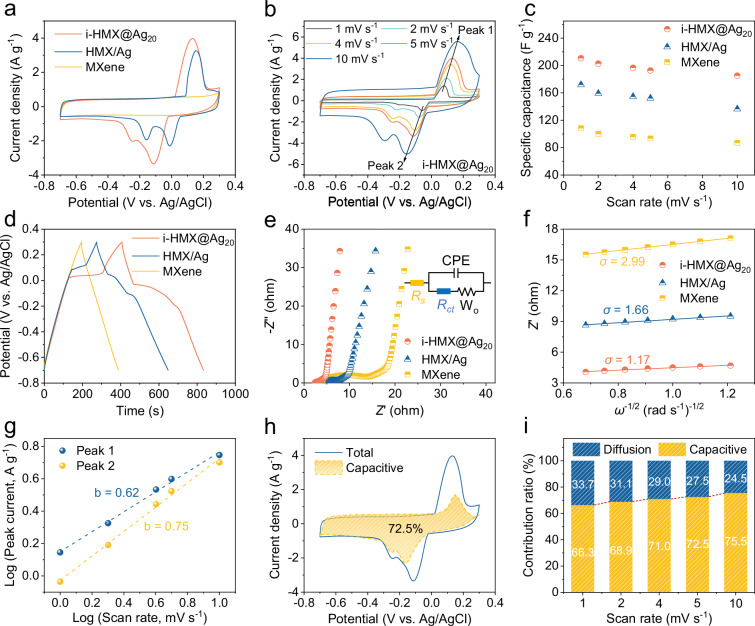
Electrochemical performance tests of the film electrodes. **a** Cyclic voltammetry (CV) curves at 5 mV s^−1^. **b** CV curves of i-HMX@Ag_20_ film at different scan rates. **c** Specific capacitance based on CV curves at different scan rates. **d** Galvanostatic charge-discharge (GCD) profiles at 0.5 A g^−1^. **e** Electrochemical impedance spectroscopy (EIS) Nyquist plots (inset is the equivalent circuit diagram) and **f** the corresponding fitting results for *Z*’ and *ω*^−1/2^ in the low-frequency region (linear fit, R^2^ is 0.9981, 0.9884, and 0.9782 for MXene, HMX/Ag, and i-HMX@Ag_20_, respectively). **g** Determination of b-value of i-HMX@Ag_20_ film, for the logarithm of anodic and cathodic peak currents versus the logarithm of scan rate (linear fit, R^2^ = 0.9941 for peak 1 and 0.9933 for peak 2). **h** The capacitive-controlled contribution of i-HMX@Ag_20_ film at 5 mV s^−1^. **i** The contribution ratios of capacitive and diffusion of i-HMX@Ag_20_ film at various scan rates.

The electrochemical properties of MXene, HMX/Ag, and i-HMX@Ag_*x*_ films were assessed in a three-electrode system, using 1 M NaCl aqueous solution as the electrolyte. Cyclic voltammetry (CV) was performed over a potential window of −0.7 to 0.3 V (vs. Ag/AgCl electrode), as depicted in Fig. 3a. MXene electrode exhibits a quasi-rectangular shape of CV curve without distinct redox peaks, suggesting the characteristic pseudocapacitive charge storage behavior^48^. In addition to the pseudocapacitive signal, the CV curves of both HMX/Ag and i-HMX@Ag_*x*_ electrodes manifest a pair of distinct redox peaks at approximately −0.1 V/0.1 V (vs. Ag/AgCl electrode) (Fig. 3a and Supplementary Fig. 28a), corresponding to the reversible transformation between Ag and AgCl^31^. Notably, the i-HMX@Ag_20_ electrode achieves the largest peak current density among the samples, reflecting its enhanced Cl^−^ storage capability due to the optimal metal-support coordination interface. The minor reduction peak observed at more negative potentials is attributed to side reactions, which is consistent with the previous study^21,22,30^. This peak is significantly suppressed in i-HMX@Ag_20_ compared to HMX/Ag, demonstrating its enhanced structural robustness against such parasitic processes. Moreover, the well-defined redox peaks progressively broaden and shift to higher potentials with increasing scan rate (Fig. 3b and Supplementary Fig. 29). This phenomenon can be attributed to the increased polarization and diffusion resistance at elevated scan rates^49^. Across a range of scan rates, the i-HMX@Ag_20_ electrode consistently delivers the largest specific capacitance, reaching 210.6 F g^−1^ at 1 mV s^−1^ and significantly surpassing the values of HMX/Ag (171.8 F g^−1^) and MXene (108.3 F g^−1^) (Fig. 3c and Supplementary Fig. 28b). The galvanostatic charge-discharge (GCD) profiles of i-HMX@Ag_*x*_ and HMX/Ag present quasi-symmetric triangular shapes and obvious charging and discharging plateaus (Fig. 3d and Supplementary Fig. 28c), further validating the combined pseudocapacitive and battery-type Cl^−^ capture process. The i-HMX@Ag_20_ electrode maintains the longest discharge time across all current densities (Supplementary Fig. 30), underscoring its higher specific capacitance and showing high consistency with the CV analysis. Therefore, i-HMX@Ag_20_ was selected as the optimal representative of i-HMX@Ag_*x*_ to conduct the subsequent performance experiments.

Electrochemical impedance spectroscopy (EIS) was employed to investigate the charge transfer kinetics and ion transport processes of i-HMX@Ag_20_, HMX/Ag, and MXene electrodes. In the Nyquist plots, the i-HMX@Ag_20_ electrode exhibits a smaller quasi-semicircle radius at high frequencies and a steeper straight line at low frequencies (Fig. 3e)^50^. According to the fitted equivalent electric circuit (inset of Fig. 3e), the combined series resistance (*R*_s_) and the charge transfer resistance (*R*_ct_) of i-HMX@Ag_20_ (*R*_s_ = 2.39 Ω, *R*_ct_ = 0.75 Ω) are markedly lower than those of HMX/Ag (*R*_s_ = 5.03 Ω, *R*_ct_ = 2.21 Ω) and MXene (*R*_s_ = 3.34 Ω, *R*_ct_ = 11.39 Ω) (Supplementary Table 3), demonstrating its enhanced electron transport behavior arising from the synergistic combination of an expanded interlayer spacing, a well‑developed porous network, and the interfacial coordination mode^51^. In the low-frequency region, the Warburg factor (*σ*), obtained from the linear slope of *Z*’ against *ω*^−1/2^ (Fig. 3f), is substantially lower for i-HMX@Ag_20_, implying reduced ion diffusion resistance within the electrode^52^. The quantified ion diffusion coefficient of i-HMX@Ag_20_ (2.88 × 10^−9^ cm^2^ s^−1^) is much higher than that of HMX/Ag (1.43 × 10^−9^ cm^2^ s^−1^) and MXene (4.41 × 10^−10^ cm^2^ s^−1^), validating the fast ion transport kinetics. To unveil the underlying mechanism of electrochemical kinetics for i-HMX@Ag_20_ electrode, the logarithmic relationship between current density and scan rate for the redox peaks is presented in Fig. 3g. The obtained b values for the anodic (peak 1) and cathodic (peak 2) peaks are 0.62 and 0.75, respectively, indicative of the compromise between capacitive-controlled and diffusion-controlled processes^53^. Given that the CV curve of i-HMX@Ag_20_ is approximately equal to the rectangular-like shape of MXene plus the redox transformation peaks of Ag/AgCl (Fig. 3a), this mixed electrochemical behavior originates from the genuine faradaic Ag/AgCl conversion coupled with the intrinsic capacitive contribution of the MXene support, rather than from any overestimation such as pseudo-capacity amplification. Moreover, the capacitive contribution at 5 mV s^−1^ is calculated to be 72.5% (Fig. 3h) and increases from 66.3% to 75.5% as the scan rate rises from 1 to 10 mV s^−1^ (Fig. 3i). For comparison, we also evaluated the electrochemical kinetic behavior of pristine MXene and HMX/Ag (Supplementary Fig. 31). Pristine MXene exhibits b values of ca. 0.95 and a high capacitive contribution of 84.0% at 5 mV s^−1^, indicating predominantly surface-controlled charge-storage behavior. In contrast, HMX/Ag shows b values of 0.66 (anodic) and 0.82 (cathodic), with a capacitive contribution of 75.1% at 5 mV s^−1^, which is comparable to that of i-HMX@Ag_20_. These results indicate that both HMX/Ag and i-HMX@Ag_20_ display a mixed capacitive and diffusion-controlled behavior. The well-aligned HMX nanosheets facilitate rapid ion intercalation and electron transfer, providing an optimal microenvironment in which the covalently linked Ag branches act as specific active sites for efficient faradaic dechlorination. This elaborate functional synergy collectively contributes to the enhanced ion migration kinetics and charge storage efficiency of the i-HMX@Ag_20_ electrode.

To further verify the critical role of our in situ etching‑and‑reduction strategy in boosting electrochemical performance, we set up a control sample by introducing NaBH_4_, a strong reducing agent, into the synthesis of i-HMX@Ag_20_ while keeping all other parameters unchanged. Interestingly, TEM images of the NaBH_4_-treated sample (Supplementary Fig. 32a, b) still exhibit Ag NPs and some in‑plane holes, suggesting that the redox reaction between Ag^+^ and MXene still occurs. However, the UV‑Vis absorption peak at 774 nm shifts only to 779 nm (Supplementary Fig. 32c), compared to 800 nm for i-HMX@Ag_20_, indicating a much weaker interfacial interaction between Ag and MXene. The XRD pattern exhibits characteristic peaks of both metallic Ag and MXene (Supplementary Fig. 32d), indicating the successful formation of Ag NPs under the combined reduction effects of MXene and NaBH_4_. Notably, the Ti 2*p* XPS spectrum (Supplementary Fig. 32e) shows that the negative shift of the peaks is much smaller than that of the i‑HMX@Ag_20_ (Fig. 2a). Moreover, the proportion of Ag^+^ species in Ag 3*d* XPS spectrum of the NaBH_4_-treated sample is also much lower (Supplementary Fig. 32f). These results suggest that most Ag^+^ are rapidly reduced by NaBH_4_ into Ag NPs that physically adsorb onto the MXene surface, rather than forming covalent bonds. As a result, the NaBH_4_-treated sample exhibits smaller CV peak currents, lower specific capacitance (196.4 F g^−1^ at 1 mV s^−1^), and shorter GCD discharge times than i‑HMX@Ag_20_ (Supplementary Fig. 32g–i). These observations collectively demonstrate that our in situ etching-and-reduction strategy promotes the formation of covalent Ag−C/O bonds at the heterointerface, which cannot be achieved by simply adding an external chemical reductant. The controlled interfacial chemistry is essential for the enhanced electrochemical performance of the i‑HMX@Ag_20_ electrode.

### Dechlorination performance and practical assessment

**Fig. 4:**
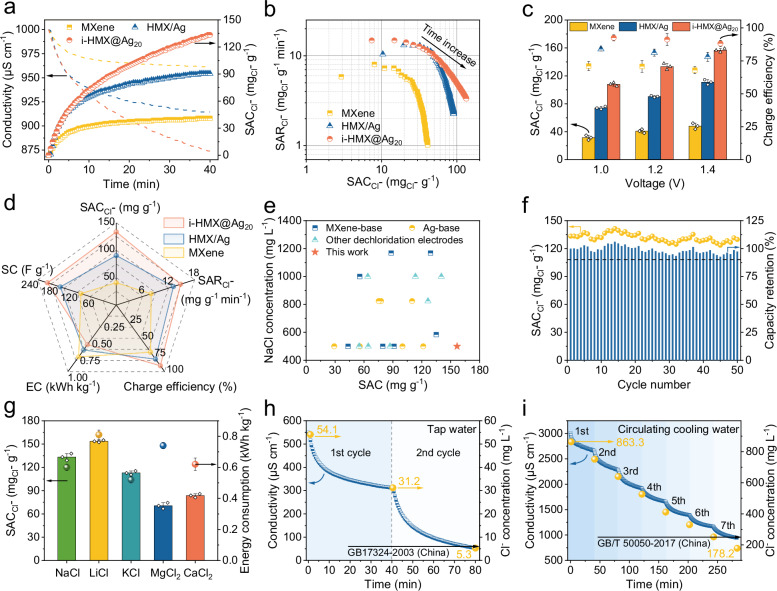
Comprehensive performance for electrochemical dechlorination. **a** Changes in conductivity and dechlorination capacity, and (**b**) capacitive deionization (CDI) Ragone plots of MXene, HMX/Ag, and i-HMX@Ag_20_ electrodes in a 500 mg L^−1^ NaCl solution at 1.2 V. **c** Dechlorination capacity and charge efficiency of MXene, HMX/Ag, and i-HMX@Ag_20_ electrodes at different voltages. **d** Radar chart comparison of MXene, HMX/Ag, and i-HMX@Ag_20_ electrodes in terms of dechlorination capacity (SAC), dechlorination rate (SAR), charge efficiency, energy consumption (EC), and specific capacitance (SC). **e** Comparison for dechlorination capacity of i-HMX@Ag_20_ with other faradaic dechlorination anodes. **f** Cyclic stability of i-HMX@Ag_20_ electrode within 50 cycles. Capacity retention is defined as the ratio of the dechlorination capacity at the nth cycle to that of the first cycle, expressed as a percentage. The capacity retention is 97.4% after 50 cycles. **g** Dechlorination capacity and energy consumption of i-HMX@Ag_20_ electrode in different metal chloride solutions with the concentration of 500 mg L^−1^. Changes in conductivity and Cl^−^ concentration of (**h**) actual tap water (**i**) and circulating cooling water treated with three tandem CDI cells. The Cl^−^ concentration in the treated tap water satisfies the hygienic standard (6 mg L^−1^) of bottled purified drinking water (GB17324-2003, China). The Cl^−^ concentration in the treated circulating cooling water meets the water quality specifications for makeup water (250 mg L^−1^) in open-circuit cooling water systems (GB/T 50050-2017, China). In (**c**) and (**g**), data are presented as mean ± standard deviation; error bars represent standard deviation; *n* = 3 independent systems.

The dechlorination performance of the as-made materials was evaluated in an asymmetric CDI cell, where each test sample (i-HMX@Ag_20_, HMX/Ag, and MXene films) served as the freestanding anode and MXene was employed as the matching cathode (Supplementary Fig. 33). Of note, the direct application of these films as electrodes eliminated the cumbersome steps involved in traditional electrode fabrication and the need for binders, saving both time and economic costs. As depicted in Fig. 4a, the gradually decreased conductivity of NaCl solution implies the continuous ion storage process driven by a constant voltage. Notably, i-HMX@Ag_20_ exhibits a much higher dechlorination capacity of 133.34 ± 4.4 mg g^−1^ than HMX/Ag (90.44 ± 1.2 mg g^−1^) and MXene (40.87 ± 2.7 mg g^−1^) when tested in a 500 mg L^−1^ NaCl solution under an applied voltage of 1.2 V. In the CDI Ragone plots (Fig. 4b), the occupied location of i-HMX@Ag_20_ in the upper-right region further reveals its higher dechlorination capacity and faster dechlorination rate (14.82 mg g^−1^ min^−1^)^54^. The performance enhancement of i-HMX@Ag_20_ underscores the beneficial impact of optimized interfacial compatibility and convenient ion channel on the desalination efficacy.

The impact of the applied voltage from 1.0 to 1.4 V on the desalination performance was explored. As shown in Fig. 4c, the dechlorination capacities of all samples show a positive correlation with the voltage due to accelerated ion migration by the intensified electric field. In particular, i-HMX@Ag_20_ consistently delivers the highest dechlorination capacity and charge efficiency across all constant voltages and achieves an ultrahigh dechlorination capacity of 156.63 ± 2.6 mg g^−1^ at 1.4 V. Although higher voltage usually brings about more side reactions and irreversible energy losses, i-HMX@Ag_20_ can consistently maintain its charge efficiency at a high level of around 90%, further highlighting the efficient electron transport obtained from its synergetic interfacial covalent linkage. Considering the fluctuating salt strengths of feedwaters in practical CDI applications, we performed the dechlorination capacity of our materials across various salt concentrations. As shown in Supplementary Fig. 34, i-HMX@Ag_20_ exhibits higher dechlorination capacity of 87.32 ± 1.5, 133.34 ± 4.4, and 161.58 ± 3.6 mg g^−1^ at 100, 500, and 1000 mg L^−1^, respectively. The excellent performance in both low- and high-concentration brine demonstrates the versatility and promising prospect of i-HMX@Ag_20_ for broad practical applications. The radar chart of the three electrodes (Fig. 4d) visually indicates that the i-HMX@Ag_20_ electrode displays enhanced comprehensive performance across all metrics, including dechlorination capacity, dechlorination rate, charge efficiency, energy consumption, and specific capacitance. In addition, further comparison with other materials highlights the significant advantage of i-HMX@Ag_20_ over the majority of the reported faradaic anodes, including Ag-based electrodes, MXene-based electrodes, and other dechlorination electrodes (Fig. 4e and Supplementary Table 4). The long-term cycling stability of the i-HMX@Ag_20_ electrode was further evaluated by a repetitive adsorption-desorption operation at 1.2 V in a 500 mg L^−1^ NaCl solution (Fig. 4f). As anticipated, i-HMX@Ag_20_ exhibits good cycling stability with capacity retention exceeding 90% over 50 cycles and negligible performance decay. Even extending the cycling test to 100 cycles, the i-HMX@Ag_20_ electrode can retain 78.6% of its initial capacity (Supplementary Fig. 35). Post‑cycling characterization was carried out to examine its structural changes. As shown in Supplementary Fig. 36a, the Ag NPs remain uniformly covered by the HMX nanosheets, with a surface morphology similar to the fresh electrode (Fig. 1h). In addition to the peaks of MXene and metallic Ag observed in the XRD spectrum (Supplementary Fig. 36b), relatively weak AgCl diffraction peaks were also detected, indicating a minor degree of irreversible phase transformation after prolonged cycling. Nevertheless, its layered film structure remains intact, with homogeneous distribution of Ti, Ag, and C elements throughout the cross‑section (Supplementary Fig. 36c). The Ag^+^ leaching concentration detected in the solution after 50 cycles was only 6.2 μg L^−1^, and after 100 cycles it remained as low as 7.1 μg L^−1^. Both values are far below the safety limit of 50 μg L^−1^ stipulated by the standards for drinking water quality (GB 5749-2022, China) and are well within the WHO guideline of 100 μg L^−1^. This extremely low Ag loss confirms that the covalent Ag−HMX interface not only anchors Ag species firmly but also prevents undesirable dissolution, thereby supporting both the structural stability and favorable environmental safety of the i-HMX@Ag_20_ electrode in long-term use. Moreover, the thickness of i-HMX@Ag_20_ film increased from 4.70 to 5.43 μm after Cl^−^ adsorption and recovered to 4.83 μm after desorption (Supplementary Fig. 37), demonstrating its excellent volumetric reversibility. The porous network of HMX nanosheets effectively buffers the volumetric expansion of Ag NPs, inhibiting the pulverization of Ag NPs and thus preserving the structural integrity and performance of the i-HMX@Ag_20_ electrode during long-term tests.

Given the complex nature of actual water, a systematic impact assessment of common coexisting cations (Li^+^, K^+^, Mg^2+^, Ca^2+^) on the dechlorination performance of the i-HMX@Ag_20_ electrode was conducted. The ion concentration was determined by conductivity-based calibration (Supplementary Fig. 38). The conductivity of different salt solutions gradually decreased during the adsorption stage over 40 min, and then rapidly recovered to its initial value during the desorption stage under a reverse voltage (Supplementary Fig. 39a–d). Intriguingly, the dechlorination performance of i-HMX@Ag_20_ in different metal chloride solutions exhibits a correlation with cation radius and carried charge number (Fig. 4g). An interesting trade-off between dechlorination capacity and energy consumption was observed among the monovalent cations. i-HMX@Ag_20_ shows the highest dechlorination capacity (153.63 ± 1.3 mg g^−1^) in LiCl solution, which is attributed to the superior intercalation capability of the smaller Li^+^ (0.76 Å) into the matching MXene cathode, thereby maximizing the charge balance for Cl^−^ adsorption. However, this came at the cost of the highest energy consumption per ion removed (0.81 ± 0.03 kWh kg^−1^) because the desolvation of the strongly hydrated Li^+^ (with the highest hydration energy) requires a higher applied voltage. In contrast, K^+^, with the largest ionic radius (1.38 Å) and the lowest hydration energy, exhibits the most energy-efficient removal (0.52 ± 0.02 kWh kg^−1^) but a lower capacity (112.91 ± 2.4 mg g^−1^). The significantly lower dechlorination capacity in divalent metal chloride solutions (MgCl_2_, CaCl_2_) compared to monovalent ones is primarily due to charge screening during EDL formation. The slightly higher capacity in CaCl_2_ over MgCl_2_ can be attributed to its marginally smaller hydrated radius and lower hydration energy, which reduce the kinetic barrier for ion approach and partial dehydration at the electrode interface. The charge efficiency exhibits a consistent pattern across the tested ions (Supplementary Fig. 39e, f), mirroring the impact of coexisting cations on dechlorination capacity and thereby substantiating the overall interpretation.

To assess the applicability of the i-HMX@Ag20 anode for dechlorination in various real water bodies, we constructed a multichannel tandem CDI system with three cascaded cells to treat tap water and circulating cooling water as representative low- and high-chloride waters, respectively. The incoming water was directly pumped into the series-connected CDI system for processing without any additional pretreatment. As depicted in Fig. 4h, the conductivity of tap water decreased from 550.1 to 312.2 μS cm^−1^ after the first run, and further dropped to 53.2 μS cm^−1^ after the second operation. The corresponding Cl^−^ concentration decreased from 54.1 to 5.3 mg L^−1^, satisfying the hygienic standard (6 mg L^−1^) of bottled purified drinking water (GB17324-2003, China). As for circulating cooling water, our CDI system after seven runs reduces its conductivity from 3010.1 to 946.2 μS cm^−1^, corresponding to a significant drop in Cl^−^ concentration from 863.3 to 178.2 mg L^−1^ and meeting the water quality specifications (250 mg L^−1^) for makeup water in open-circuit cooling water systems (GB/T 50050-2017, China) (Fig. 4i). The treated water with low Cl^−^ concentration can be repeatedly recycled while preventing scaling and pipeline corrosion. Accordingly, the i-HMX@Ag_20_-based dechlorination system demonstrates significant application potential for treating actual water with a wide range of Cl^−^ concentrations and complex water qualities.

### Enhancement mechanism

**Fig. 5:**
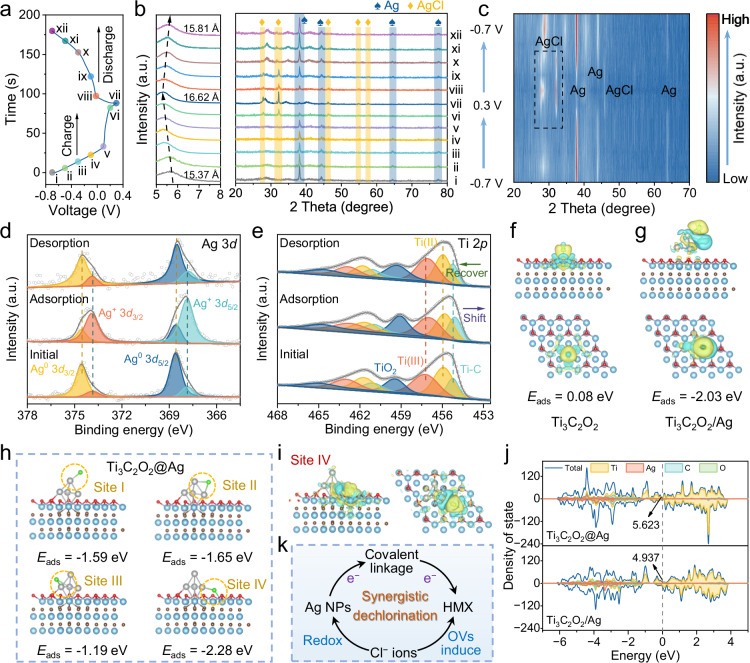
Dechlorination mechanism analysis by ex situ characterizations and theoretical calculations. **a** The representative steps i to xii of one consecutive GCD process for the i-HMX@Ag_20_ electrode at 2 A g^−1^ in 1 M NaCl solution. **b** Ex situ XRD patterns of the i-HMX@Ag_20_ electrode under various GCD stages and (**c**) their corresponding contour maps. **d** High-resolution Ag 3*d* XPS spectra and **e** Ti 2*p* XPS spectra of i-HMX@Ag_20_ after Cl^−^ adsorption and regeneration. Differential charge density diagrams of (**f**) Ti_3_C_2_O_2_ and **g** Ti_3_C_2_O_2_/Ag after Cl adsorption, where cyan and yellow represent charge depletion and charge accumulation, respectively. *E*_ads_ is the adsorption energy. **h** The side view of adsorption configurations for a Cl atom on Ti_3_C_2_O_2_@Ag with different active sites. **i** Differential charge density diagrams of Ti_3_C_2_O_2_@Ag after Cl adsorption. **j** Calculated total and partial density of states for Ti_3_C_2_O_2_/Ag and Ti_3_C_2_O_2_@Ag. **k** Schematic diagram of the synergistic dechlorination process for i-HMX@Ag_20_.

To pinpoint the Cl^−^ capture mechanism of the i-HMX@Ag_20_ electrode, ex situ electrochemical XRD analysis was performed to monitor the structural evolution during the charge/discharge process (Fig. 5a–c). The interlayer spacing of the i-HMX@Ag_20_ film expanded from 12.72 Å in a dry state (Figs. 1f) to 15.37 Å in a wet state due to the hydration swelling effect, while the well-defined layered structure was retained. Upon charging to 0.3 V (steps i to vii), the diffraction peak corresponding to the (002) plane of MXene shifted progressively from 5.7° to 5.3°, indicative of a continuous interlayer expansion from 15.37 to 16.62 Å due to Cl^−^ intercalation. Concurrently, the progressive attenuation of the Ag diffraction peaks alongside the growing intensity of the AgCl peaks (Fig. 5b, c) marks the substantial phase transition from Ag to AgCl following Cl^−^ capture in the i-HMX@Ag_20_ electrode, suggesting the dominated faradaic nature of the charge storage. On the contrary, upon discharging to −0.7 V (steps vii to xii), the interlayer spacing shrank back to its original value of 15.81 Å, and the AgCl was gradually reduced back to metallic Ag. These XRD findings reveal a coupled process of reversible Cl^−^ intercalation/deintercalation within the HMX galleries and Ag/AgCl redox transformation during the charge/discharge cycling of the i-HMX@Ag_20_ electrode.

Ex situ XPS was conducted to analyze the chemical valence evolution of the i-HMX@Ag_20_ electrode during the dechlorination/regeneration process. As illustrated in Fig. 5d, the proportion of Ag^+^ species increased significantly after Cl^−^ adsorption, which is attributed to the formation of AgCl, and then reverted to its initial state upon desorption due to electrochemical reduction. In the high-resolution Ti 2*p* XPS spectra (Fig. 5e), the characteristic peaks of Ti−C, Ti (II), and Ti (III) exhibited a negative shift after Cl^−^ adsorption and subsequently recovered to the initial peak positions upon desorption. This reversible peak shift confirms directional electron transfer, where electrons are donated from Ag NPs to HMX during charging and returned during discharging, restoring the interfacial charge state. These results are in good agreement with the ex situ XRD analysis, jointly corroborating the Cl^−^ capturing mechanism based on synergistic ion intercalation and faradaic reaction of Ag + Cl^−^ ⇌ AgCl + e^−^ in the i-HMX@Ag_20_ electrode. It should be noted that while completely decoupling the faradaic and capacitive contributions is difficult, the much higher capacity of i-HMX@Ag_20_ than pristine MXene, the reversible Ag/AgCl phase transition observed by ex situ XRD (Fig. 5b, c), and the high charge efficiency (~90%) collectively confirm that the faradaic reaction is the dominant Cl^−^ removal pathway.

To further elucidate the origin of the enhanced dechlorination performance, the charge distributions and Cl adsorption processes of the samples were comparatively analyzed by DFT calculations. The optimized configurations of Ti_3_C_2_O_2_, Ti_3_C_2_O_2_/Ag, and Ti_3_C_2_O_2_@Ag containing four OVs (Fig. 2h and Supplementary Fig. 26) were used to investigate the adsorption behavior of Cl atoms. As presented in Supplementary Fig. 40, the Cl atom preferentially adsorbs on the surface O sites of Ti_3_C_2_O_2_ and on the Ag cluster of Ti_3_C_2_O_2_/Ag. The differential charge density diagrams demonstrate the charge accumulation surrounding the adsorbed Cl atom and the charge depletion at the corresponding adsorption sites (Fig. 5f, g)^8^. The adsorption energy (*E*_ads_) of a Cl atom on Ti_3_C_2_O_2_ is 0.08 eV, indicative of weak interactions between pristine MXene and Cl. This phenomenon can be attributed to the electrostatic repulsion from the negatively charged MXene surface. By contrast, Ti_3_C_2_O_2_/Ag exhibits a negative *E*_ads_ value of −2.03 eV, suggesting a spontaneous and exothermic adsorption process driven by the strong chemical affinity between Ag and Cl. Nevertheless, the charge recombination is largely confined around the Ag atoms, with minimal charge redistribution of the MXene support (Fig. 5g), thereby failing to leverage the excellent conductivity of the MXene substrate. Notably, the optimized Ti_3_C_2_O_2_@Ag model exhibits multiple stable adsorption configurations for Cl atom, including binding to a single Ag atom (site I), bridging between two Ag atoms (site II and III), and coordinating to both a Ag and a surface O atom (site IV) (Fig. 5h and Supplementary Fig. 41). Among these, the *E*_ads_ value at site IV ( − 2.28 eV) is substantially more negative than at sites distant from the Ag/MXene interface, reflecting the favorable Cl adsorption promoted by the covalent linkage and OVs synergy at the heterogeneous interface. In this case, the electron distribution is more delocalized in Ti_3_C_2_O_2_@Ag compared to Ti_3_C_2_O_2_ and Ti_3_C_2_O_2_/Ag, with both the nearby Ag atoms and the Ti atoms exhibiting pronounced charge depletion after Cl adsorption (Fig. 5i). Furthermore, charge redistribution within the MXene lattice indicates that Ag not only interacts directly with Cl but also transfers electrons through the heterogeneous interface to the MXene, which in turn participates in Cl^−^ binding. These findings are consistent with the ex situ XPS results, demonstrating that the covalent Ag−MXene interfacial coupling effectively facilitates electron transfer, thereby enhancing Cl^−^ adsorption. The density of states (DOS) calculations further show that both Ti_3_C_2_O_2_@Ag and Ti_3_C_2_O_2_/Ag retain metallic character, with a distinct nonbonding band of Ti projected density of states (PDOS) near the Fermi energy (Fig. 5j)^45^. Notably, Ti_3_C_2_O_2_@Ag possesses higher DOS at the Fermi level compared to Ti_3_C_2_O_2_/Ag, further indicating improved electronic conductivity and redistribution of surface charge density^32,55^.

Based on comprehensive experimental and theoretical analyses, we propose a synergistic dechlorination mechanism stemming from covalent interfacial linkage at the Ag/HMX heterointerface (Fig. 5k). The mechanism operates through two coupled pathways rooted in the heterogeneous interface of i-HMX@Ag_20_. First, Ag NPs with atomically dispersed Ag SAs as growth pivots form covalent crosslinks with the HMX surface. This interfacial configuration promotes efficient electron transfer from Ag species to the HMX support during charging, rendering the Ag surface electron-deficient and highly active for the faradaic reaction (Ag + Cl^−^ ⇌ AgCl + e^−^), thereby directly enhancing dechlorination efficiency. Moreover, the HMX matrix serves not only as a highly conductive substrate facilitating charge transfer but also provides abundant active sites. The in situ etching process during material synthesis generates rich OVs, which expose unsaturated Ti atoms and promote the enrichment and capture of Cl^−^ on the HMX surface. Remarkably, the adsorption sites between the Ag atoms and OVs exhibit high activity for Cl^−^ adsorption. This intrinsic synergy, where covalent interfacial engineering and defect chemistry are rationally coupled, transcends simple physical mixing and establishes a general design principle for high-performance electrochemical dechlorination. Economically, although the use of Ag inevitably increases material costs, our interfacial design strategy is inherently extensible to other hybrid systems, thereby providing a general design principle for developing high‑performance yet cost‑effective hybrid electrodes.

## Discussion

In summary, we initiated interfacial covalent crosslinking between Ag and HMX via an in situ etching-and-reduction method, thereby establishing interfacial charge redistribution and a built-in electric field across the Ag NP/HMX junction. This strategy relies on the spontaneous redox reaction between metal cations and the low‑valence Ti species on MXene, and can be extended to other noble metals, such as Pd. The elaborately tailor-made i-HMX@Ag_20_ electrode demonstrated a high dechlorination capacity of 156.63 ± 2.6 mg g^−1^ in a 500 mg L^−1^ NaCl solution at 1.4 V with good cyclic stability, outperforming most reported CDI anode materials. The series-connected system of three CDI cells achieved a notable practical dechlorination effect for actual tap water and circulating cooling water. The enhanced performance is attributed to the concurrent construction of a covalently linked interface and a spatially interconnected porous conductive network, which together accelerate electron and ion transport. The underlying dechlorination mechanism was elucidated through a combination of ex situ characterization and DFT calculations. The covalent crosslink facilitates directed electron transfer from Ag NPs to HMX support, creating an electron-deficient Ag surface that kinetically favors the faradaic dechlorination. Of note, the OVs generated in situ on the HMX surface strengthen Cl^−^ adsorption, underscoring the synergistic effect between covalent interfacial engineering and defect chemistry. The well-designed HMX nanosheets further contribute as pseudocapacitive buffer layers to facilitate Cl^−^ intercalation and alleviate the volumetric expansion of Ag NPs during cycling. This work demonstrates the advantages of covalent linkage at Ag/MXene heterogeneous interfaces, providing an interface regulation strategy to develop hybrid electrodes for electrochemical dechlorination.

## Methods

### Preparation of Ti_3_C_2_T_*x*_ MXene dispersion

Monolayer Ti_3_C_2_T_*x*_ MXene dispersion was synthesized according to the minimally intensive layer delamination (MILD) method^36,37^. In detail, 1.5 g of Ti_3_AlC_2_ MAX powder was slowly added into a mixture of 30 mL of 9 M HCl and 2.4 g of LiF under continuous stirring. After reaction for 36 h at 35 °C, the precipitate was repeatedly washed with deionized (DI) water and centrifuged at 11,652 × *g* for 20 min until the pH of the supernatant reached approximately 6.5. The slurry was then sonicated for 15 min under a N_2_ environment and an ice bath. The unexfoliated particles were removed by centrifugation at 466 ×*g* for 30 min. Finally, the purified Ti_3_C_2_T_*x*_ MXene supernatant was collected and stored at 0 °C–5 °C.

### Preparation of i-HMX@Ag_*x*_ dispersion

Typically, a certain volume of AgNO_3_ solution (0.5 mg mL^−1^) was added into 25 mL of Ti_3_C_2_T_*x*_ MXene dispersion (2 mg mL^−1^) under continuous stirring. After in situ etching and reduction for 70 min, the precipitate was collected by centrifugation and washing with DI water to remove unreacted ions. Subsequently, the i-HMX@Ag_*x*_ (*x* represents the mass percentage of AgNO_3_ in relation to MXene and is numerically equivalent to the volume of the added AgNO_3_ solution in mL) dispersion was obtained by scavenging surface impurities with L(+)-tartaric acid solution and rinsing with DI water.

### Preparation of HMX/Ag dispersion

First, 0.9 mL of H_2_O_2_ was dropped into 300 mL of Ti_3_C_2_T_*x*_ MXene dispersion (0.5 mg mL^−1^) under continuous stirring. After etching for 20 min, the HMX was harvested by centrifugation and washing with L(+)-tartaric acid solution and DI water, followed by redispersion in water at a concentration of 2 mg mL^−1^. Then, commercial Ag NPs were dispersed in water at a concentration of 0.5 mg mL^−1^ under sonication treatment. Finally, the HMX/Ag dispersion was prepared by mixing HMX and Ag dispersions with Ag constituting 12.7 wt.% of the total solute.

### Preparation of Ti_3_C_2_T_*x*_ MXene, i-HMX@Ag_*x*_, and HMX/Ag films

The prepared Ti_3_C_2_T_*x*_ MXene, i-HMX@Ag_*x*_, and HMX/Ag dispersions were subjected to vacuum filtration for the fabrication of flexible films. The mass of each film was controlled to approximately 20 mg by adjusting the volume of each dispersion.

### Electrochemical tests

CV, GCD, and EIS measurements were carried out using a CHI 760 electrochemical workstation (CH Instruments). All electrochemical tests used a three-electrode system with 1 M NaCl solution as electrolyte. The prepared films were cut into long strips with an effective contact area of 1.5 × 1.0 cm and then used as working electrodes. A platinum plate and an Ag/AgCl electrode (KCl solution-saturated) were used as the counter electrode and the reference electrode, respectively. The specific capacitance (*C*, F g^−1^) was calculated from CV curves according to the following equation:

1 $$C=\int \frac{{I}{{{\rm{d}}}}{V}}{2v\varDelta {Vm}}$$

where *I* (A) is the current density, *v* (mV s^−1^) is the scan rate, Δ*V* (V) is the potential window, and *m* (g) is the mass of the electrode material.

The relationship between the current density (*i*, A g^−1^) and scan rate (*v*, mV s^−1^) can be fitted by the following equations:

2 $$i={{{\rm{a}}}}{v}^{{{{\rm{b}}}}}$$

3 $$\log \left(i\right)={{{\rm{blog}}}}\left(v\right)+\log \left({{{\rm{a}}}}\right)$$

The capacitive and diffusion contribution can be quantified by equations:

4 $$i\left(V\right)={k}_{1}v+{k}_{2}{v}^{1/2}$$

5 $$i/{v}^{1/2}={k}_{1}{v}^{1/2}+{k}_{2}$$

where *k*_1_ and *k*_2_ are constants, *k*_1_*v* and *k*_2_*v*^1/2^ represent capacitive-controlled contribution and diffusion-controlled contribution, respectively.

The EIS test was conducted over a frequency range between 0.1-10^5 ^Hz with an open circuit application potential and an amplitude of 5 mV. The Cl^−^ ion diffusion coefficient (*D*, cm^2^ s^−1^) was calculated by using the following equations:

6 $$D=\frac{0.5{R}^{2}{T}^{2}}{{F}^{4}{c}^{2}{A}^{2}{\sigma }^{2}}$$

7 $${Z}_{{{{\rm{re}}}}}=\sigma {\omega }^{-0.5}$$

where *R* is the gas constant (8.314 J K^−1^ mol^−1^), *T* (K) represents the temperature, *F* is the Faraday constant (96485 C mol^−1^), *c* (mol cm^−3^) is the ion concentration, *A* (cm^2^) stands for the surface area of electrode, *σ* (Ω s^−0.5^) is the Warburg factor, *Z*_re_ (Ω) is the real part impedance, and *ω* (s^−1^) represents frequency.

### CDI tests

The CDI cell consisted of two acrylic support plates (5 × 5 × 1 cm), freestanding film electrodes, and ion exchange membranes (IEMs) located near the current collectors. Specifically, the cation exchange membrane (CMI-7000S) and the anion exchange membrane (AMI-7001S) were purchased from Membrane International Inc. in the USA. Before use, both of these IEMs needed to be immersed in a 3 ~ 5 wt.% NaCl solution for 24 h for hydration and swelling. The cell also featured a flow channel measuring 3 × 3 cm × 3 mm and multiple rubber gaskets. The as-prepared films with a diameter of 4 cm were directly pasted onto the conductive carbon paper as binder-free electrodes. During the CDI test, 50 mL of 500 mg L^−1^ NaCl solution was circulated through the system. The flow rate was kept at 40 mL min^−1^ by using a peristaltic pump. A conductivity meter (EC-400F, Shanghai INESA Scientific Instrument Co., Ltd.) was utilized to record the real-time conductivity of the NaCl solution. The conductivity can be converted to the corresponding NaCl concentration through mathematical calculations. In the practical applicability experiments, the tap water was directly supplied by the municipal source. The circulating cooling water was simulated by dissolving 0.83 g of NaCl, 0.46 g of CaCl_2_, 0.19 g of MgCl_2_·6H_2_O, and 0.27 g NaHCO_3_ in 1 L of DI water, where NaCl is the main source of Cl^−^, Ca^2+^ and Mg^2+^ provide the hardness of water, and NaHCO_3_ is a buffer to maintain the pH value of water at a slightly alkaline level. The salt adsorption capacity (SAC_Cl_-, mg_Cl_- g^−1^) and salt adsorption rate (SAR_Cl_-, mg_Cl_- g^−1^ min^−1^) of the electrodes were calculated according to the following equations:

8 $${{{{\rm{SAC}}}}}_{{{{{\rm{Cl}}}}}^{-}}=\frac{\left({C}_{0}-{C}_{{{{\rm{e}}}}}\right)V}{m}$$

9 $${{{{\rm{SAR}}}}}_{{{{{\rm{Cl}}}}}^{-}}=\frac{{{{{\rm{SAC}}}}}_{{{{{\rm{Cl}}}}}^{-}}}{t}$$

where *C*_0_ and *C*_e_ (mg L^−1^) are the initial and final Cl^−^ concentrations, respectively, *V* (L) is the volume of the salt solution, *m* (g) is the effective mass of the dechlorination electrode, and *t* (min) is the dechlorination time.

The charge efficiency (*Λ*) and energy consumption (*E*, kWh kg^−1^) were calculated according to the following equations:

10 $$\varLambda=\frac{\varGamma \times F}{\varSigma }$$

11 $$E=\frac{U\int I\left(t\right){{{\rm{d}}}}t}{{m}_{{{{\rm{NaCl}}}}}}$$

where *Γ* (mol g^−1^) is the dechlorination capacity, *F* is the Faraday constant (96485 C mol^−1^), and *Ʃ* (charge, C g^−1^) is the charge consumption and obtained by integrating current during the desalination process, *U* is the applied potential (V), *I*(*t*) is the current (A), and *m*_NaCl_ is the mass of removed NaCl.

## Supplementary information

Supplementary information

Transparent Peer Review file

## Source data

source data

## Data Availability

The data supporting the key findings of this study are available within the article and the Supplementary Information. All data generated in this study are provided in the Supplementary Information/Source Data file. Source data are provided with this paper.
