# Supplementary material for: Covalent Ag-holey MXene link at interface boosting electrochemical dechlorination
Source: Nat Commun. 2026 Jul 14;17:8642. doi: 10.1038/s41467-026-75560-1 (PMC13486493; doi:10.1038/s41467-026-75560-1)
Supplement: Supplementary file 1 [file 41467_2026_75560_MOESM1_ESM.pdf]

Supplementary Information

Covalent Ag-holey MXene link at interface boosting electrochemical dechlorination

Hao Zhang[1], Chenxu Liu[1], Hao Wang[1], Xin Ma[1], Ruochen Wang[1], Jiansheng Li[2]*, Jiayin Yuan[3]*, Miao Zhang[1]*

[1]School of Chemistry, Xi'an Jiaotong University, Xi'an, 710049, P.R. China

[2]Key Laboratory of Jiangsu Province for Chemical Pollution Control and Resources Reuse, School of Environmental and Biological Engineering, Nanjing University of Science and Technology, Nanjing, 210094, P.R. China

[3]Department of Chemistry, Stockholm University, Stockholm, 10691, Sweden

*Corresponding author.

E-mail: lijsh@njust.edu.cn; jiayin.yuan@su.se; miaozhangchem@xjtu.edu.cn

This Supplementary Information document includes:

Supplementary Notes

Supplementary Methods

Supplementary Figures 1-41

Supplementary Tables 1-4

Supplementary References

Supplementary Notes

Chemicals

Titanium aluminum carbide powder (Ti_3AlC_2 MAX, 400 meshes) was purchased from Laizhou Kai Kai Ceramics Materials Co., Ltd. Hydrochloric acid (HCl, 37%), lithium fluoride (LiF, 99%), hydrogen peroxide (H_2O_2, 30%), and silver nitrate ($AgNO_3$, ≥ 99.8%) were purchased from Sinopharm Chemical Reagent Co., Ltd. L(+)-tartaric acid (> 99.0%) was purchased from Tokyo Chemical Industry Co., Ltd. Silver nanoparticles (Ag NPs, 99.5%, 60-120 nm) were purchased from Shanghai Macklin Biochemical Co., Ltd. NaCl (99.5%), KCl (≥ 99.5%), $MgCl_2 \cdot 6H_2O$ (≥ 99%), and $CaCl_2$ (≥ 97%) were purchased from Shanghai Aladdin Biochemical Technology Co., Ltd. All chemicals were used as received without further purification. Deionized (DI) water (18.25 MΩ·cm) was used throughout the experiment.

Supplementary Methods

Supplementary Method 1. Characterizations

Scanning electron microscopy (SEM) images were collected by using Gemini SEM 500 (ZEISS, German). The powder specimens were directly attached to the conductive adhesive tape. The film samples were first broken by liquid nitrogen and then attached to the conductive adhesive tape. Transmission electron microscopy (TEM) was performed on a Talos F200X instrument (Thermo Fisher, USA) at 200 kV. The specimens were mounted on a Cu grid (Lacey C only, TED PELLA, INC., USA) by dropping a drop of the diluted dispersion solution (0.01 mg mL^{-1}) and drying. High-angle annular dark-field scanning TEM (HAADF-STEM) and corresponding energy-dispersive X-ray spectroscopy (EDS) elemental mapping were performed on an Aberration-corrected transmission electron microscope (JEM-ARM200F NEOARM, JEOL, Japan) at 200 kV, equipped with a Silicon Drift Detector (SDD) electric cooling detector (windowless type, 100 square millimeters in size). Atomic force microscopy (AFM, Veeco Instruments, CA) was conducted by a Nanoscope V in tapping mode. ScanAsyst-air probe was used in all morphology measurements. The specimens were mounted on a mica plate by dropping a drop of the diluted dispersion solution (0.01 mg

mL^{-1}) and drying. All the images were processed using Nanoscope Analysis 2.0 software. X-ray diffraction (XRD, Bruker, D8 Advance, German) patterns were collected by using Cu Kα radiation (40 kV, 40 mA, λ = 1.5418 Å). The functional groups of the prepared films were explored using a Fourier transform infrared spectrometer (FTIR-850, Tianjin Gangdong Sci.&Tech. Co., LTD.) with a resolution of 1 cm^{-1} and a scanning frequency of 32. Raman spectra were recorded from 100 to 2000 cm^{-1} using InVia Qontor spectrometer (Renishaw). X-ray photoelectron spectroscopy (XPS, Thermo Fisher, Escalab 250XI, USA) spectra were conducted by using a monochromatic Al Kα X-ray source ($h\upsilon$ = 1486.6 eV). The water contact angle was measured on a goniometer (Krüss, DSA25, Germany) at ambient conditions. All the images were processed with the ADVANCE software. Zeta potential measurements were performed on a Zetasizer Nano ZS90 (Malvern, England) using a folding capillary sample cell (DTS1070). Ultraviolet-visible (UV-Vis) spectra were tested by a GENESYS 150 (Thermo Fisher, USA). The electrical conductivity of the films was obtained on a four-probe resistivity tester (RTS-8, 4Probes Tech Ltd.).

Supplementary Method 2. Details of characterizing the solid phase with X-ray absorption fine structure (XAFS) analyses

Ag K-edge analysis was performed with Si (111) crystal monochromators at the BL14W1 beamlines at the Shanghai Synchrotron Radiation Facility (SSRF) (Shanghai, China). Before the analysis at the beamline, samples were pressed into thin sheets with 1 cm in diameter and sealed using Kapton tape film. The XAFS spectra were recorded at room temperature using a 4-channel Silicon Drift Detector (SDD) Bruker 5040. Ag K-edge extended X-ray absorption fine structure (EXAFS) spectra were recorded in transmission mode. Negligible changes in the line-shape and peak position of Ag K-edge X-ray absorption near edge structure (XANES) spectra were observed between two scans taken for a specific sample. The XAFS spectra of these standard samples (Ag foil, Ag_2O, and AgO) were recorded in transmission mode. The spectra were processed and analyzed by the software codes Athena and Artemis.

Supplementary Method 3. Density functional theory (DFT) calculations

All calculations were carried out with the Vienna Ab-initio Simulation Package, employing the Perdew-Burke-Ernzerhof (PBE) functional within the generalized gradient approximation (GGA) for the exchange-correlation interactions. A plane-wave basis set with a cutoff energy of 450 eV was used, together with the projector-augmented wave (PAW) method to treat electron-ion interactions. The Ti_3C_2 (001) surface was represented by a p(4×4) supercell, and a vacuum layer of 20 Å was introduced along the z-direction to minimize interlayer coupling. This surface was fully saturated with oxygen to construct the $Ti_3C_2O_2$ configuration. A five-atom silver cluster was then deposited on $Ti_3C_2O_2$, yielding the $Ti_3C_2O_2$-Ag5 system. Furthermore, selected oxygen vacancies (OVs) were introduced on $Ti_3C_2O_2$, into which the Ag cluster was embedded, resulting in the $Ti_3C_2O_2$-Ag5-OVs structure. The total energy change between successive steps fell below 10^{-4} eV. A 3×3×1 k-point mesh was used for sampling the Brillouin zone. The DFT-D3 scheme was adopted to incorporate dispersion corrections. To address the self-interaction error in transition metals with localized d electrons (such as Ti in $Ti_3C_2O_2$-Ag5), the DFT + U approach was applied, setting the effective U parameter to 4.0 eV for Ti 3d states. The adsorption energy of Cl* was calculated as $E_{ads} = E_{total} - E_{slab} - E_{adsorbate}$, where E_{total}, E_{slab}, and $E_{adsorbate}$ denote the total energies of the adsorbate-surface system, the clean slab, and the isolated adsorbate, respectively. VASP 5.4.4 version and VESTA version 3 were used for DFT calculations.

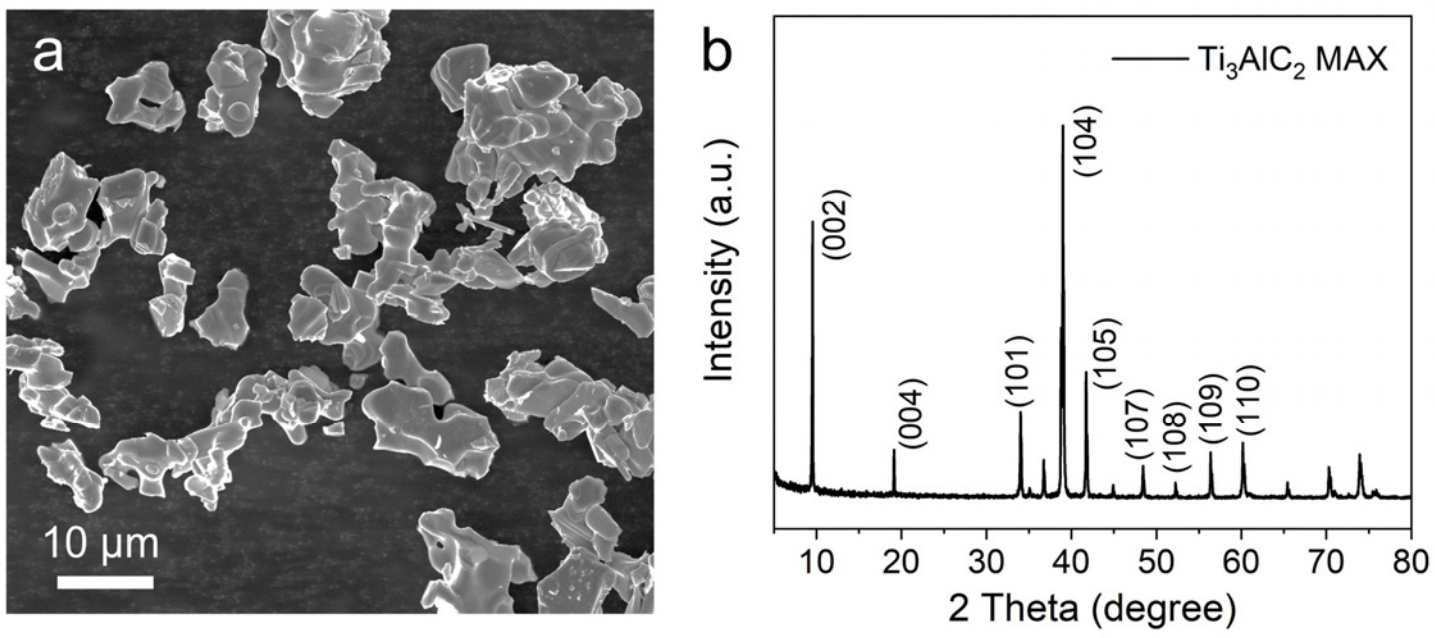

Supplementary Fig. 1. Characterization analysis of MAX. (a) SEM image and **(b)** XRD pattern of Ti_3AlC_2 MAX.

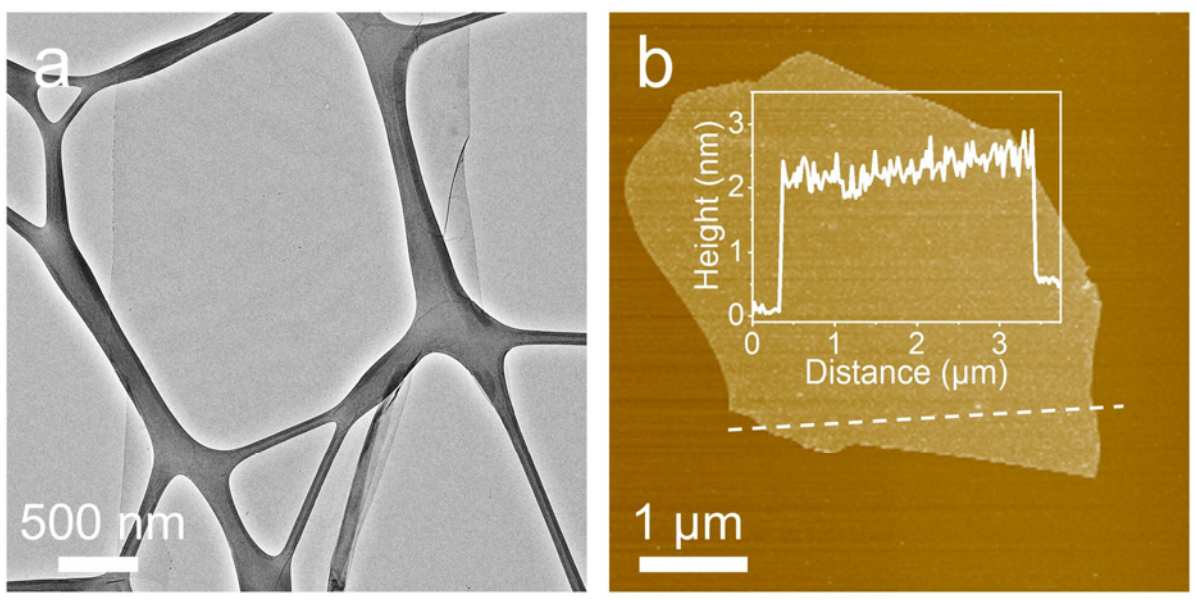

Supplementary Fig. 2. **Pristine MXene nanosheet. (a)** TEM and **(b)** AFM images of monolayer $Ti_3C_2T_x$ MXene nanosheet.

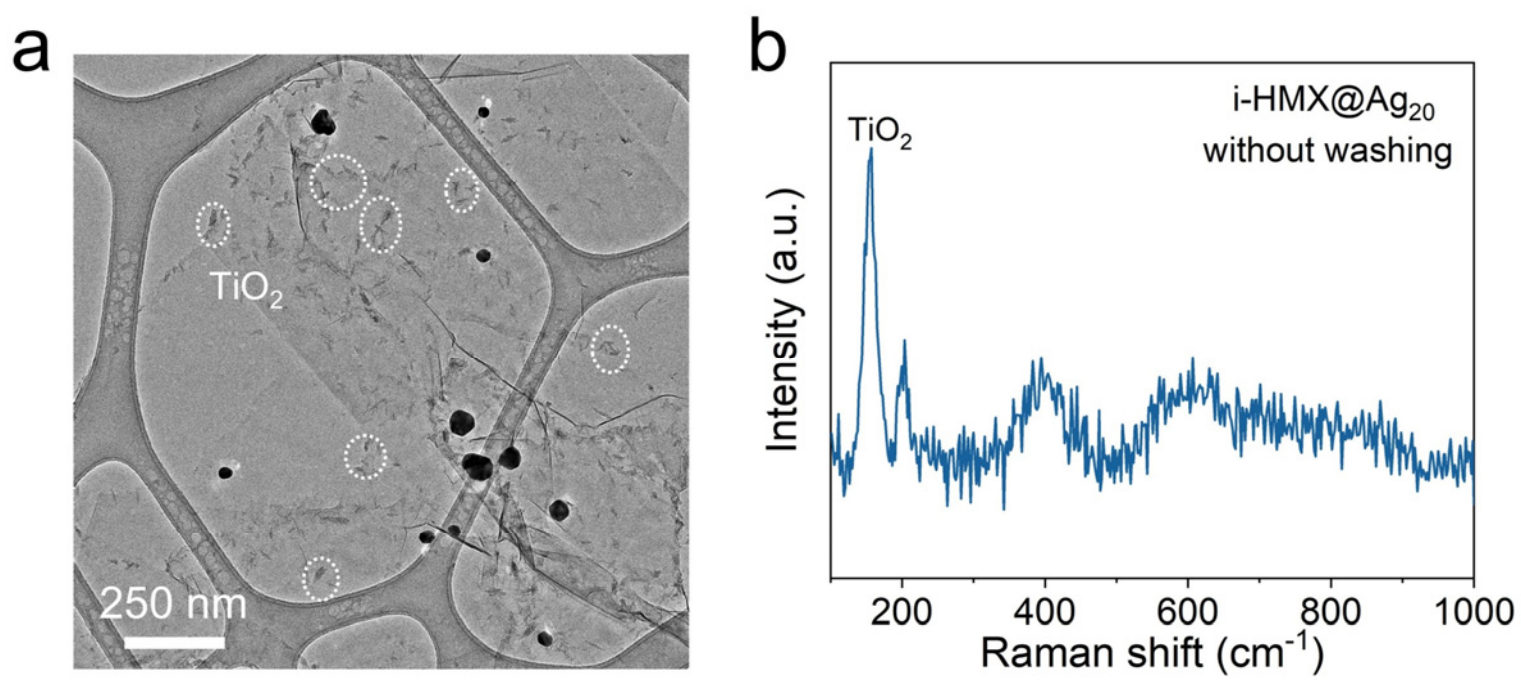

Supplementary Fig. 3. The morphology before acid washing. (a) TEM image and **(b)** Raman spectrum of i-HMX@Ag_{20} before washing with L(+)-tartaric acid.

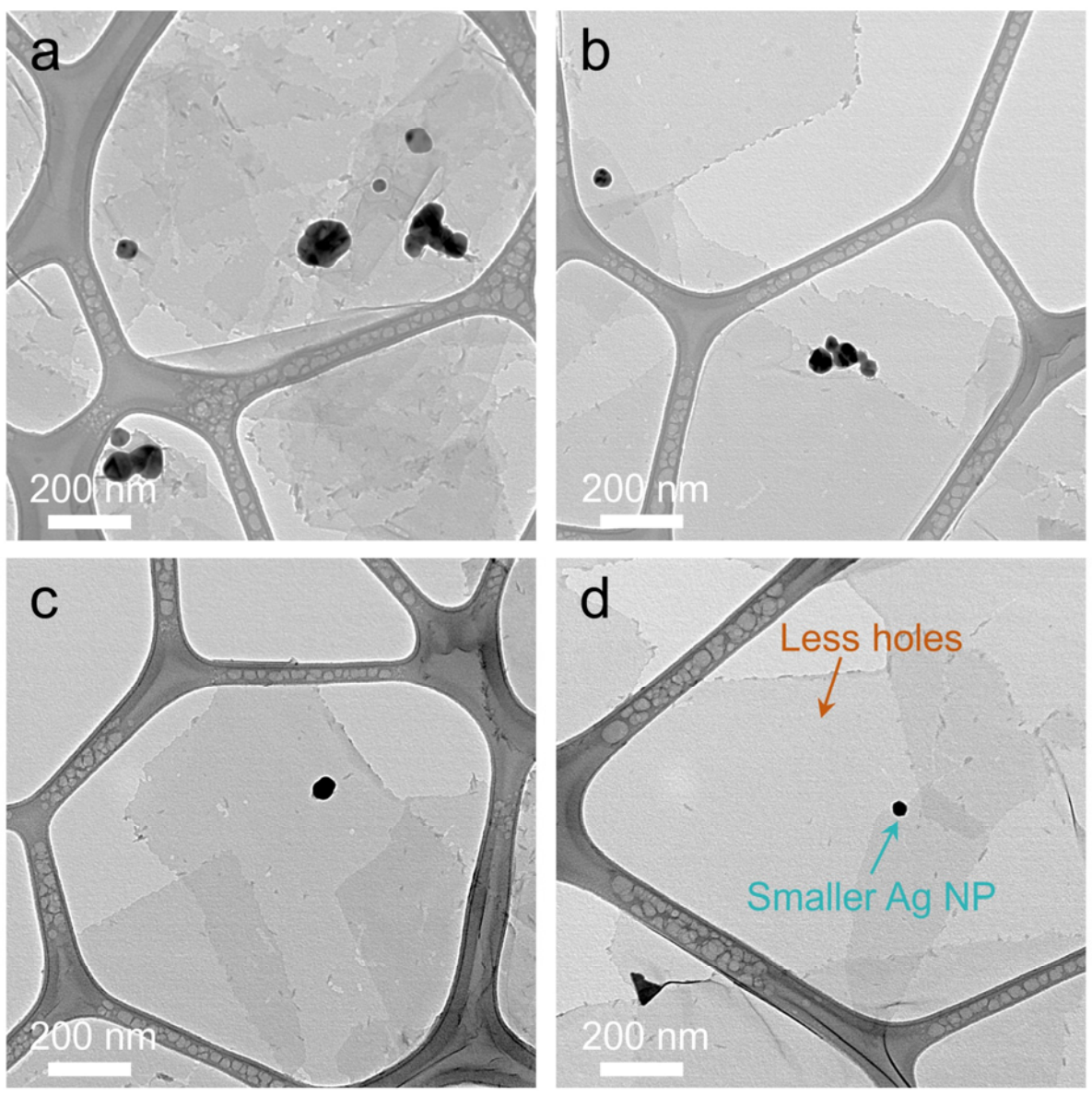

Supplementary Fig. 4. Changes of Ag NPs and nanoholes. TEM images of **(a)** i-HMX@Ag_{30}, **(b)** i-HMX@Ag_{10}, **(c)** i-HMX@Ag_{5}, and **(d)** i-HMX@Ag_{1} nanosheets.

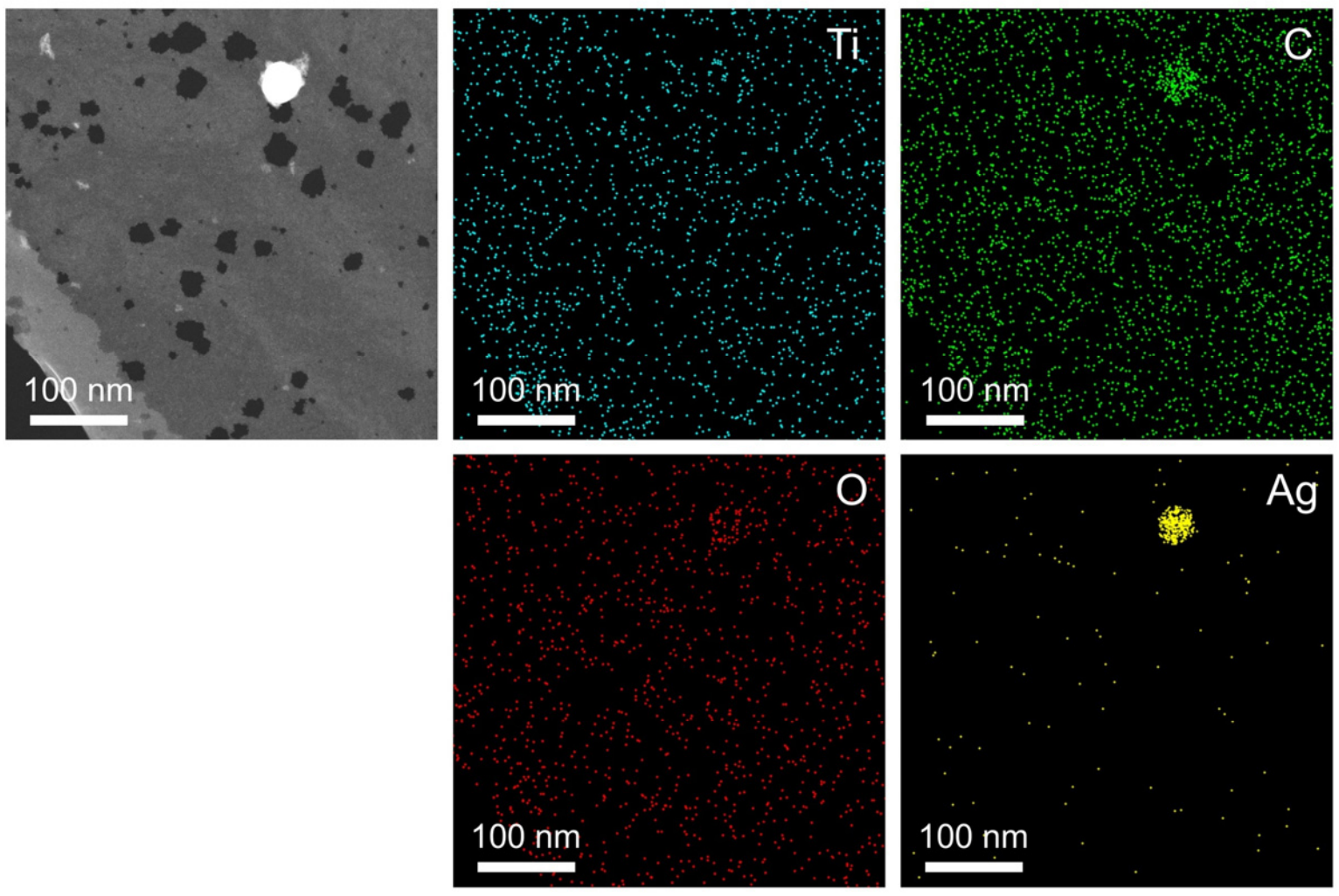

Supplementary Fig. 5. Elemental distribution analysis. HAADF-STEM and corresponding EDS elemental mapping images for elements Ti, C, O, and Ag of i-HMX@Ag_{20} nanosheet.

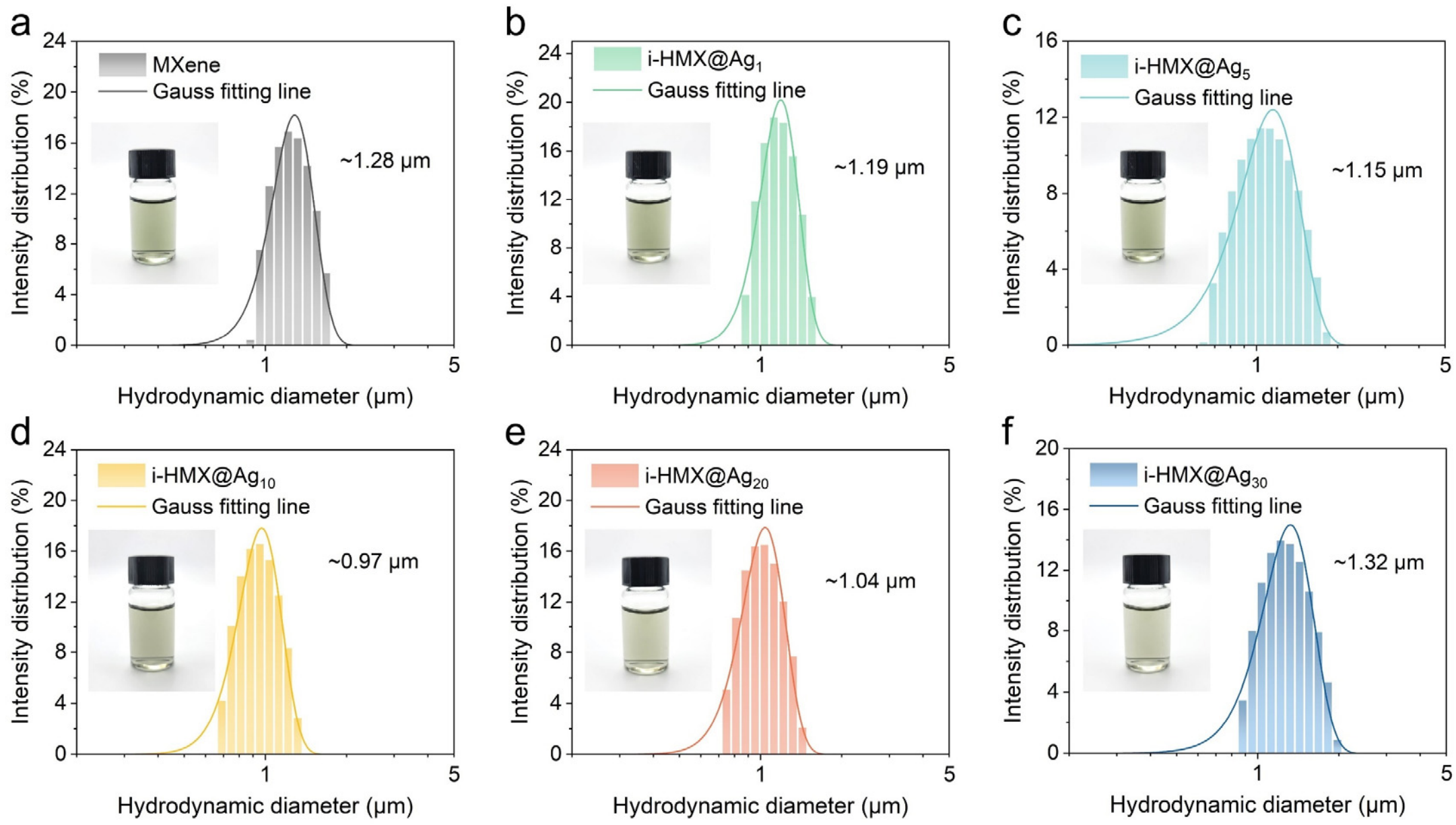

Supplementary Fig. 6. Size of nanosheets. Size distributions of **(a)** MXene, **(b)** i-HMX@Ag_1, **(c)** i-HMX@Ag_5, **(d)** i-HMX@Ag_{10}, **(e)** i-HMX@Ag_{20}, and **(f)** i-HMX@Ag_{30} nanosheets. All plots were conducted by Gauss fitting; R^2 is 0.9705, 0.9794, 0.9654, 0.9765, 0.9753, and 0.9678 in **(a)** to **(f)**, respectively. Inset: digital photographs of the corresponding dispersions. The concentration of all the test dispersions is 0.01 mg mL^{-1} and the pH value is 7.

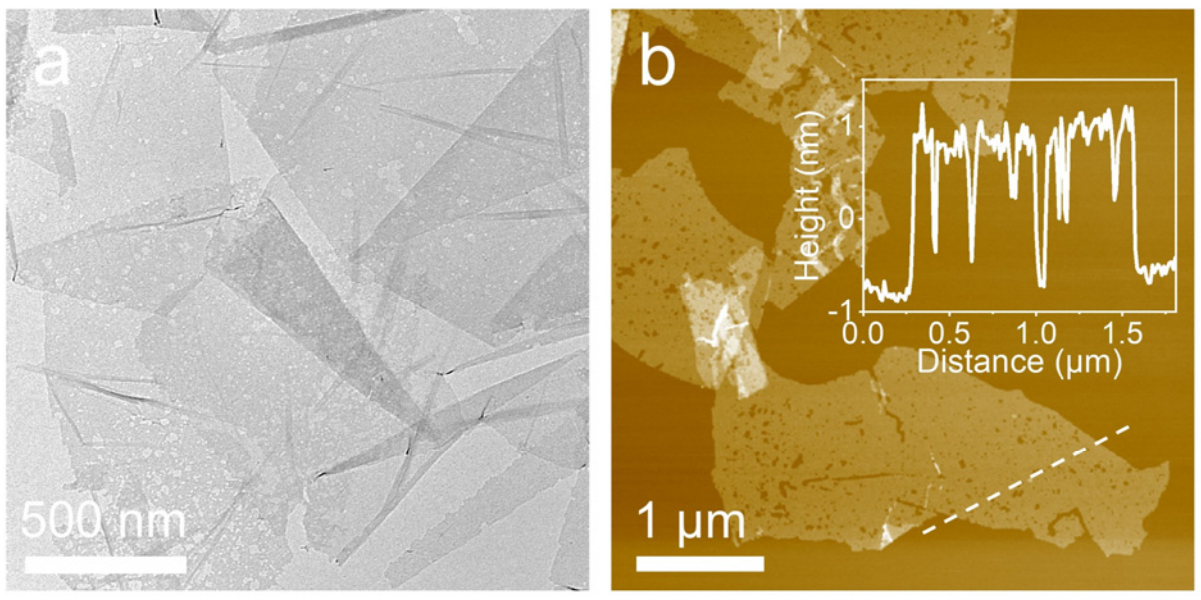

Supplementary Fig. 7. Morphology of HMX. **(a)** TEM and **(b)** AFM images of monolayer HMX nanosheets. Inset is the height of a single nanosheet.

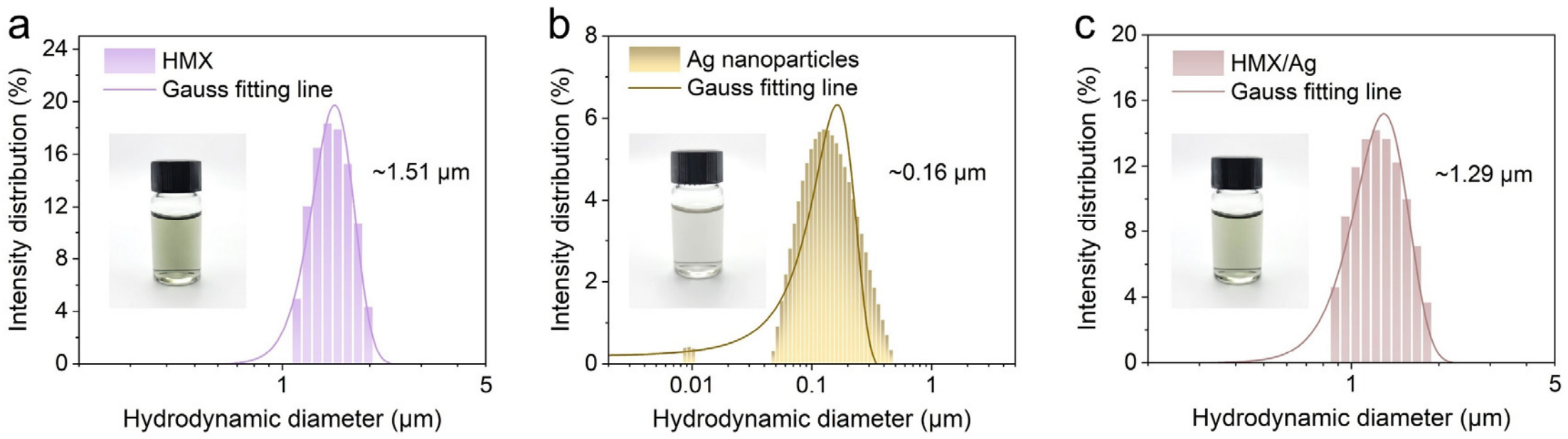

Supplementary Fig. 8. Size of HMX, Ag NPs, and HMX/Ag. Size distributions of **(a)** HMX, **(b)** Ag NPs, and **(c)** HMX/Ag. All plots were conducted by Gauss fitting; R^2 is 0.9784, 0.8804, and 0.9654 in **(a)** to **(c)**, respectively. Inset: digital photographs of the corresponding dispersions. The concentration of all the test dispersions is 0.01 mg mL^{-1} and the pH value is 7.

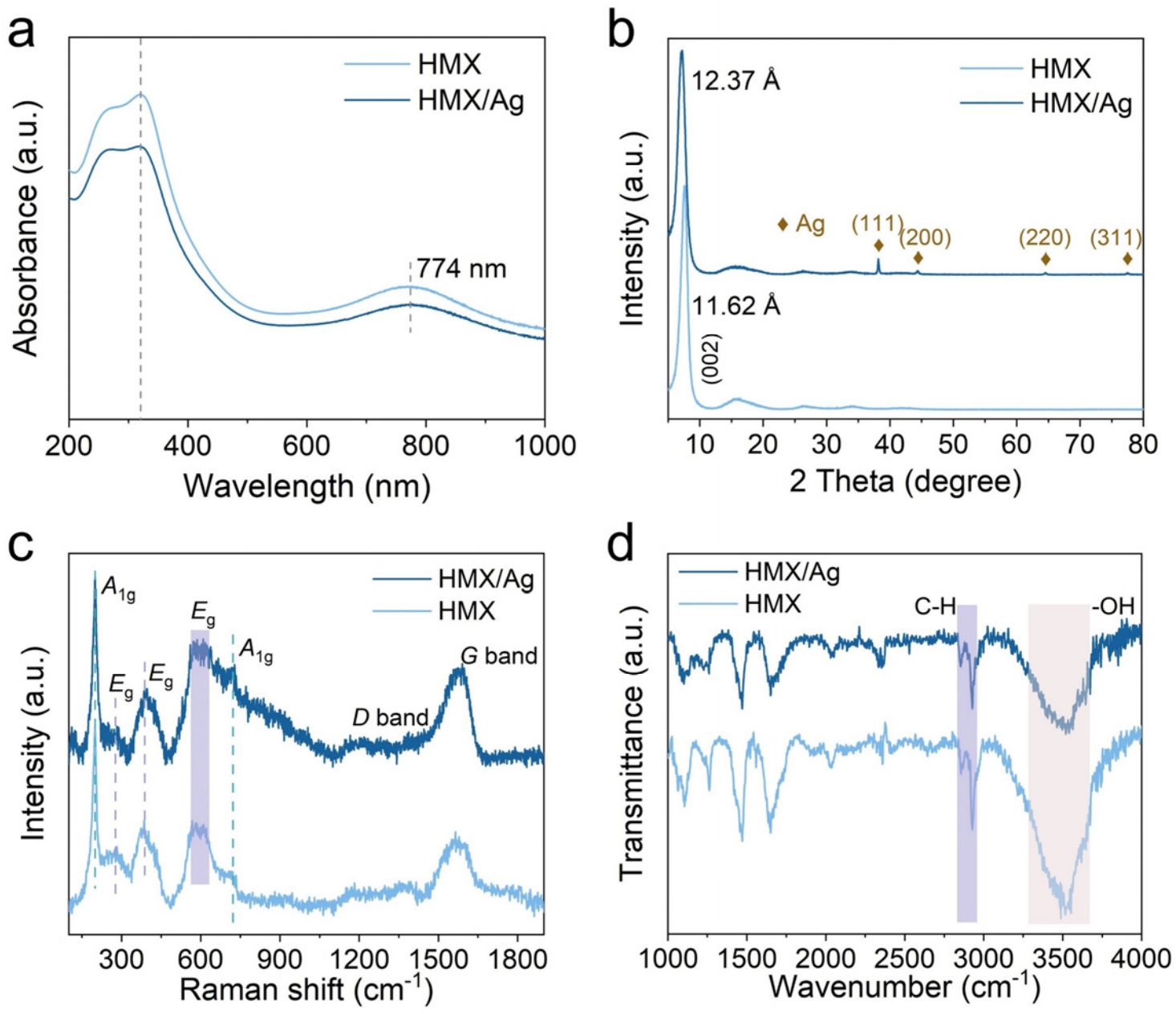

Supplementary Fig. 9. Characterization analysis of HMX and HMX/Ag. (a) UV-Vis absorption spectra, **(b)** XRD patterns, **(c)** Raman spectra, and **(d)** FT-IR spectra of HMX and HMX/Ag.

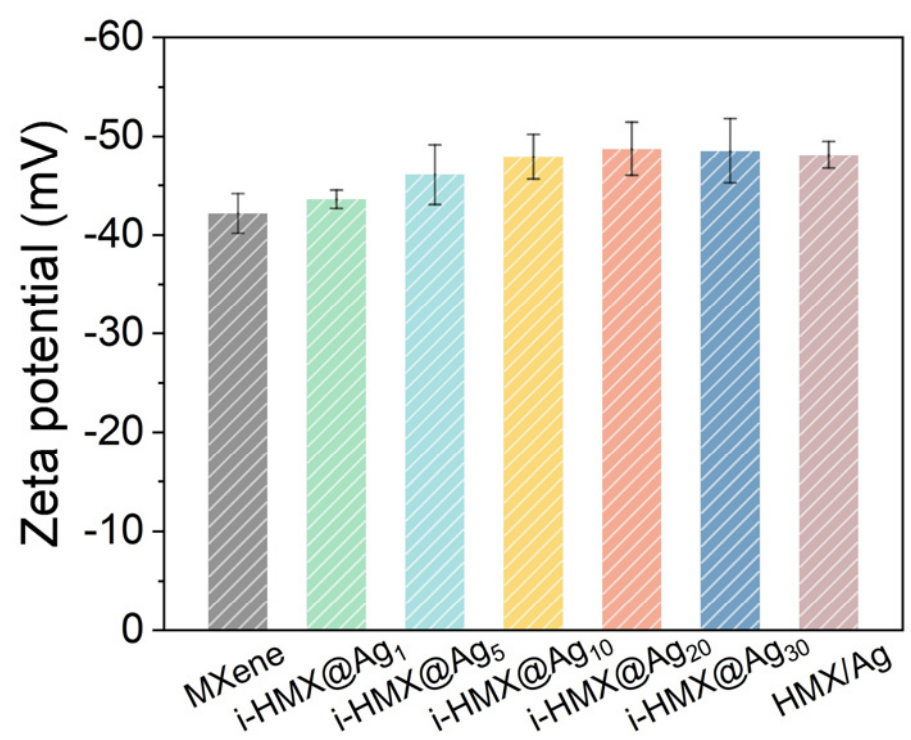

Supplementary Fig. 10. Surface potential analysis. Zeta potential of different samples. The concentration of all the test dispersions is 0.01 mg mL^{-1} and the pH value is 7. Data are presented as mean ± standard deviation; error bars represent standard deviation; n = 100 processed runs.

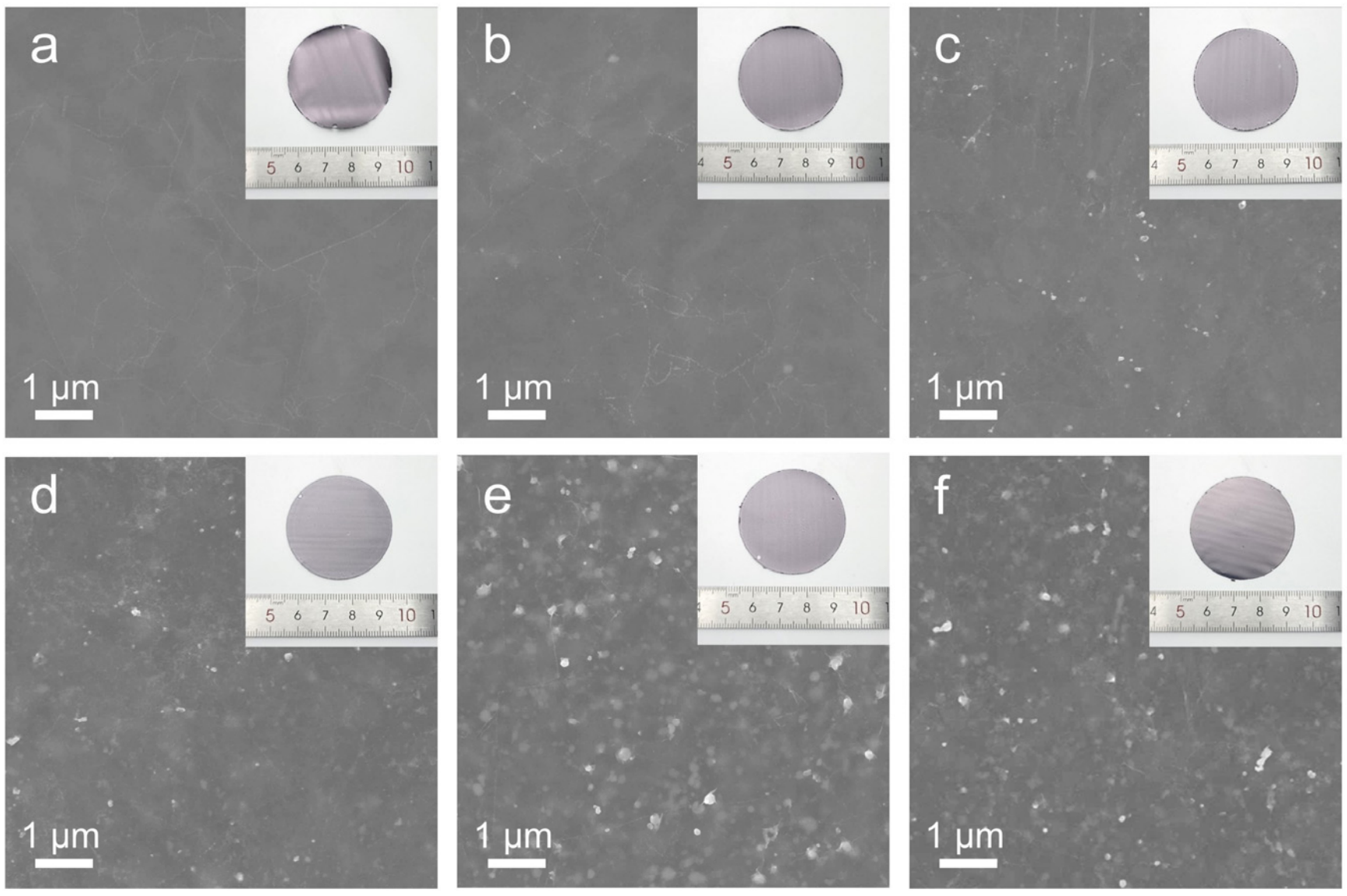

Supplementary Fig. 11. Surface morphology of films. Digital photographs (inset) and surface SEM images of **(a)** MXene, **(b)** i-HMX@Ag_1, **(c)** i-HMX@Ag_5, **(d)** i-HMX@Ag_{10}, **(e)** i-HMX@Ag_{30}, and **(f)** HMX/Ag films.

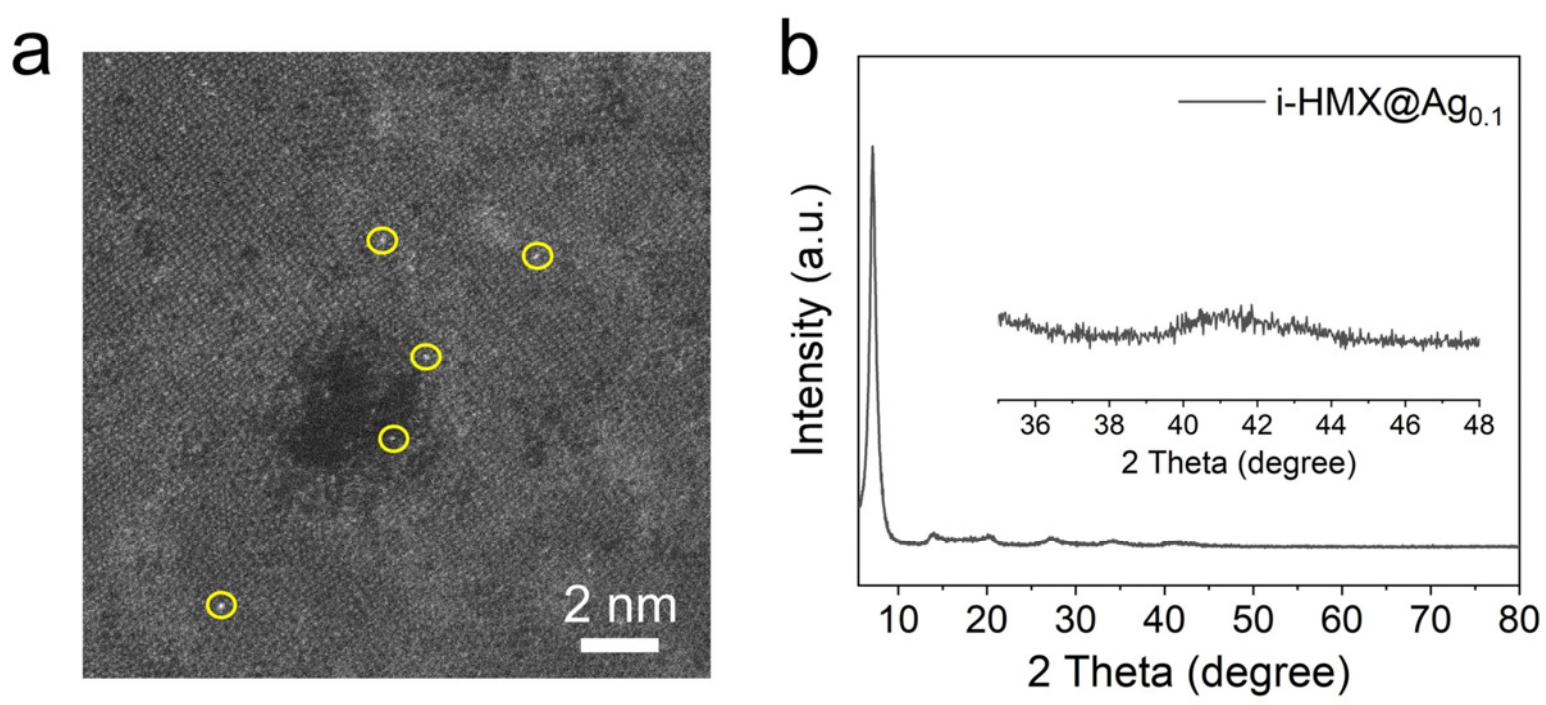

Supplementary Fig. 12. Characterization analysis in a low Ag content. (a) HAADF-STEM and **(b)** XRD pattern of i-HMX@$Ag_{0.1}$. The yellow circles in **(a)** represent Ag SAs. The inset in **(b)** is the partial enlarged XRD plots from 35 to 48 degree.

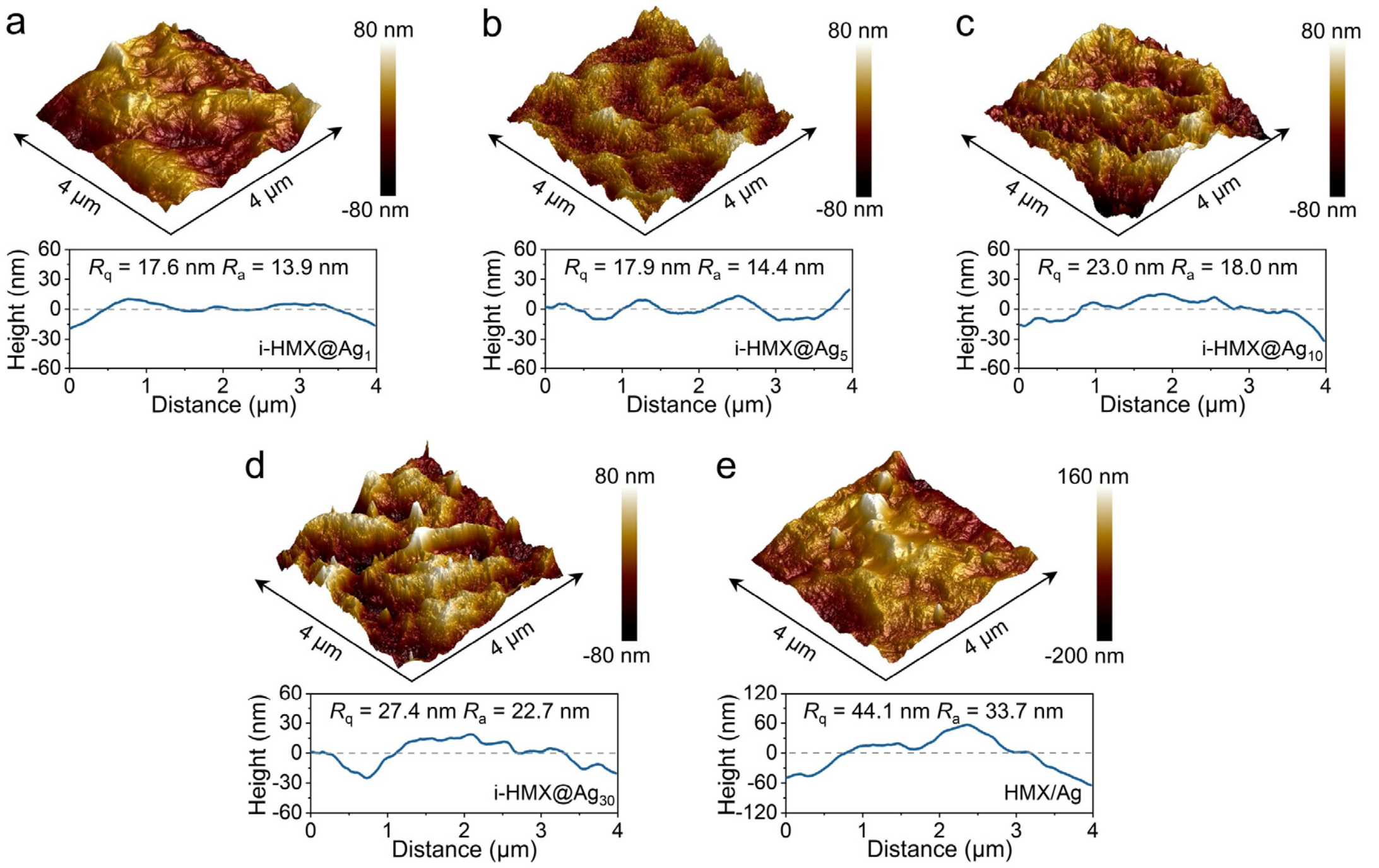

Supplementary Fig. 13. Film morphology and roughness. AFM 3D images and surface roughness of **(a)** i-HMX@Ag_1, **(b)** i-HMX@Ag_5, **(c)** i-HMX@Ag_{10}, **(d)** i-HMX@Ag_{30}, and **(e)** HMX/Ag films.

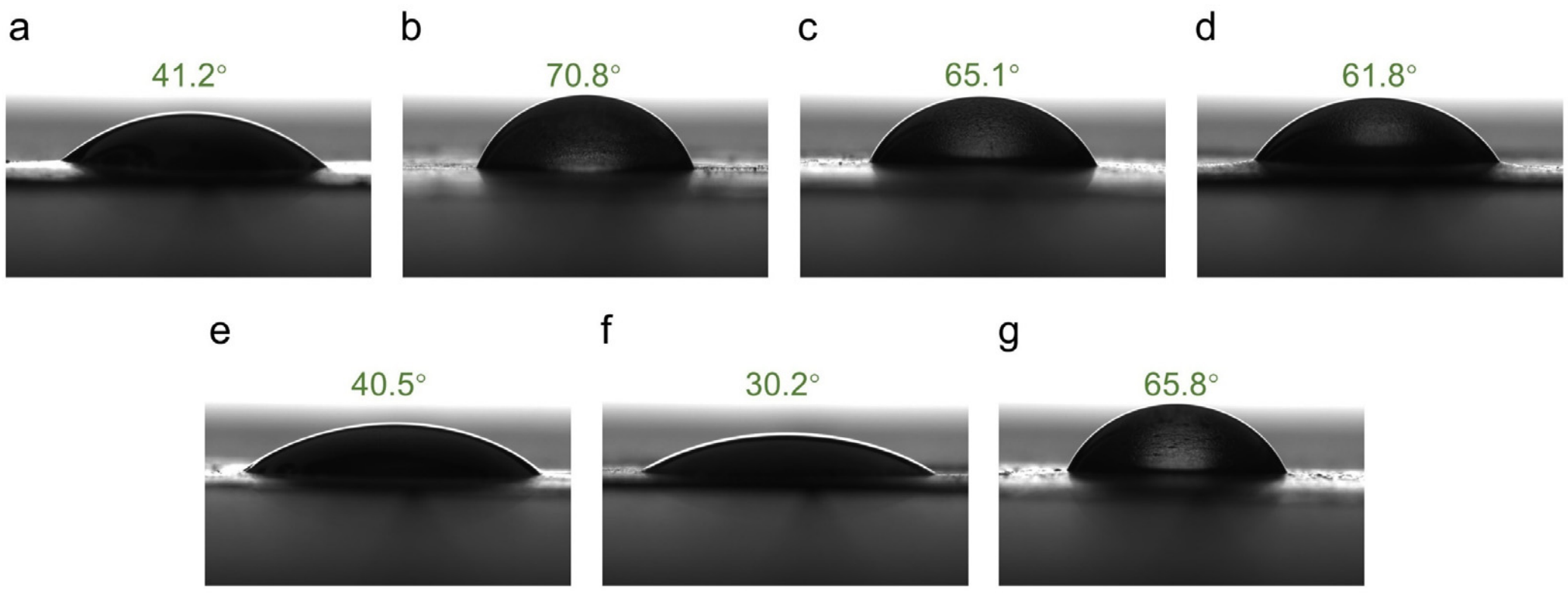

Supplementary Fig. 14. Hydrophilicity analysis. Contact angles of H_2O droplet on **(a)** MXene, **(b)** i-HMX@Ag_1, **(c)** i-HMX@Ag_5, **(d)** i-HMX@Ag_{10}, **(e)** i-HMX@Ag_{20}, **(f)** i-HMX@Ag_{30}, and **(g)** HMX/Ag films.

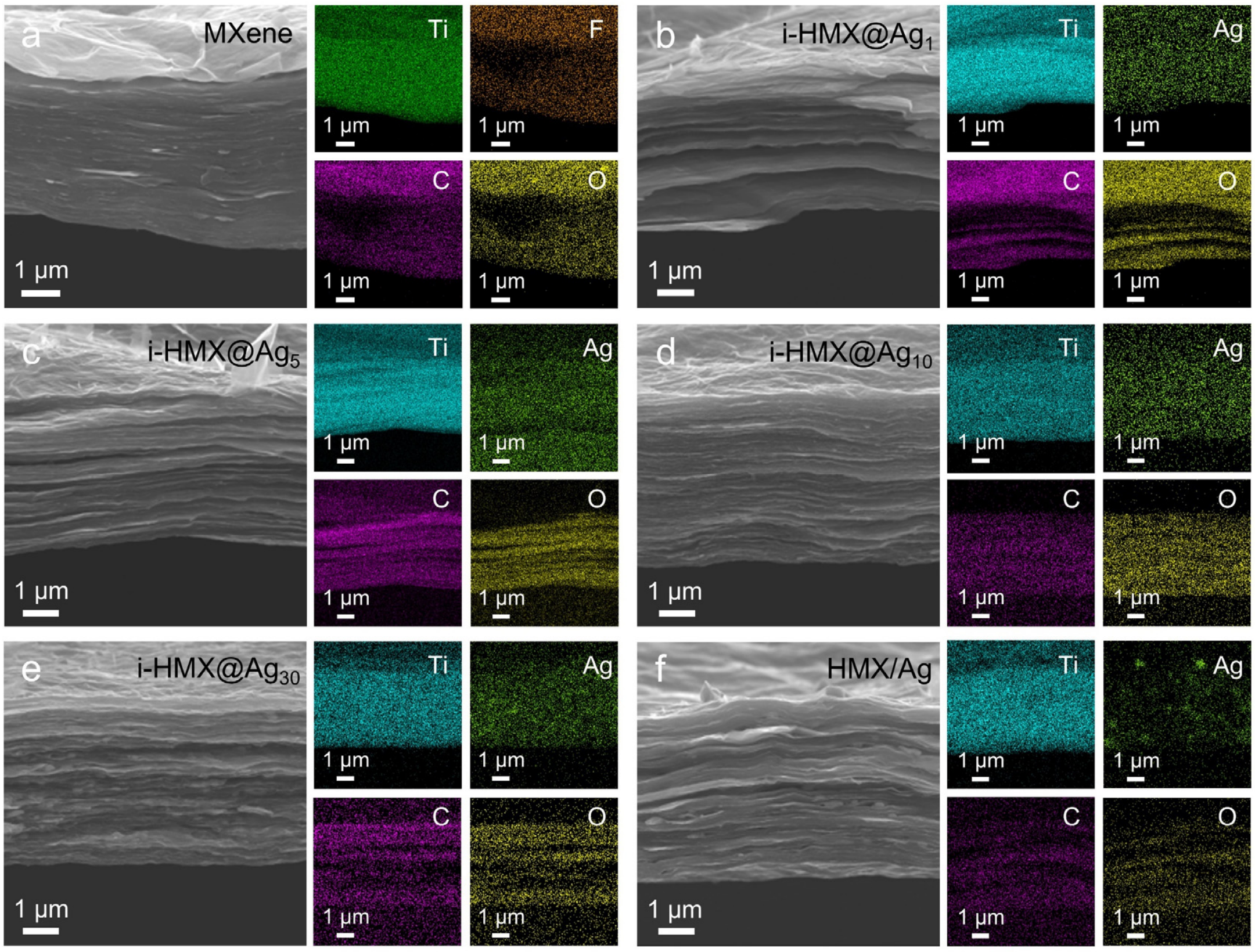

Supplementary Fig. 15. The cross-sectional structure of the films. Cross-sectional SEM images and corresponding EDS elemental mapping images of **(a)** MXene, **(b)** i-HMX@Ag_1, **(c)** i-HMX@Ag_5, **(d)** i-HMX@Ag_{10}, **(e)** i-HMX@Ag_{30}, and **(f)** HMX/Ag films.

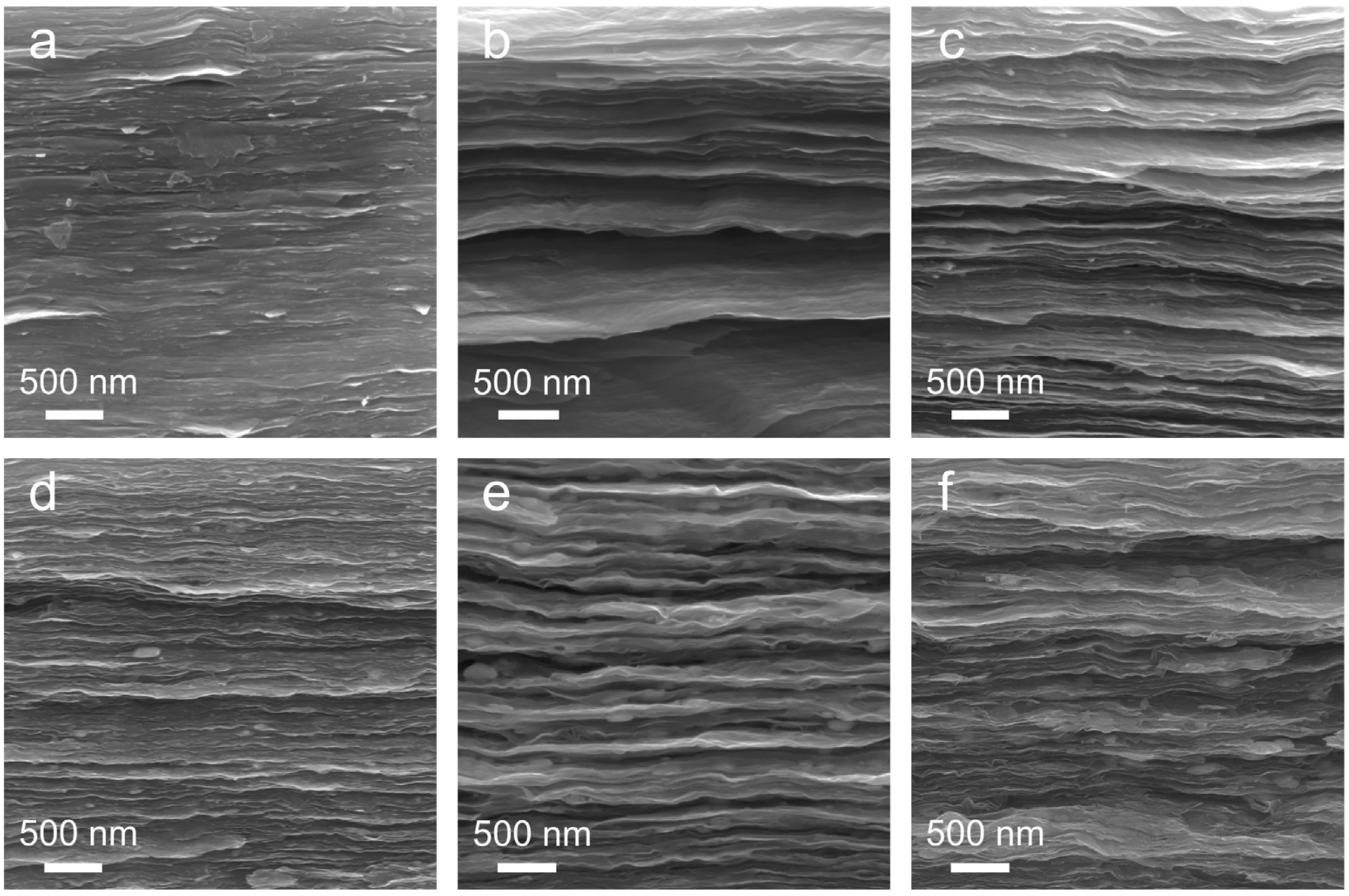

Supplementary Fig. 16. The enlarged cross-sectional structure of the films. Enlarged cross-sectional SEM images of **(a)** MXene, **(b)** i-HMX@Ag_1, **(c)** i-HMX@Ag_5, **(d)** i-HMX@Ag_{10}, **(e)** i-HMX@Ag_{20}, and **(f)** i-HMX@Ag_{30} films.

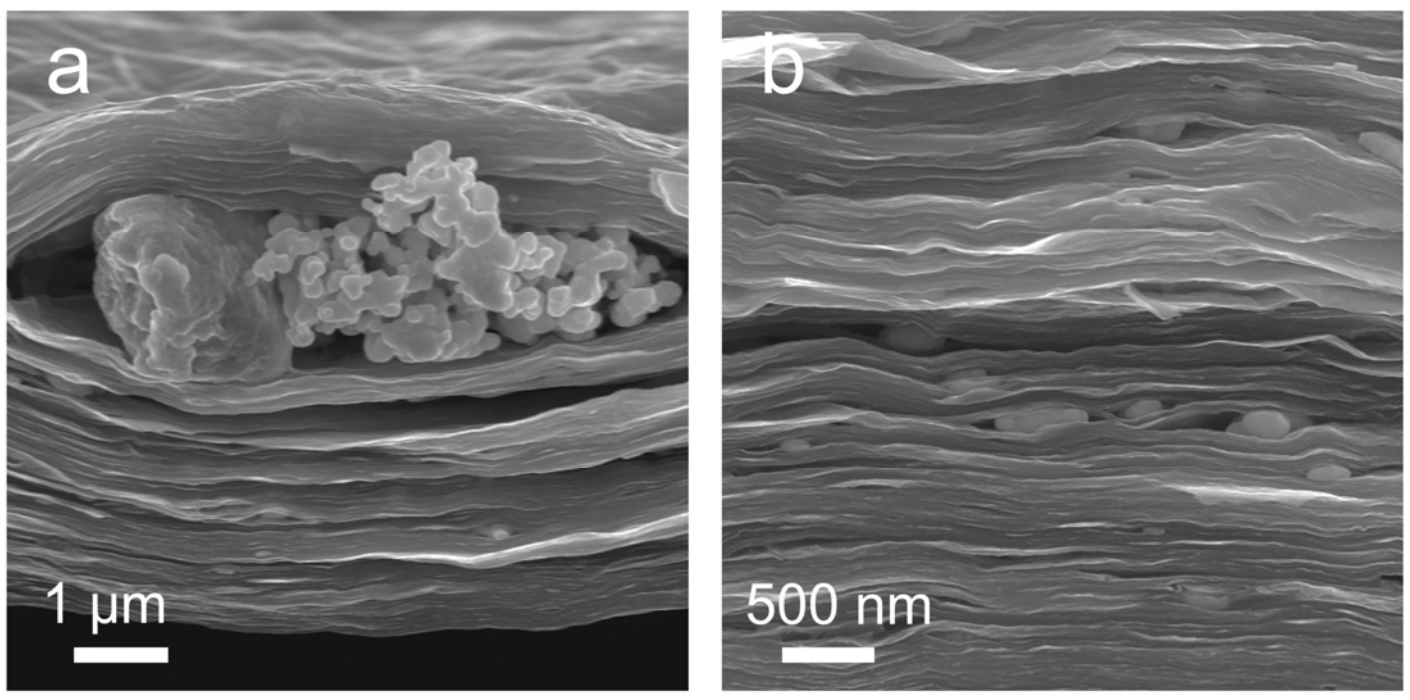

Supplementary Fig. 17. Particle agglomeration phenomenon. Enlarged cross-sectional SEM images of HMX/Ag film.

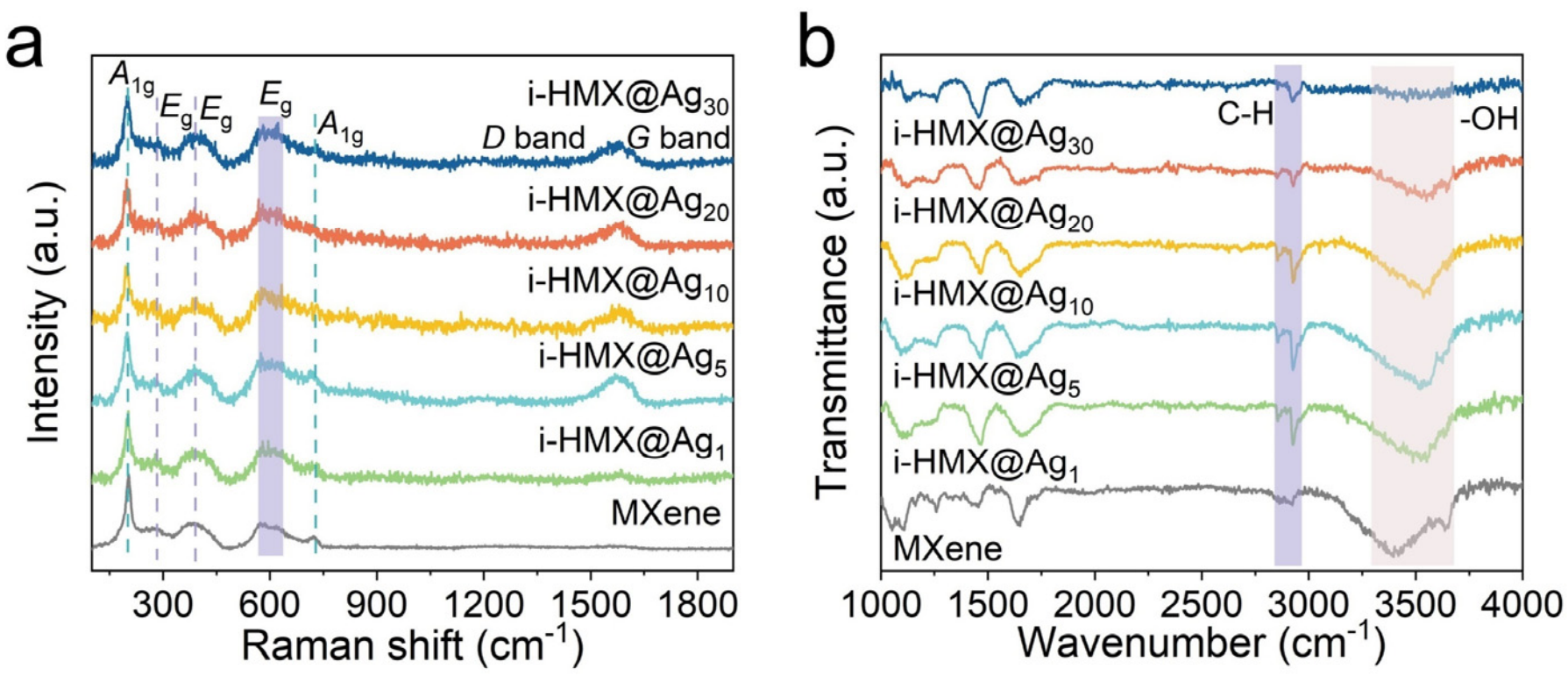

Supplementary Fig. 18. Characterization analysis of i-HMX@Ag$_x$. (a) Raman spectra and **(b)** FT-IR spectra of MXene, i-HMX@Ag$_1$, i-HMX@Ag$_5$, i-HMX@Ag$_{10}$, i-HMX@Ag$_{20}$, and i-HMX@Ag$_{30}$ films.

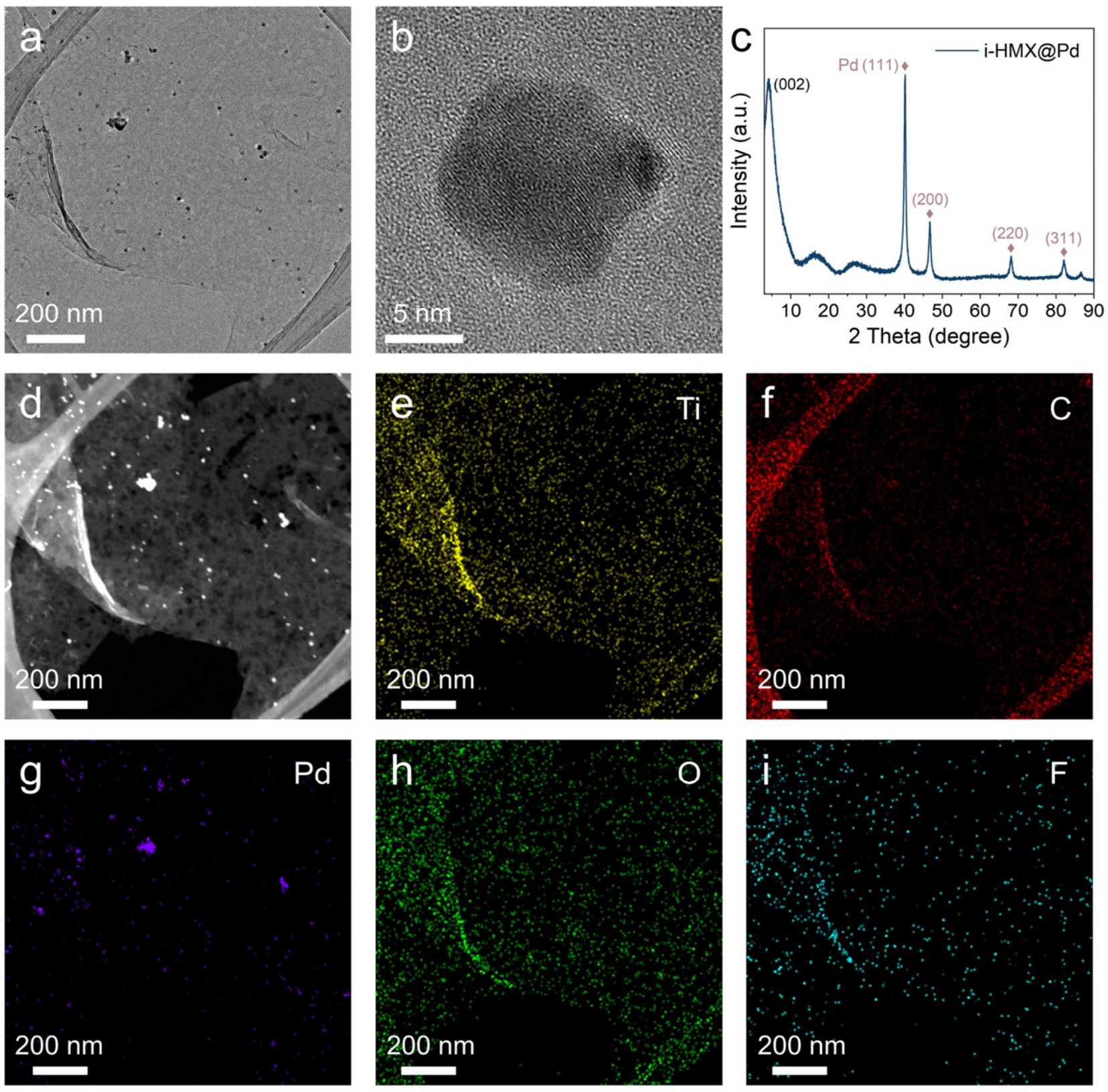

Supplementary Fig. 19. Verification of the applicability of the in situ etching-and-reduction strategy. (a) TEM image, **(b)** HRTEM image, **(c)** XRD pattern, **(d-i)** HAADF-STEM and corresponding EDS elemental mapping images of i-HMX@Pd.

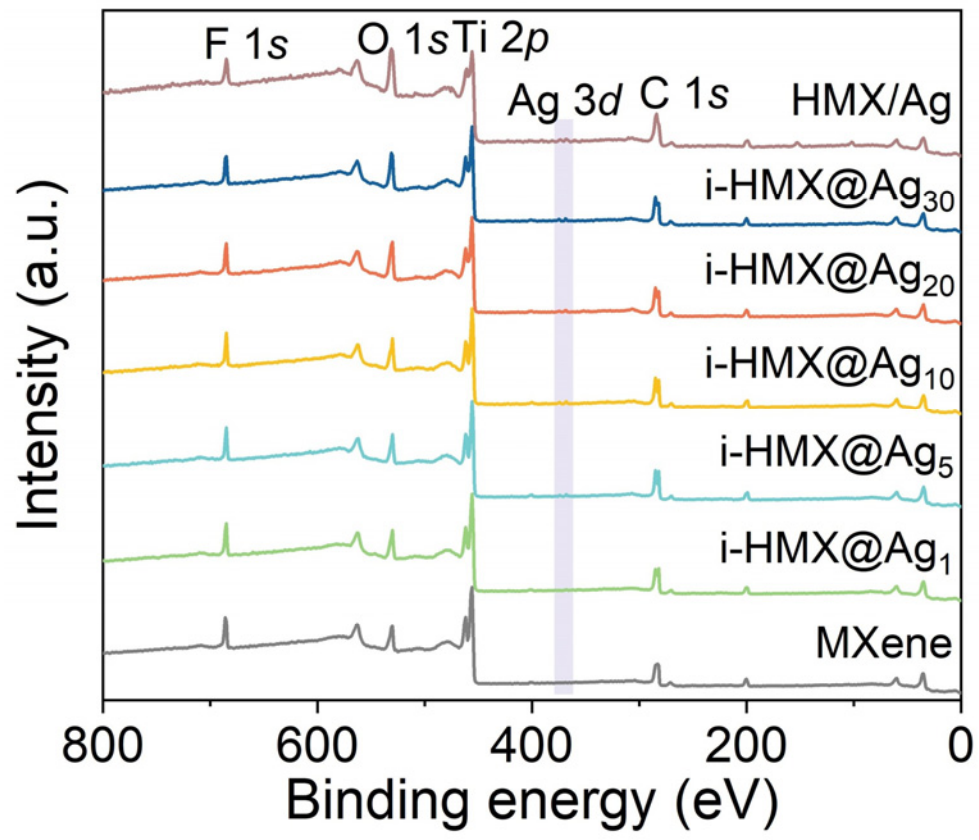

Supplementary Fig. 20. Element analysis. XPS survey spectra of MXene, i-HMX@Ag_1, i-HMX@Ag_5, i-HMX@Ag_{10}, i-HMX@Ag_{20}, i-HMX@Ag_{30}, and HMX/Ag films.

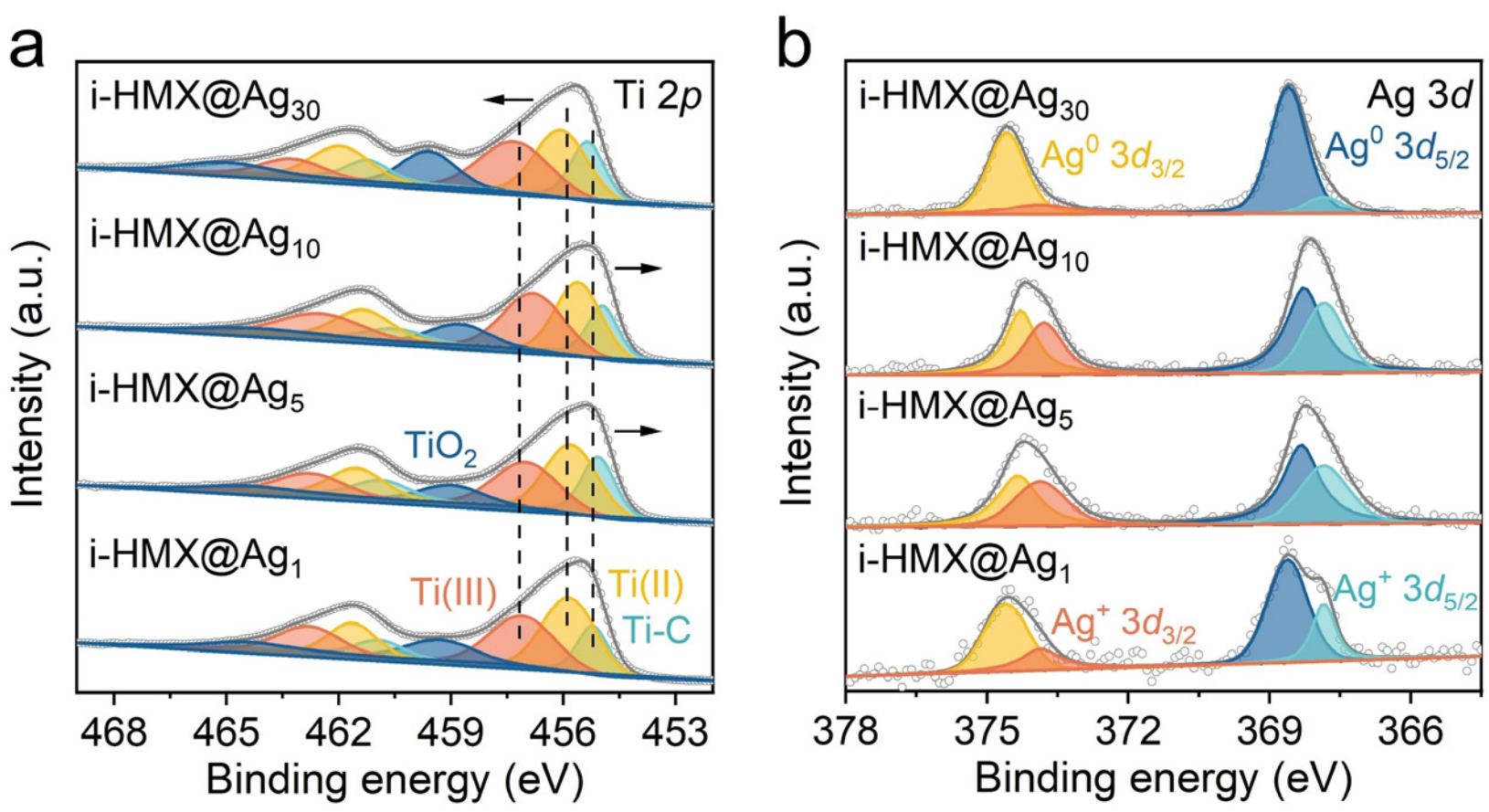

Supplementary Fig. 21. Chemical state analysis of Ti and Ag. High-resolution XPS spectra of **(a)** Ti 2*p* and **(b)** Ag 3*d* for i-HMX@Ag_1, i-HMX@Ag_5, i-HMX@Ag_{10}, and i-HMX@Ag_{30} films.

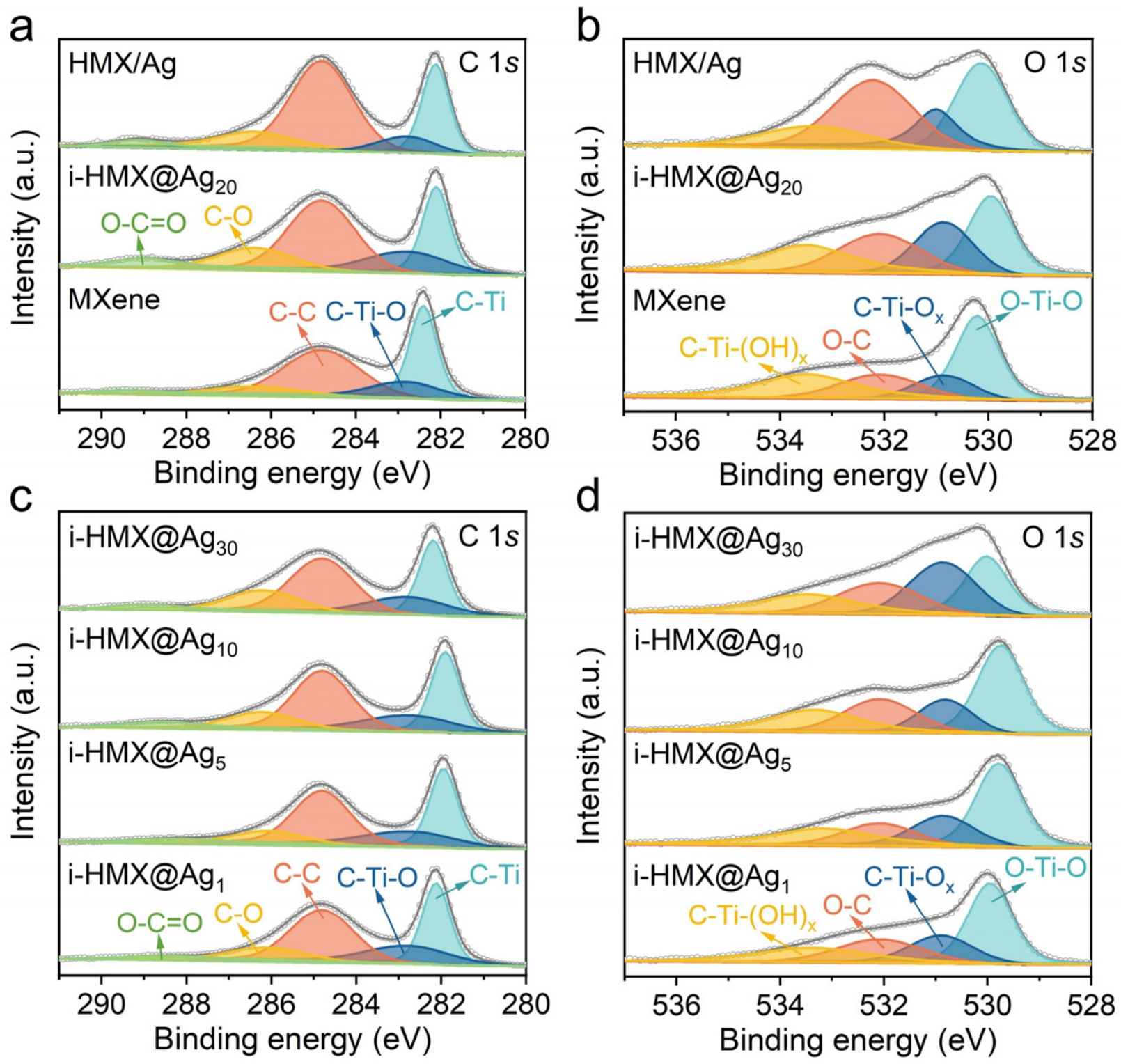

Supplementary Fig. 22. Chemical state analysis of C and O. High-resolution XPS spectra of **(a, c)** C 1*s* and **(b, d)** O 1*s* for MXene, i-HMX@Ag_1, i-HMX@Ag_5, i-HMX@Ag_{10}, i-HMX@Ag_{20}, i-HMX@Ag_{30}, and HMX/Ag films.

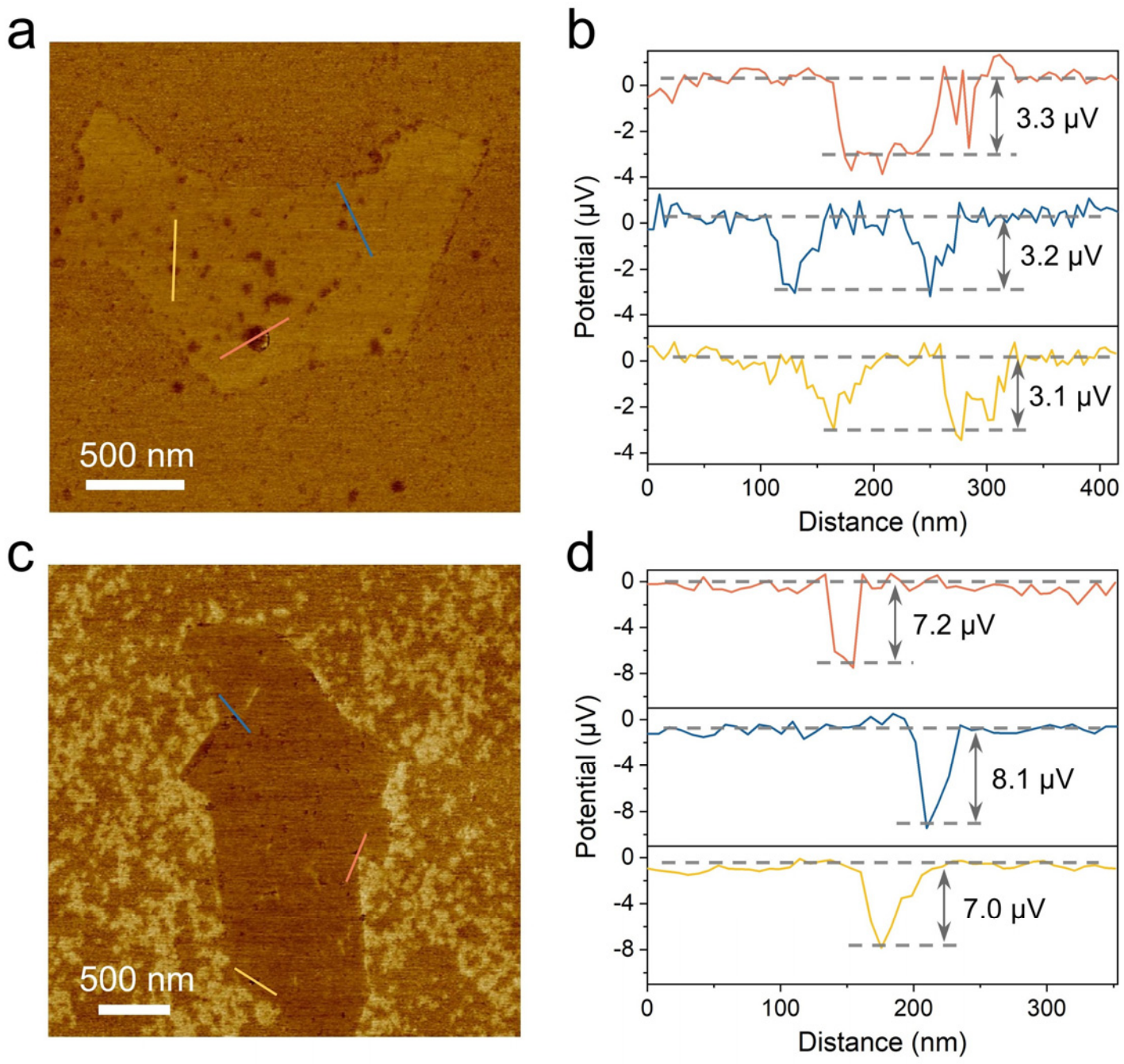

Supplementary Fig. 23. Surface potentials changes. Kelvin probe force microscopy (KPFM) images and corresponding surface potentials of **(a, b)** i-HMX@Ag_{20} and **(c, d)** HMX/Ag. A probe tip of SCM-PIT-V2 was used for KPFM. The specimens were mounted on a conductive silicon wafer by dropping a drop of the diluted dispersion solution (0.01 mg mL^{-1}) and drying.

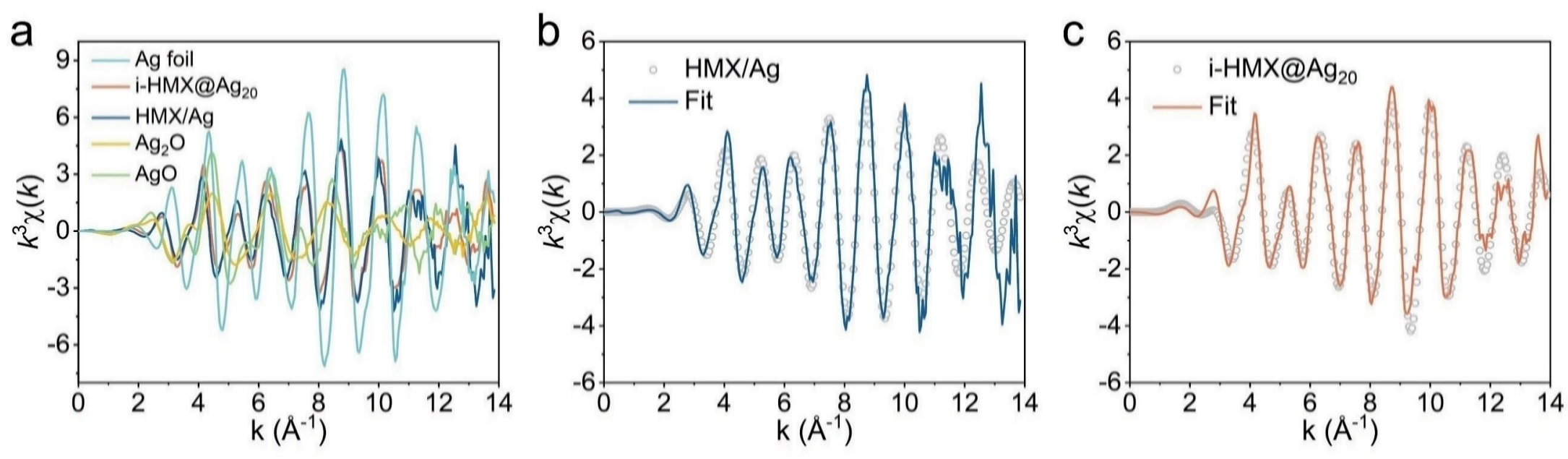

Supplementary Fig. 24. EXAFS analysis in K space. (a) EXAFS spectra in K space of Ag foil, Ag_2O, AgO, HMX/Ag, and i-HMX@Ag_{20}. EXAFS fitting results in K space of **(b)** HMX/Ag and **(c)** i-HMX@Ag_{20}.

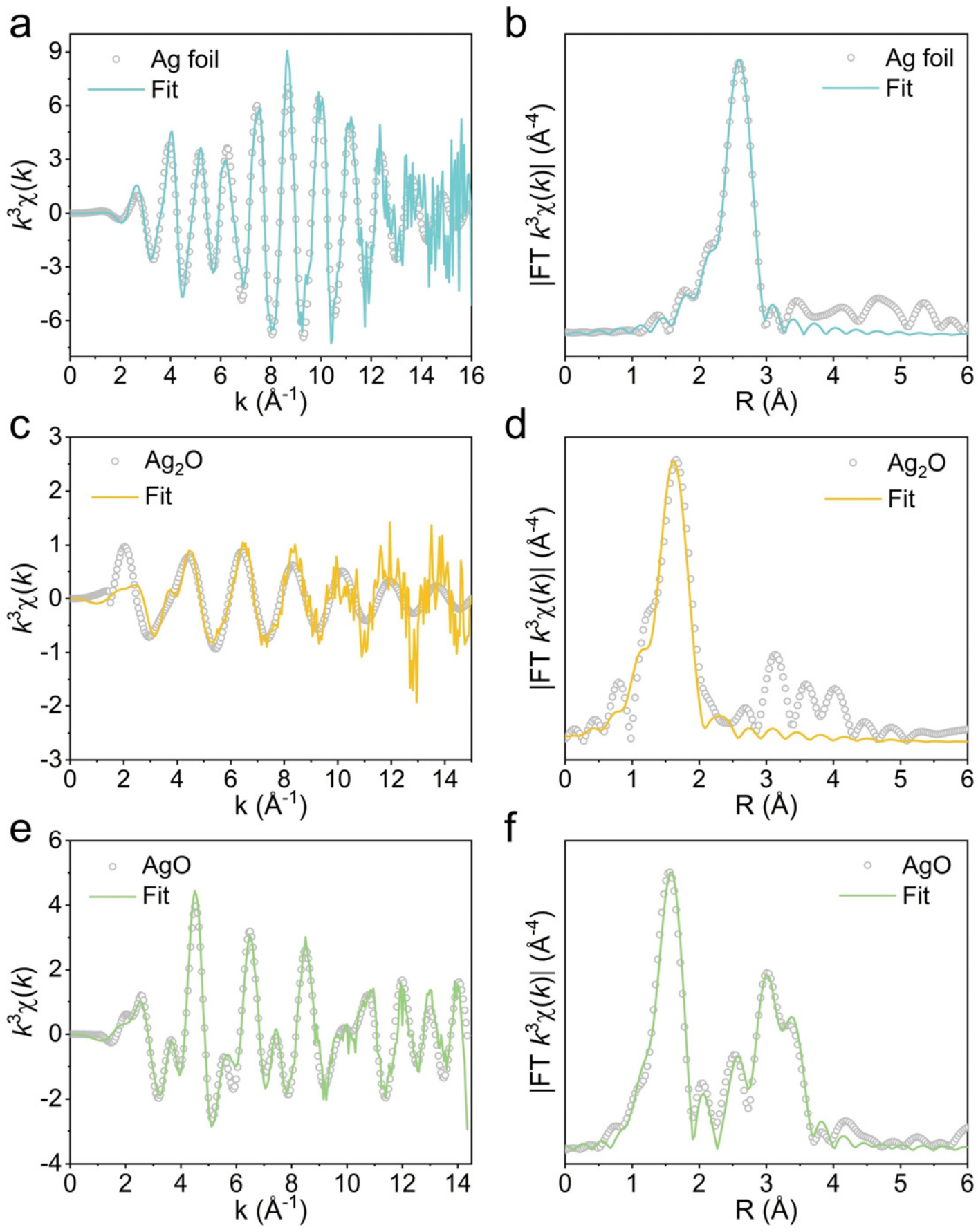

Supplementary Fig. 25. EXAFS analysis in R space. EXAFS fitting results in **(a, c, e)** K space and **(b, d, f)** R space of Ag foil, Ag_2O, and AgO.

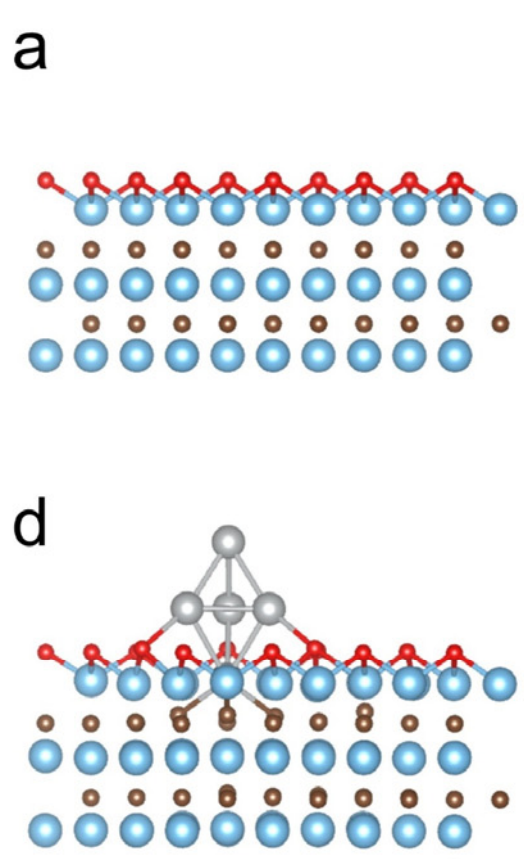

Supplementary Fig. 26. Atomic configurations optimization. The side view of the optimized atomic configurations for **(a)** $Ti_3C_2O_2$, **(b)** $Ti_3C_2O_2$/Ag, **(c)** $Ti_3C_2O_2$@Ag with three OVs, **(d, e)** $Ti_3C_2O_2$@Ag with four OVs, and **(f)** $Ti_3C_2O_2$@Ag with five OVs.

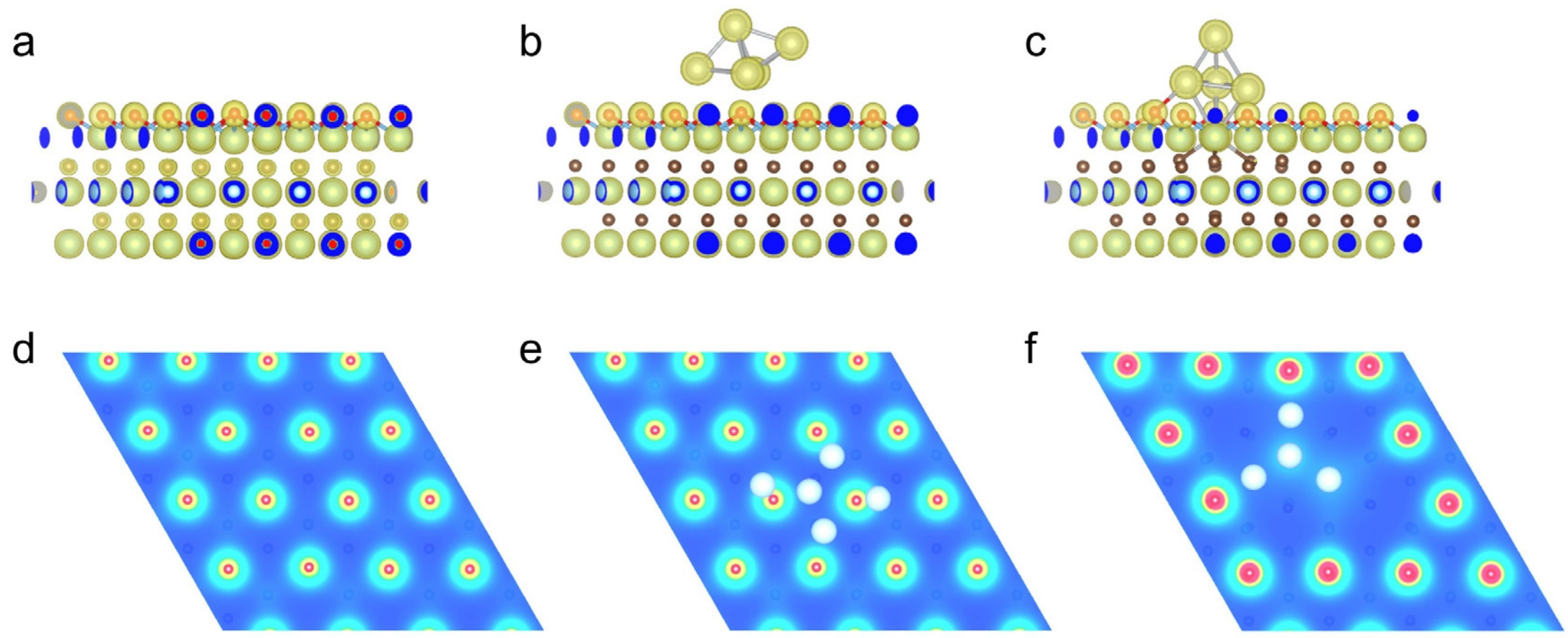

Supplementary Fig. 27. Electron density distribution analysis. The electron density distribution diagrams in **(a, d)** $Ti_3C_2O_2$, **(b, e)** $Ti_3C_2O_2$/Ag, and **(c, f)** the optimal $Ti_3C_2O_2$@Ag.

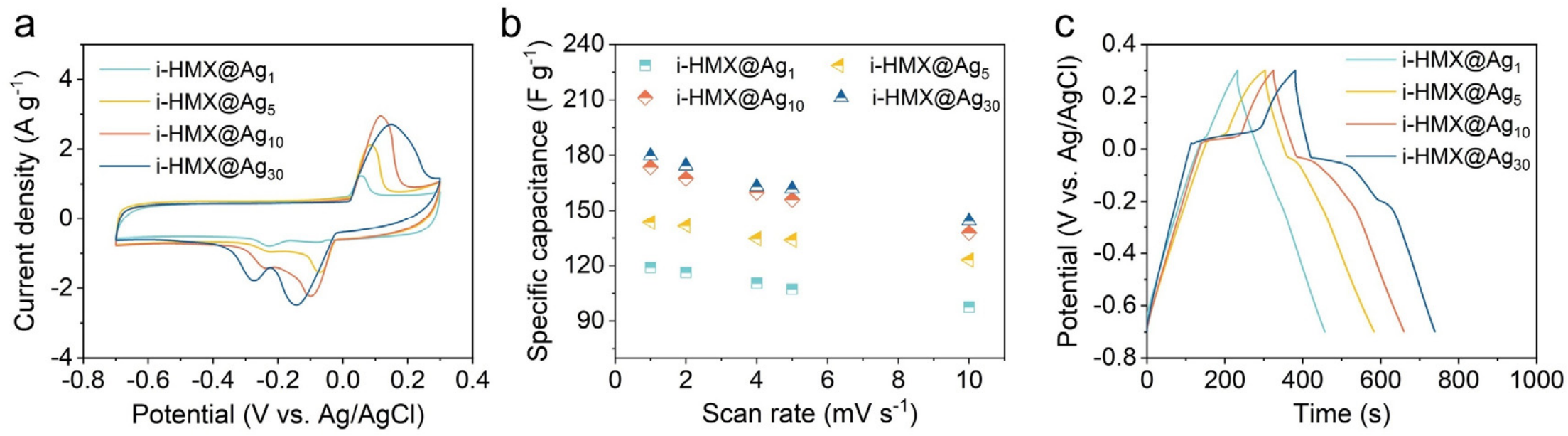

Supplementary Fig. 28. Electrochemical performance of i-HMX@Ag*x*. (a) CV curves of i-HMX@Ag1, i-HMX@Ag5, i-HMX@Ag10, and i-HMX@Ag30 at 5 mV s^{-1}. **(b)** Specific capacitance of i-HMX@Ag1, i-HMX@Ag5, i-HMX@Ag10, and i-HMX@Ag30 at various scan rates. **(c)** GCD profiles of i-HMX@Ag1, i-HMX@Ag5, i-HMX@Ag10, and i-HMX@Ag30 at 0.5 A g^{-1}.

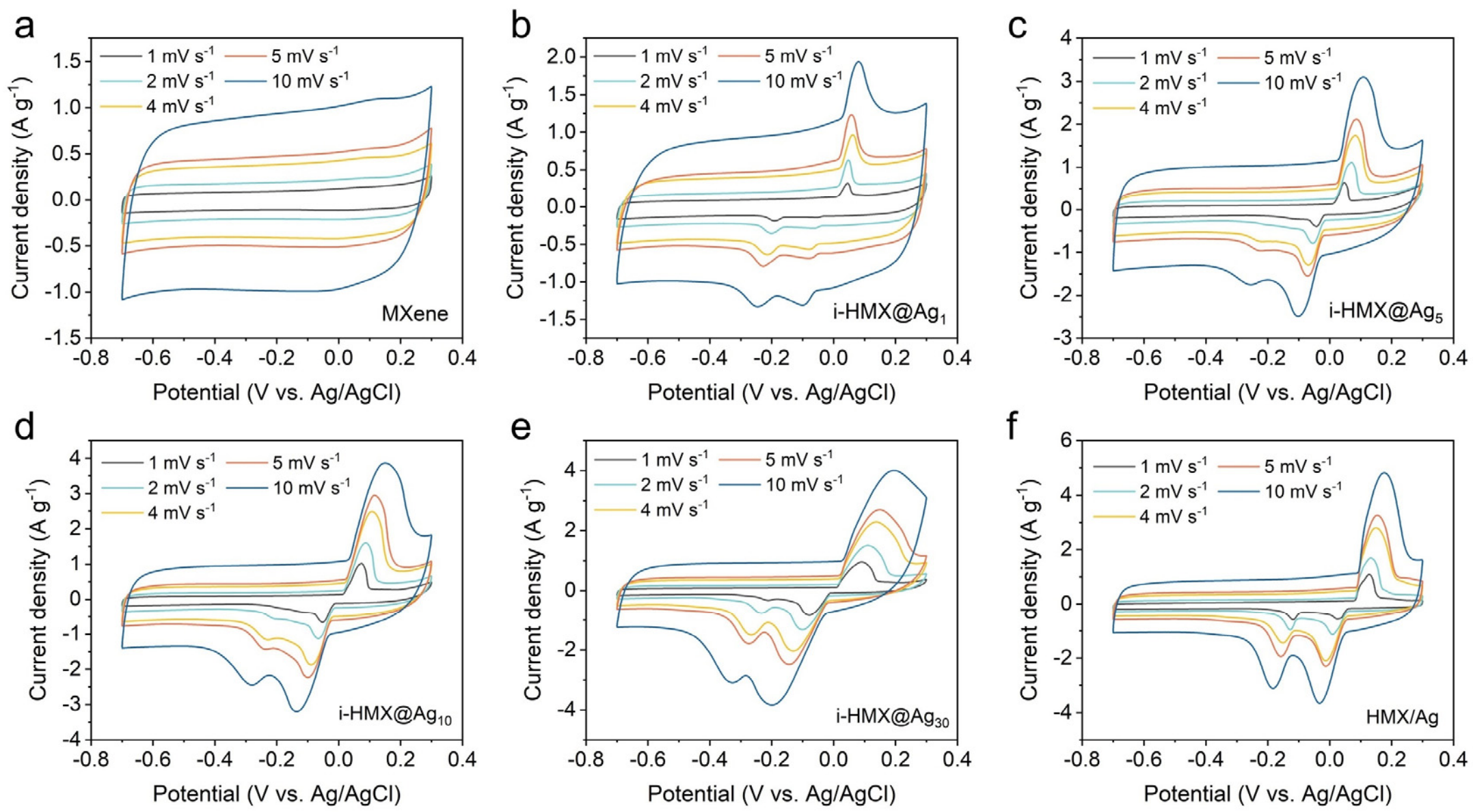

Supplementary Fig. 29. CV diagrams under different operating conditions. CV curves of **(a)** MXene, **(b)** i-HMX@Ag1, **(c)** i-HMX@Ag5, **(d)** i-HMX@Ag10, **(e)** i-HMX@Ag30, and **(f)** HMX/Ag at different scan rates.

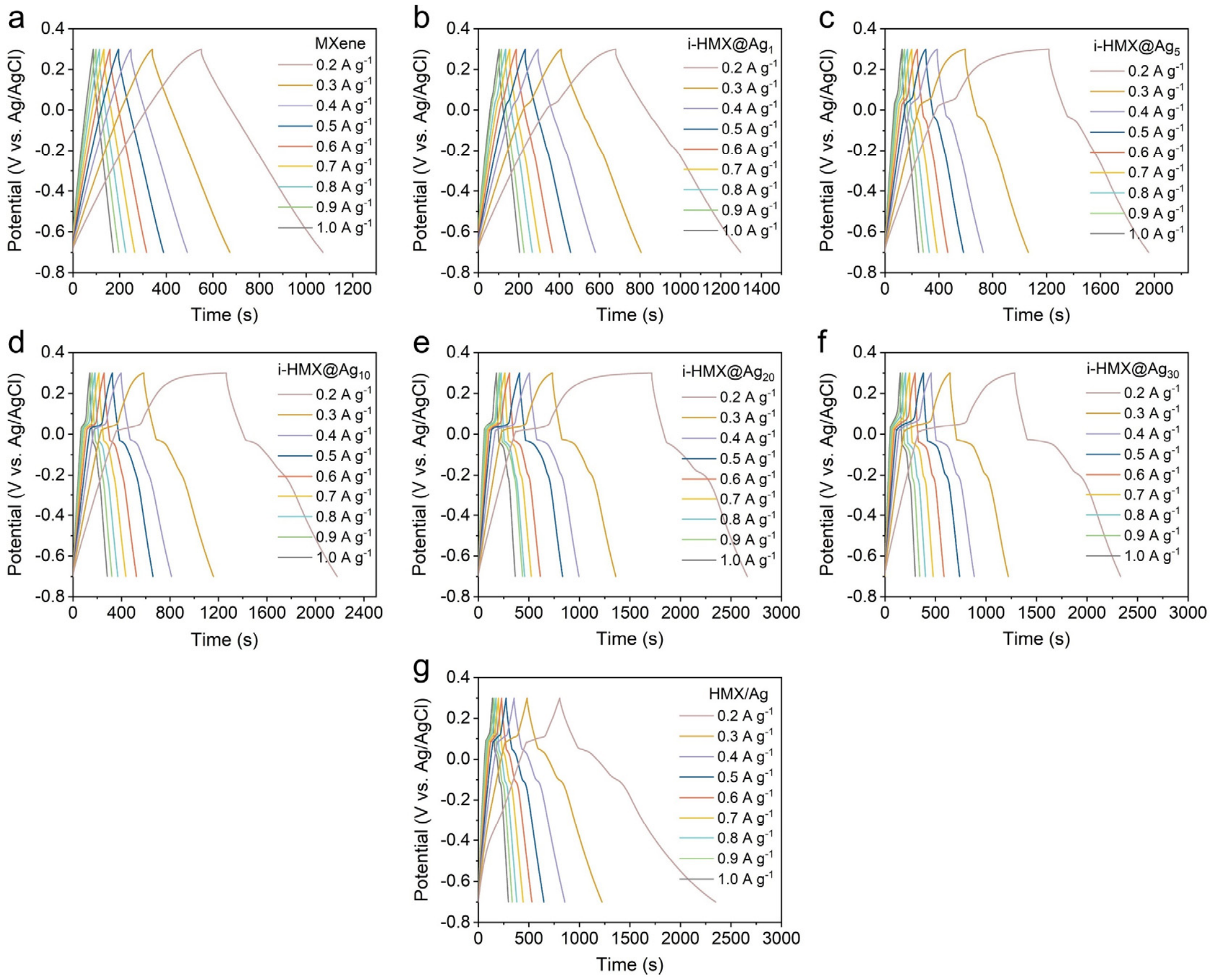

Supplementary Fig. 30. GCD diagrams under different operating conditions. GCD profiles of **(a)** MXene, **(b)** i-HMX@Ag_1, **(c)** i-HMX@Ag_5, **(d)** i-HMX@Ag_{10}, **(e)** i-HMX@Ag_{20}, **(f)** i-HMX@Ag_{30}, and **(g)** HMX/Ag at different current densities.

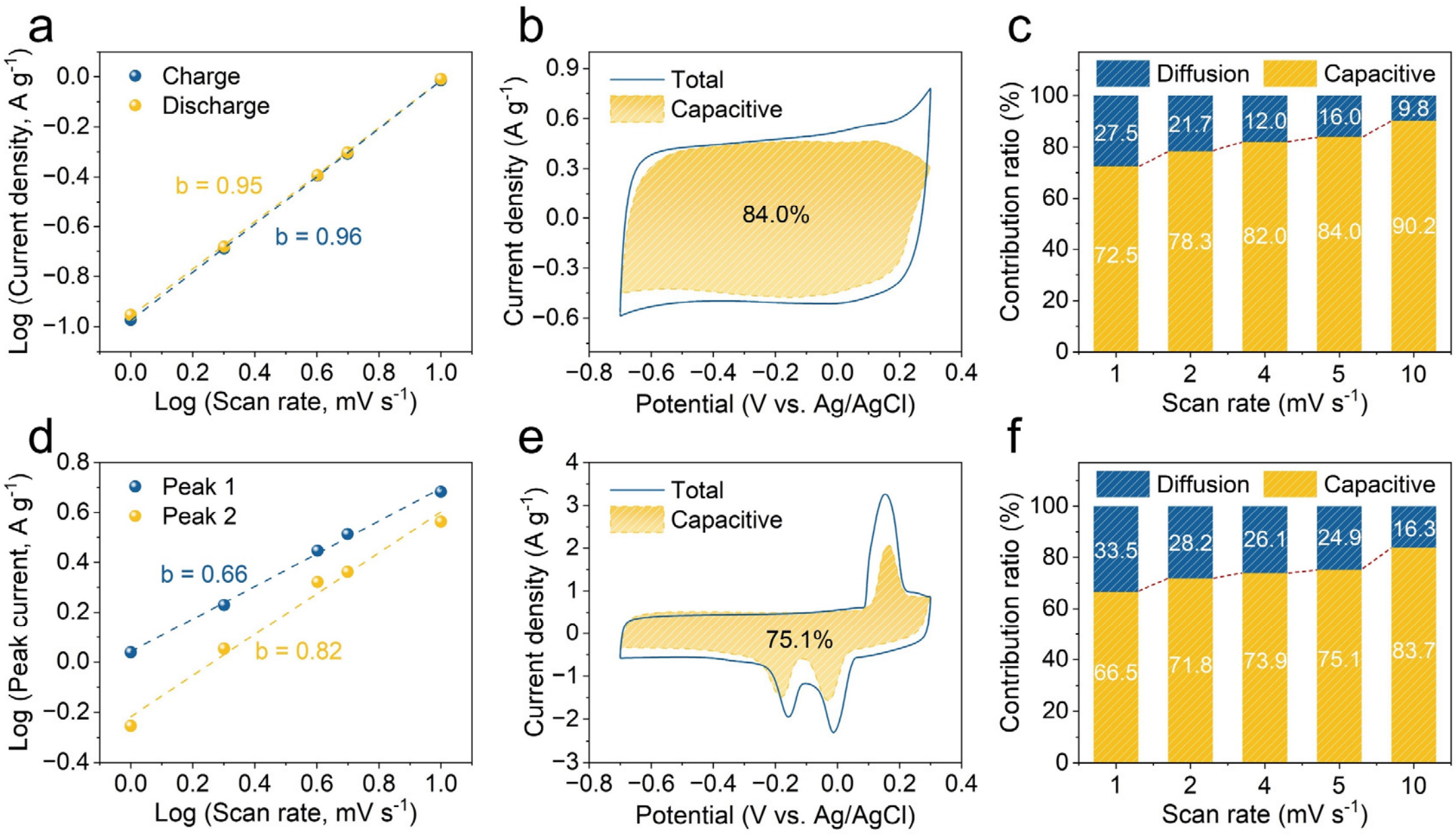

Supplementary Fig. 31. Electrochemical kinetics of MXene and HMX/Ag film. Determination of b-value, the capacitive contribution at 5 mV s^{-1}, and the contribution ratios of capacitive and diffusion at various scan rates of **(a-c)** MXene film and **(d-f)** HMX/Ag film. In **(a)** and **(d)**, the plots were conducted by linear fitting; R^2 are 0.9999 (charge) and 0.9997 (discharge) in **(a)**; R^2 are 0.9968 (peak 1) and 0.9815 (peak 2) in **(d)**.

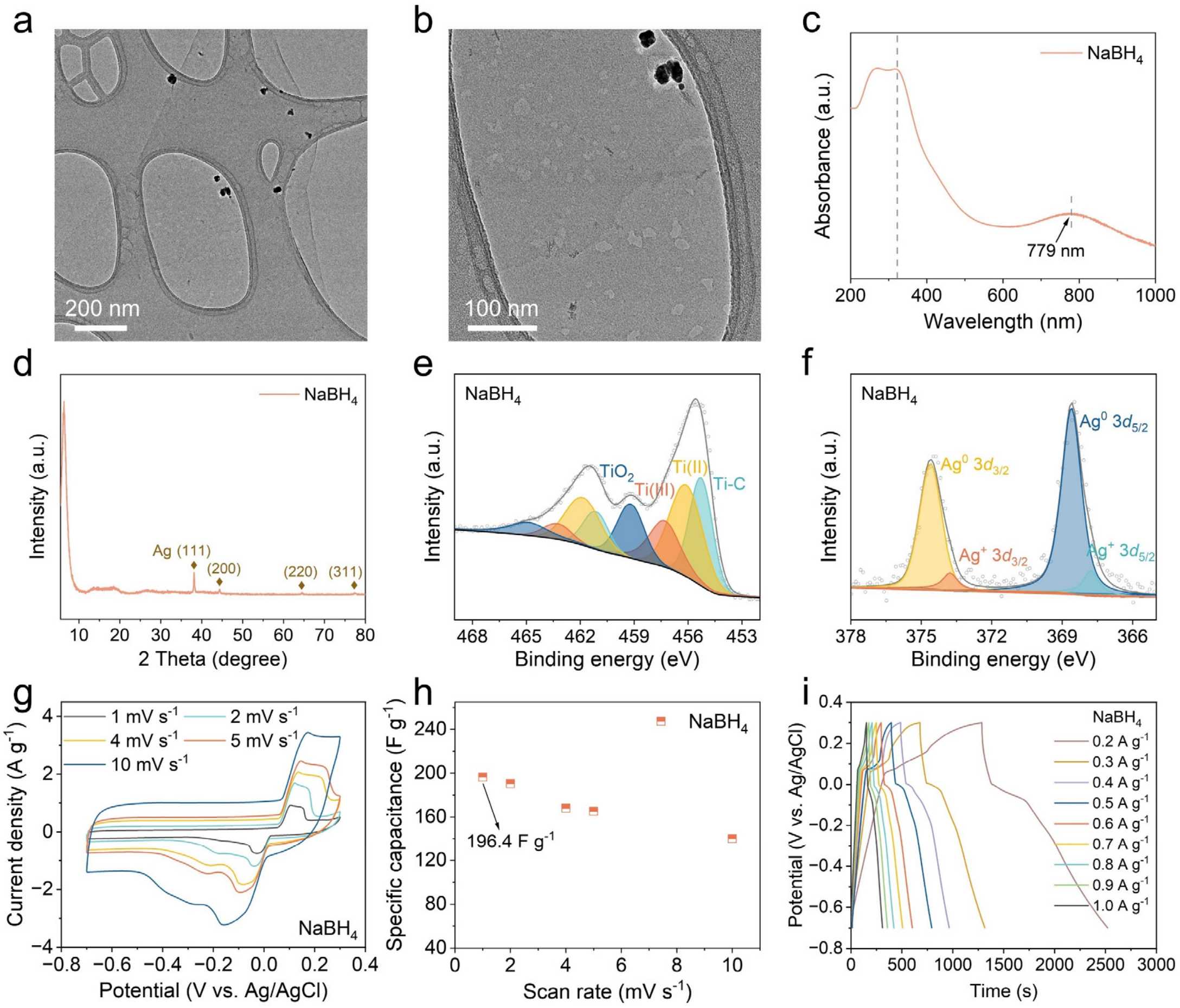

Supplementary Fig. 32. Characterization and performance of the $NaBH_4$ assisted sample. (a, b) TEM images, **(c)** UV-Vis spectrum, **(d)** XRD pattern, **(e)** Ti 2*p*, and **(f)** Ag 3*d* XPS spectra of Ag−MXene prepared with $NaBH_4$. **(g)** CV curves and **(h)** corresponding specific capacitances at different scan rates. **(i)** GCD profiles at different current densities.

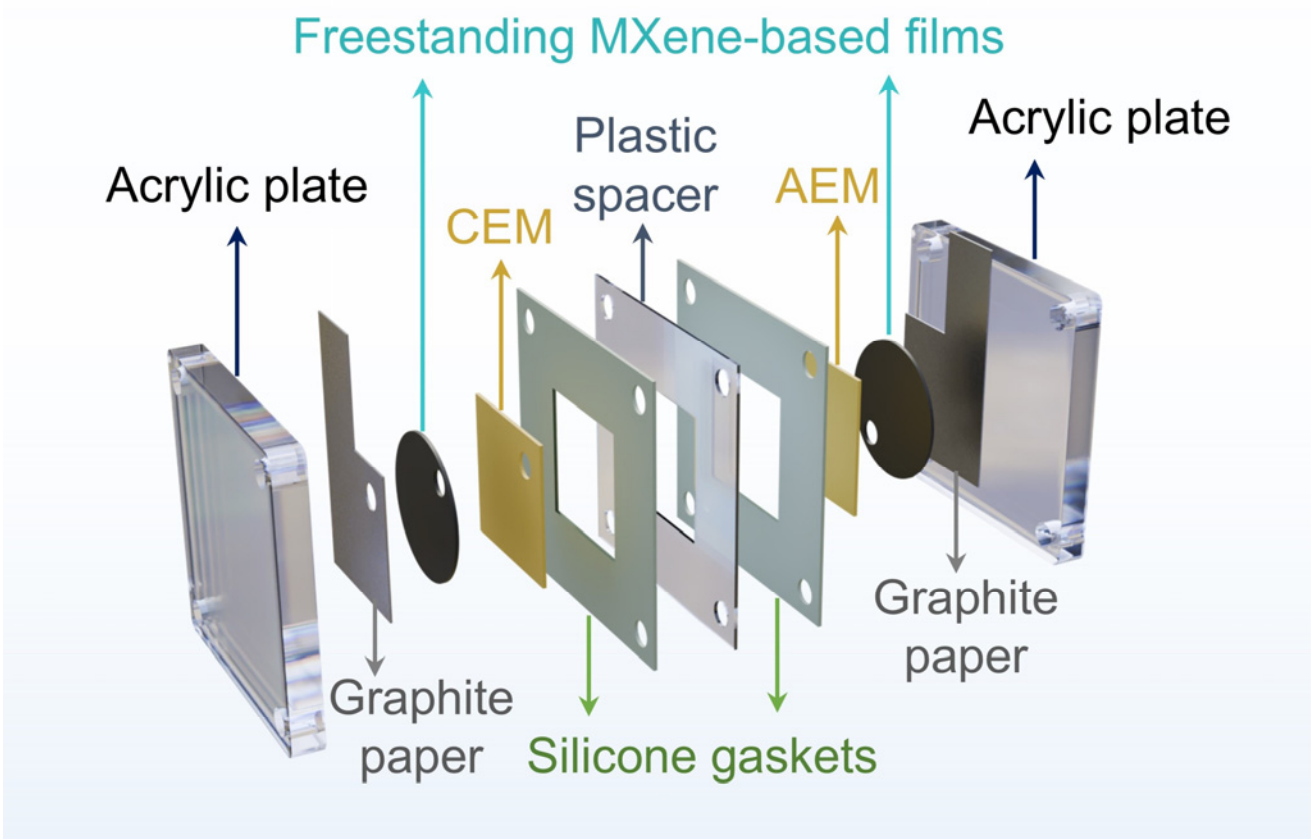

Supplementary Fig. 33. Dechlorination cell. Schematic diagram of the CDI dechlorination device.

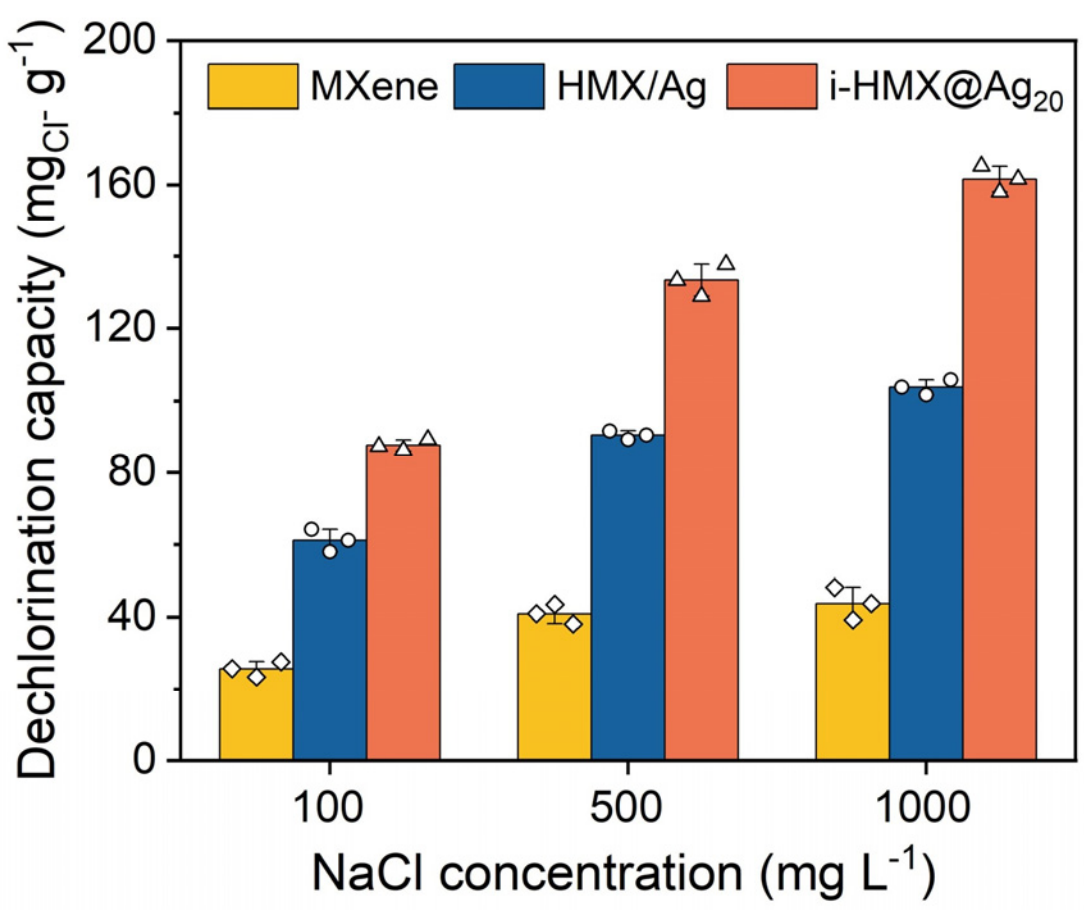

Supplementary Fig. 34. The effect of salt concentration on capacity. Dechlorination capacity of MXene, HMX/Ag, and i-HMX@Ag_{20} electrodes in a NaCl solution with different concentrations at 1.2 V. Data are presented as mean ± standard deviation; error bars represent standard deviation; n = 3 independent systems.

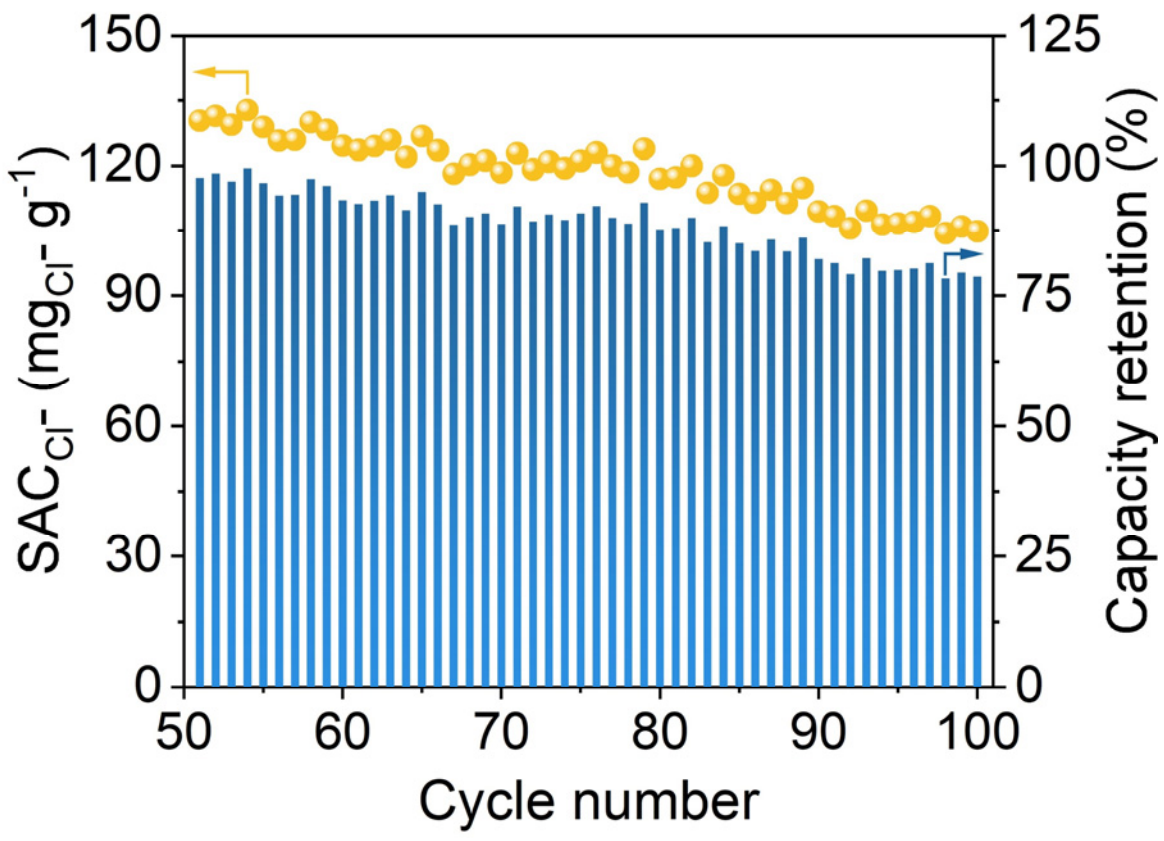

Supplementary Fig. 35. Long-term stability. Cyclic stability of i-HMX@Ag_{20} electrode over 51 to 100 cycles. Capacity retention is defined as the ratio of the dechlorination capacity at the nth cycle to that of the first cycle, expressed as a percentage. The capacity retention is 78.6% after 100 cycles.

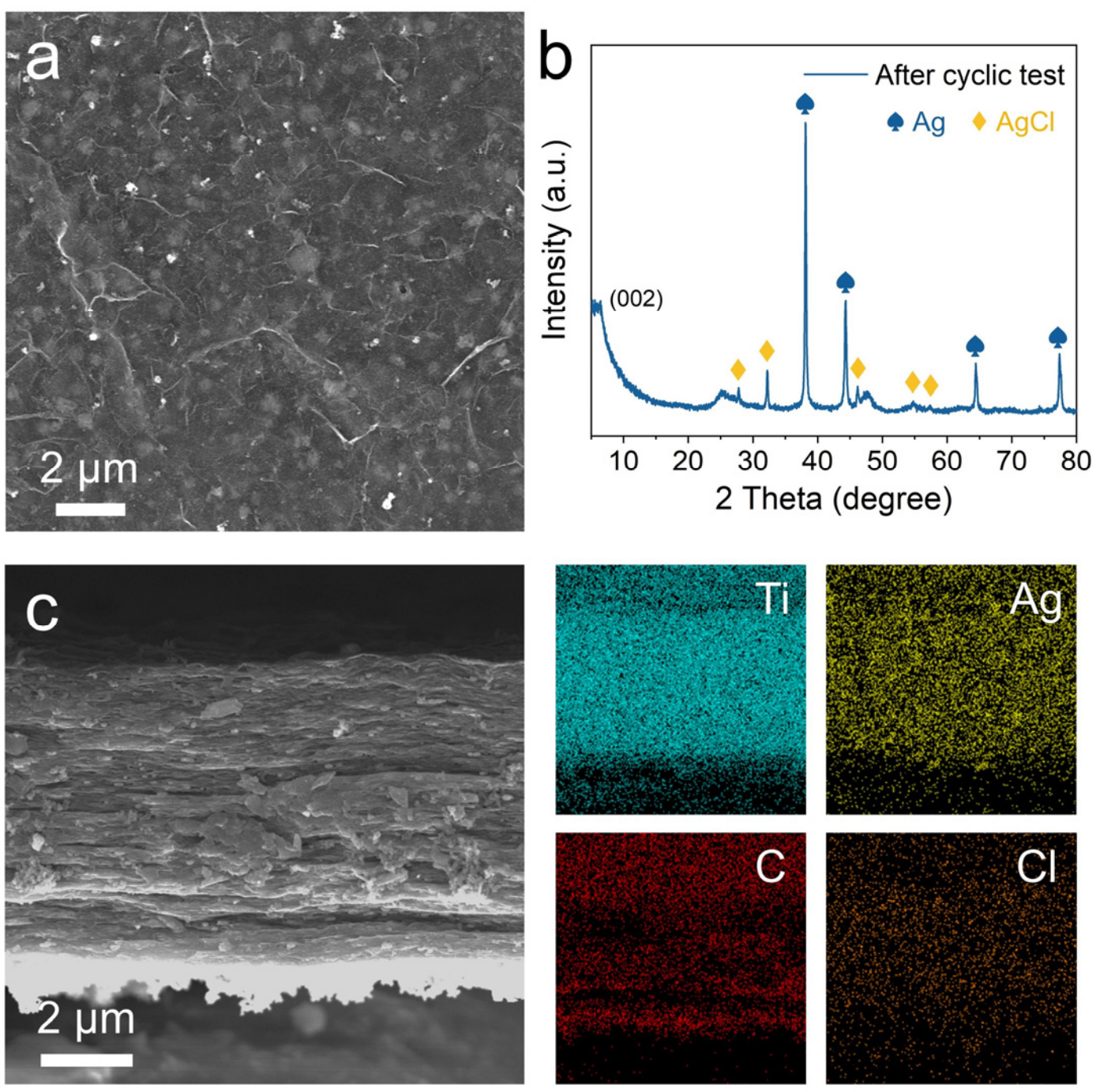

Supplementary Fig. 36. Characterization analysis after cycling. (a) Surface SEM image, **(b)** XRD pattern, and **(c)** cross-sectional SEM image and corresponding EDS elemental mapping of i-HMX@Ag_{20} film after cyclic test.

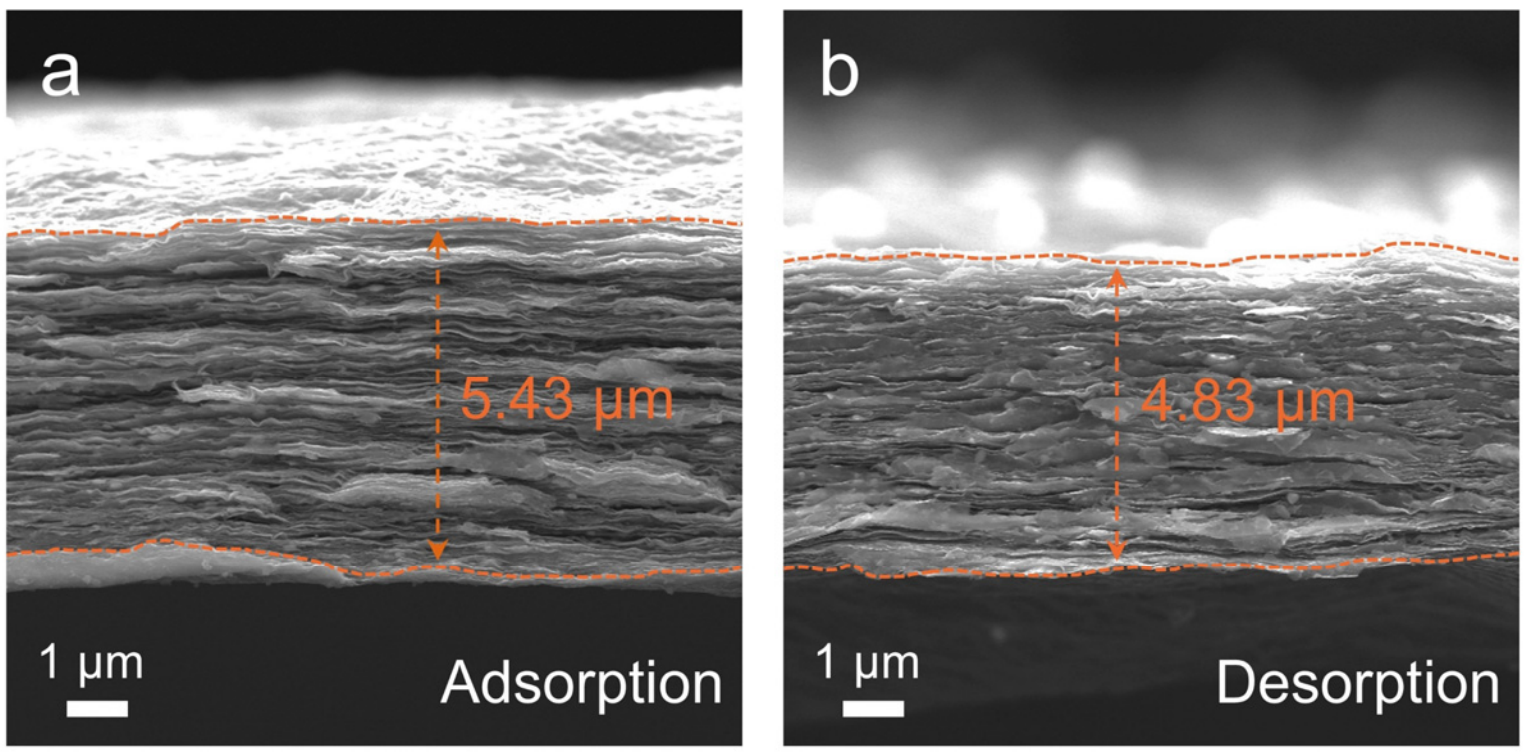

Supplementary Fig. 37. Volume expansion analysis. Cross-sectional SEM images of i-HMX@Ag_{20} film after **(a)** adsorption and **(b)** desorption.

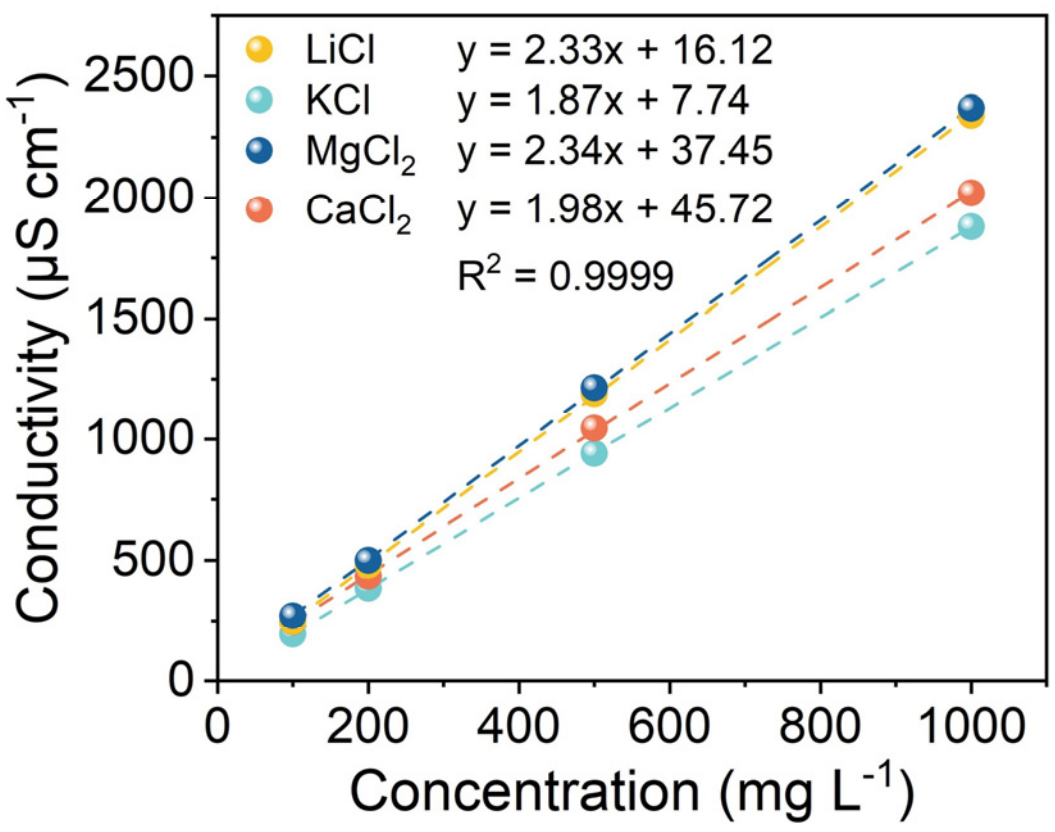

Supplementary Fig. 38. Standard curves of different salt solutions. Linear fitting relationship between salt concentration and conductivity in LiCl, KCl, $MgCl_2$, and $CaCl_2$ solution, respectively. The plots were conducted by linear fitting; $R^2 = 0.9999$.

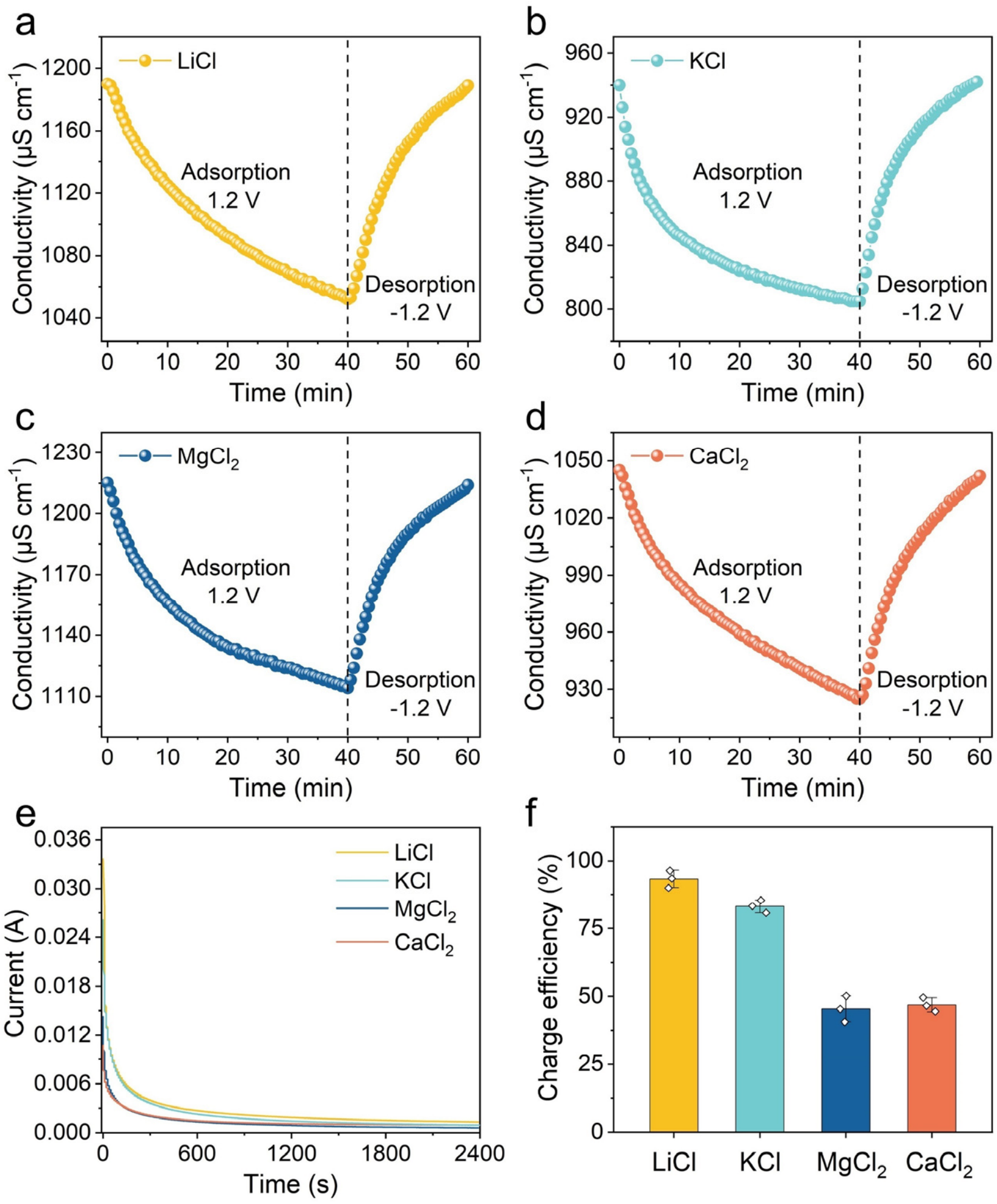

Supplementary Fig. 39. Dechlorination performance in different salt solutions. Conductivity versus time curves of the i-HMX@Ag_{20} electrode during adsorption and desorption processes in 500 mg L^{-1} **(a)** LiCl solution, **(b)** KCl solution, **(c)** $MgCl_2$ solution, and **(d)** $CaCl_2$ solution. **(e)** Current plots and **(f)** the corresponding charge efficiencies in different dechlorination systems. Data in **(f)** are presented as mean ± standard deviation; error bars represent standard deviation; n = 3 independent systems.

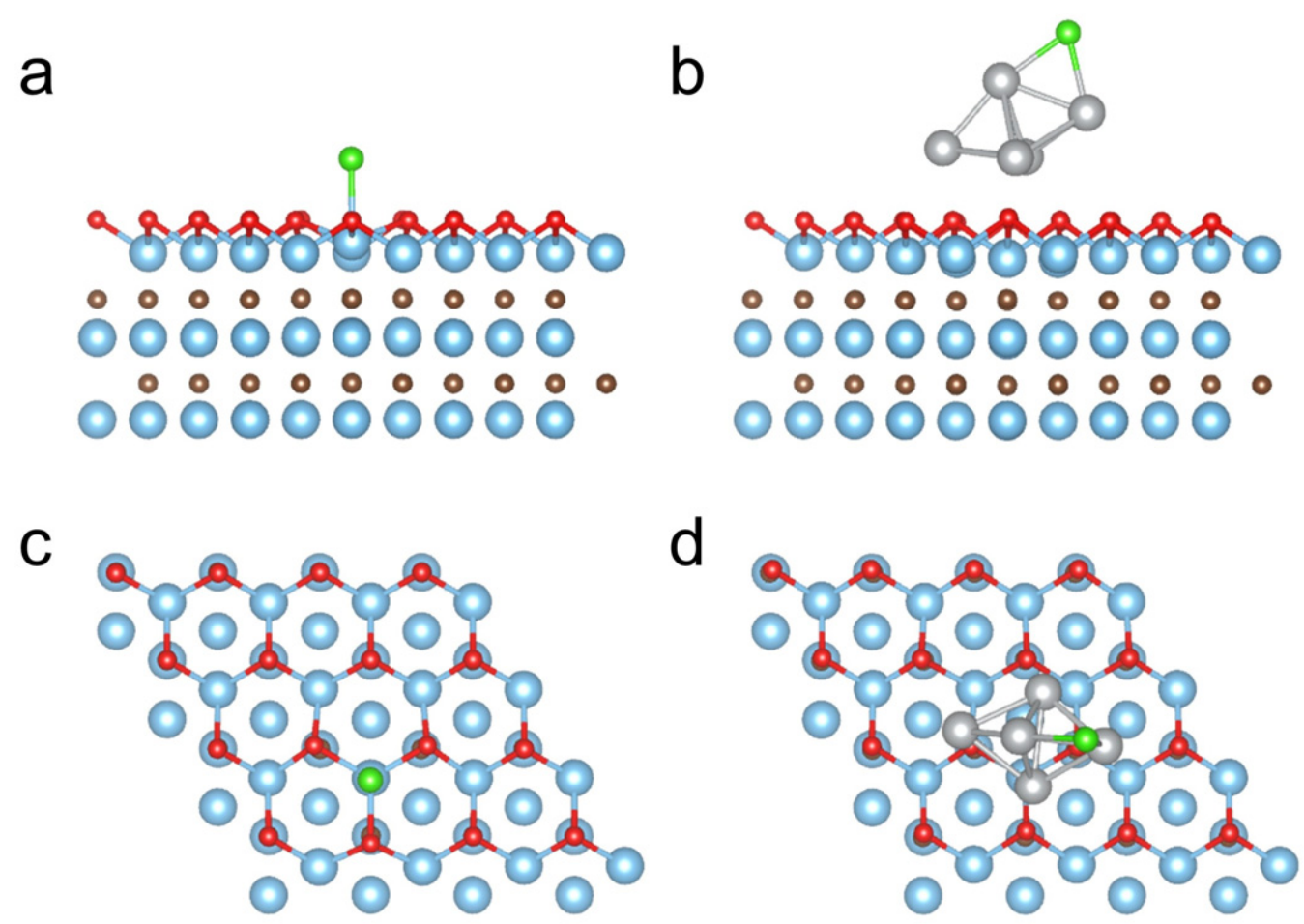

Supplementary Fig. 40. Adsorption configuration of control samples. (a) The side view and **(c)** top view of adsorption configuration for a Cl atom on $Ti_3C_2O_2$. **(b)** The side view and **(d)** top view of adsorption configuration for a Cl atom on $Ti_3C_2O_2$/Ag.

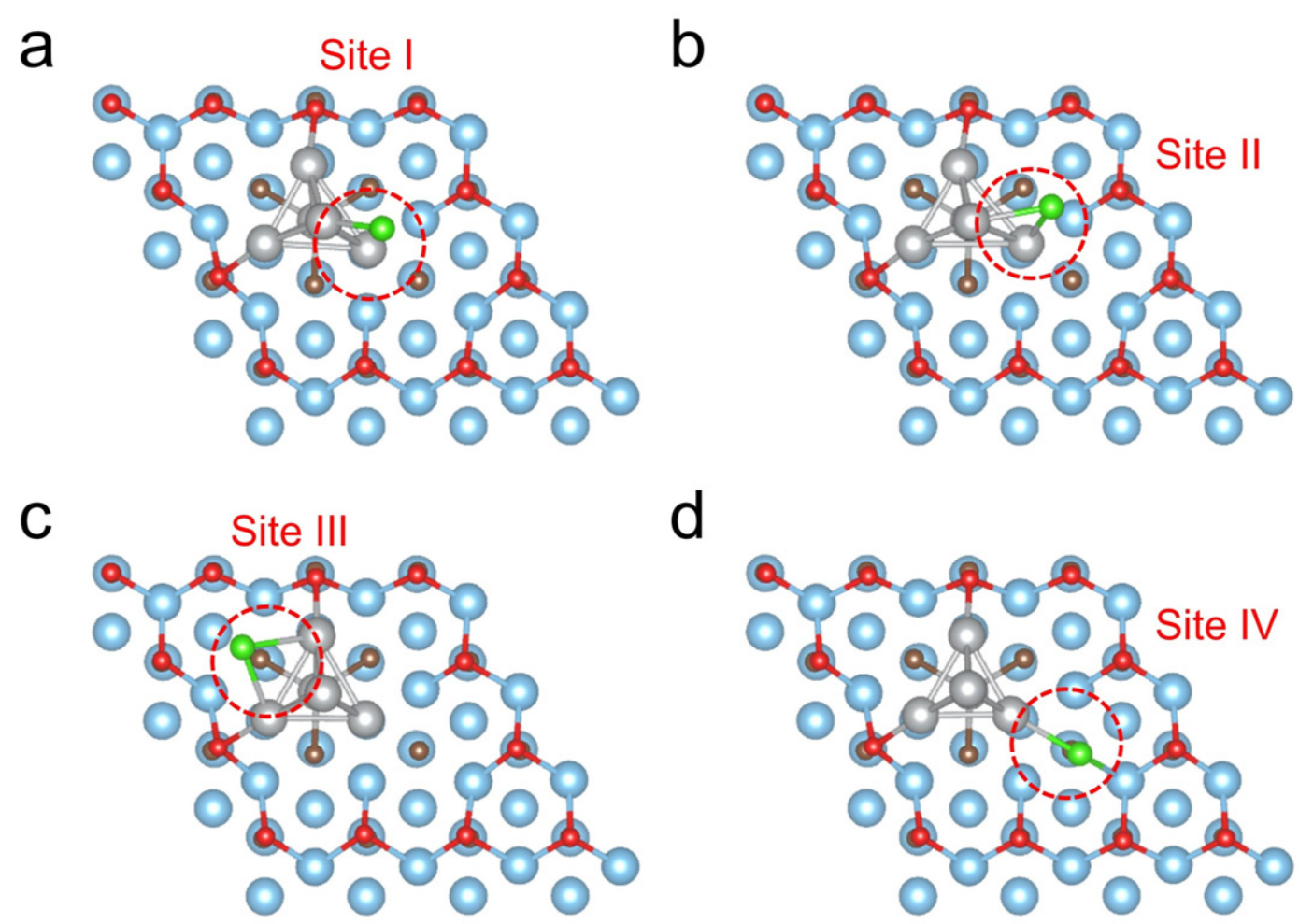

Supplementary Fig. 41. Adsorption configuration of $Ti_3C_2O_2$@Ag. The top view of adsorption configurations for a Cl atom on $Ti_3C_2O_2$@Ag with different active sites.

Supplementary Table 1. Theoretical and measured Ag content in different samples.

Samples	Theoretical Ag content (wt.%)	Ag content by ICP-OES (wt.%)
i-HMX@Ag_1	0.64	0.22
i-HMX@Ag_5	3.18	1.35
i-HMX@Ag_{10}	6.35	3.69
i-HMX@Ag_{20}	12.70	8.48
i-HMX@Ag_{30}	19.05	13.32

Supplementary Table 2. EXAFS fitting parameters at the Ag *K*-edge for various samples.

Sample	Shell	CN[a]	R (Å)[b]	σ^2 (Å^2)[c]	ΔE_0 (eV)[d]	R factor
Ag foil	Ag-Ag	12.0	2.86 ± 0.01	0.0095	-6.6 ± 0.2	0.0023
AgO	Ag-O1	2.1 ± 0.2	2.03 ± 0.01	0.0044	4.4 ± 1.0	0.0039
	Ag-Ag1	0.3 ± 0.1	3.27 ± 0.01	0.0030		
	Ag-O2	4.5 ± 0.9	3.43 ± 0.01	0.0109		
	Ag-Ag2	1.4 ± 0.5	3.74 ± 0.01	0.0030		
Ag_2O	Ag-O1	1.0 ± 0.1	1.99 ± 0.01	0.0019	8.3 ± 2.5	0.0152
	Ag-O2	4.0 ± 0.1	2.72 ± 0.01	0.0032		
i-HMX@Ag_{20}	Ag-O/C	1.1 ± 0.2	2.04 ± 0.01	0.0049	-2.5 ± 0.5	0.0054
	Ag-Ag	5.0 ± 0.4	2.86 ± 0.01	0.0087		
HMX/Ag	Ag-Ag	6.4 ± 0.6	2.86 ± 0.01	0.0096	-4.0 ± 0.6	0.0117

[a]CN, coordination numbers; [b]R, the distance between absorber and backscatter atoms; [c]σ^2, Debye-Waller factors; [d]ΔE_0: the inner potential correction; R factor indicates the goodness of the fit. S_0^2 was fixed to 0.90, according to the experimental EXAFS fit of Ag foil by fixing CN as the known crystallographic value. Error bounds that characterize the structural parameters obtained by EXAFS spectroscopy were estimated as CN ± 20%, R ± 1%, σ^2 ± 20%.

Supplementary Table 3. R_s and R_{ct} values of MXene, HMX/Ag, and i-HMX@Ag_{20} electrodes.

Electrodes	R_s (Ω)	R_{ct} (Ω)
MXene	3.34	11.39
HMX/Ag	5.03	2.21
i-HMX@Ag_{20}	2.39	0.75

Supplementary Table 4. Comparisons of the dechlorination performance of i-HMX@Ag_{20} film electrode with other faradaic anodes, including Ag-based electrodes, MXene-based electrodes, and other dechlorination electrodes.

Electrode materials	NaCl concentrations (mg L^{-1})	Voltages (V) or specific current (mA g^{-1})	SAC (mg g^{-1})	Reference
CNT@NiCoAl-LDH@MXene	500	1.2 V	79.5	1
CoFe-LDH/$Ti_3C_2T_x$	1168	50 mA g^{-1}	129.09 ± 3.62	2
FeOCl/$Ti_3C_2T_x$	1000	200 mA g^{-1}	55.62 ± 2.61	3
Bi-ene NSs@MXene	1168	1.2 V	88.2	4
MCNF@PPy^+Cl^-	500	1.2 V	91.2	5
NPC/NiMn-LDH/MXene	500	1.2 V	43.5	6
$Ti_3C_2T_x$/Ag-3	584	20 mA g^{-1}	135	7
Ag@CNP	824	1.2 V	77.73	8
Ag@CN	824	1.2 V	110	9
Ag-MOF	500	20 mA g^{-1}	121.52	10
Ag/N-CNP	824	1.2 V	75.3	11
Ag@NHC	500	1.4 V	55.7	12
F-Ag_2@NG	500	1.2 V	~100	13
Ag/ZCs	500	1.2 V	29.18	14
BiOCl@G	500	1.4 V	87	15
CuAl-LDO/rGO	1000	1.2 V	64	16
TAPArGO-75	500	100 mA g^{-1}	55	17
FeS@carbon nanoflowers	500	1.2 V	64.47 ± 0.97	18
Fe/Fe_3C@CNFs	824	1.2 V	126.4	19
Bi/N-PC	1000	100 mA g^{-1}	113.4	20
GF@FeN	1000	1.6 V	140.32	21
i-HMX@Ag_{20}	500	1.2 V	133.34 ± 4.4	This work
i-HMX@Ag_{20}	500	1.4 V	156.63 ± 2.6	This work

Supplementary References

1. Zhang, L. et al. Heterostructure of NiCoAl-layered double hydroxide nanosheet arrays assembled on MXene coupled with CNT as conductive bridge for enhanced capacitive deionization. *Chem. Eng. J.* **478**, 147270 (2023).
2. Lei, J., Xiong, Y., Yu, F. & Ma, J. Flexible self-supporting CoFe-LDH/MXene film as a chloride ions storage electrode in capacitive deionization. *Chem. Eng. J.* **437**, 135381 (2022).
3. Lei, J. et al. Interlayer structure manipulation of FeOCl/MXene with soft/hard interface design for safe water production using dechlorination battery deionization. *Angew. Chem. Int. Ed.* **63**, e202401972 (2024).
4. Gong, S. et al. Vertically aligned bismuthene nanosheets on MXene for high-performance capacitive deionization. *ACS Nano* **17**, 4843-4853 (2023).
5. Wang, X. et al. Hierarchical MXene/polypyrrole-decorated carbon nanofibers for asymmetrical capacitive deionization. *ACS Appl. Mater. Interfaces* **14**, 53150-53164 (2022).
6. Cai, Y., Zhao, G., Yuan, Q. & Zhao, J. Elaborate designed sandwich structural faradic material NPC/NiMn-LDH/MXene for enriched ion accessible transfer pathways in capacitive deionization. *Chem. Eng. J.* **484**, 149491 (2024).
7. Liang, M. et al. Combining battery-type and pseudocapacitive charge storage in Ag/$Ti_3C_2T_x$ MXene electrode for capturing chloride ions with high capacitance and fast ion transport. *Adv. Sci.* **7**, 2000621 (2020).
8. Cao, Y. et al. Enhancement of interfacial interaction between carbon and silver nanoparticles through co-doping with nitrogen and phosphorus. *Desalination* **581**, 117603 (2024).
9. He, Y. et al. Significant stability enhancement of Ag@carbon electrode based on physical confinement of carbon nanocage and its application in capacitive deionization. *Sep. Purif. Technol.* **340**, 126564 (2024).
10. Wei, D. et al. Coordination confined silver-organic framework for high performance electrochemical deionization. *Adv. Sci.* **11**, 2401174 (2024).
11. He, Y. et al. A newly synthesized highly stable Ag/N-carbon electrode for

enhanced desalination by capacitive deionization. *Environ. Sci.: Nano* **7**, 3007-3019 (2020).

12. Kong, W. et al. Highly dispersed Ag nanocrystals anchored on N-doped holey carbon aerogel as high-mass-loading electrode for Cl^- capture in hybrid capacitive deionization. *Small* **21**, 2409342 (2025).
13. Liu, L. et al. Battery-type desalination behavior confined within capsules for efficient capacitive deionization. *Small* **21,** 2408922 (2025).
14. Zhang, H. et al. Ag-doped hollow ZIFs-derived nanoporous carbon for efficient hybrid capacitive deionization. *Desalination* **473**, 114173 (2020).
15. Kong, W. et al. Highly dispersed ultrasmall BiOCl nanoclusters on graphene sheets as high-performance anion-capture electrode for hybrid capacitive deionization. *Desalination* **573**, 117222 (2024).
16. Xi, W. & Li, H. Vertically-aligned growth of CuAl-layered double oxides on reduced graphene oxide for hybrid capacitive deionization with superior performance. *Environ. Sci.: Nano* **7**, 764-772 (2020).
17. Xu, L. et al. Heterointerfacial charge modulation of p-type covalent organic frameworks on graphene achieving high-performance clstorage with ultralong cycling life. *Angew. Chem. Int. Ed.* **64**, e202508092 (2025).
18. Ni, J. et al. Polypropylene-derived iron sulfide/carbon nanoflower for high performance electrochemical dechlorination. *J. Colloid Interface Sci.* **705**, 139491 (2026).
19. Zhang, H. et al. Unlocking efficient redox dechlorination through Fe/Fe_3C nanoclusters embedded porous carbon nanofibers using capacitive deionization. *Sep. Purif. Technol.* **362**, 131944 (2025).
20. Shi, W. et al. Bismuth nanoparticle-embedded porous carbon frameworks as a high-rate chloride storage electrode for water desalination. *ACS Appl. Mater. Interfaces* **13**, 21149-21156 (2021).
21. Liu, L. et al. Pseudocapacitive Fe_4N nanoparticles encapsulated within interconnected graphene cavities as efficient binder-free capacitive deionization anodes. *Desalination* **617**, 119453 (2026).
