# Supplementary material for: Covalent Ag-holey MXene link at interface boosting electrochemical dechlorination
Source: Nat Commun. 2026 Jul 14;17:8642. doi: 10.1038/s41467-026-75560-1 (PMC13486493; doi:10.1038/s41467-026-75560-1)
Supplement: Supplementary file 2 [file 41467_2026_75560_MOESM2_ESM.pdf]

nature portfolio

Peer Review File

Covalent Ag-holey MXene link at interface boosting electrochemical dechlorination

Corresponding Author: Professor Jiayin Yuan

This file contains all reviewer reports in order by version, followed by all author rebuttals in order by version.

Version 0:

Reviewer comments:

Reviewer #1

(Remarks to the Author)
This manuscript presents an in situ etching-and-reduction strategy for constructing covalently linked Ag single-atom/nanoparticle–holey MXene heterointerfaces. The authors provide comprehensive structural and spectroscopic characterization and demonstrate impressive dechlorination performance in both simulated and actual complex saline solution. The study is interesting in its precise regulation of covalent crosslinking between Ag and holey MXene to alter charge distribution at the heterointerface. The combination of experimental techniques with DFT calculations also offers valuable mechanistic insights. Overall, this work represents a meaningful advance in the design of high-performance faradaic electrodes for electrochemical dechlorination. I believe the manuscript could be suitable for publication after minor revision and addressing the issues listed below.
1.The authors should elaborate on the distinction between their in situ method and other non-physical mixing approaches for MXene/Ag composites. A brief comparison would help readers more clearly appreciate the uniqueness of the covalent bridging strategy.
2.Is there any fundamental difference between in situ etching with Ag^+ and conventional H_2O_2 etching? Will different perforation methods affect the structure of holey MXene?
3.During the in situ etching and reduction process, the low-valent Ti species (Ti^{2+}/Ti^{3+}) are expected to participate in redox chemistry. Could this process lead to the formation of Ti-based impurities (e.g., TiO_2), that may affect the purity of the resulting i-HMX@Agx materials? Additional clarification or supporting evidence would be helpful.
4.The current sample notation (i-HMX@Agx) is based on the volume of $AgNO_3$ added during synthesis. For ease of comparison with prior reports, it would be helpful to provide the actual Ag loading (for example, wt% from ICP-OES or another quantitative method) for representative samples.
5.In Fig. 1e, the peak at 446 nm is assigned to the LSPR of Ag NPs. However, for i-HMX@Ag1, this peak is not clearly visible. Could the authors comment on the approximate size threshold for Ag NPs to exhibit LSPR in this system?
6.In Fig. 2a, the Ti 2p negative shift observed for i-HMX@Ag20 is absent for HMX/Ag. A brief statement clarifying why this shift is not seen in the physically mixed control would reinforce that the electronic modulation arises from covalent bonding.
7.The Zeta potential values (Supplementary Fig. S9) for all samples are highly negative (> –40 mV). The authors should discuss whether the presence of Ag NPs or the holey structure influences the surface charge, and whether this has any implication for the colloidal stability during film fabrication.
8.The long-term cycling stability (Fig. 4f) shows > 90% retention over 50 cycles with negligible Ag leaching (6.2 μg/L). Whether any structural changes were observed after prolonged cycling? Post-cycling analysis would be informative to support the robustness of the covalent interface.
9.For the circulating cooling water, the initial Cl^- concentration is reported as 863.3 mg/L. Providing additional details on the water composition (e.g., other coexisting ions, pH) would help readers assess practical applicability.
10.Can the covalent interfacial bridging strategy be extended to be applicable to other MXene-based composite systems besides Ag? A more explicit summary of the key design principles at the end of the Discussion section is needed.

Reviewer #2

(Remarks to the Author)
Title: Covalent Ag-MXene link at interface boosting electrochemical dechlorination

This work proposes an in-situ etching reduction strategy to construct a hybrid structure of Ag single atoms coexisting with

nanoparticles on MXene, emphasizing the formation of a covalent bonding interface between Ag and MXene. The author systematically characterized the material structure, chemical state, electrochemical performance, and CDI dechlorination performance, and combined DFT calculations to explore the enhanced mechanism of interface electronic structure on Cl- adsorption. The overall experimental design is relatively complete, the data is abundant, and some results have certain novelty and application potential. A Major Revision is recommended:

1. The article points out that Ag+ undergoes spontaneous redox reactions with low-priced Ti on the surface of MXene, leading to the formation of Ti defects and pores. Similar phenomena have been reported in existing literature, such as MXene reducing metal ions and forming defects. The author should provide a clearer explanation of the irreplaceability of this work in mechanism control, structural regulation, or performance enhancement. Suggest adding comparative experiments, such as using chemical reducing agent (NaBH4) to prepare Ag/MXene, to compare its interface structure and electrochemical behavior.

2. The term "Ag single atom as growth fulcrum" needs to be more cautious. Although scattered bright spots can be seen in the HAADF-STEM image, the EXAFS fitting results show that the Ag Ag coordination number reaches 5.0, indicating that Ag mainly exists in the form of nanoparticles. There is still a lack of evidence from time-resolved or in situ nucleation processes to determine whether single atoms truly play a role in guiding growth. Suggest the authors to appropriately weaken this statement or supplement the structural evolution data when controlling for extremely low Ag loading.

3. Figure 4f only shows a capacity retention rate of >90% after 50 cycles, but does not provide structural characterization after cycling (such as SEM, XRD, Ag leaching). Suggest supplementing the morphology, crystal structure, and Ag+ leaching concentration of the electrode after cycling (6.2 μg L-1 is already available, and its comparative significance with environmental standards can be further discussed).

4. When treating actual tap water and circulating cooling water in Figure 4h and i, it does not indicate whether pretreatment is carried out (such as filtration, pH adjustment, removal of organic matter, etc.), nor does it indicate the type and aging state of IEM. It is suggested to supplement this information to improve the reproducibility of the results.

5. More experimental evidence is needed to verify the existence of interface electric fields, such as Kelvin probe force microscopy (KPFM).

6. Please provide the b-value and proportion of pseudocapacitance for the comparative sample.

Reviewer #3

(Remarks to the Author)
This work utilizes Ag single atoms as growth fulcrums to construct covalently linked Ag/MXene hybrid structures. The in situ etching–reduction strategy enables strong interfacial coupling between Ag species and MXene while simultaneously generating a porous MXene framework with enhanced ion transport pathways. The combination of Ag single atoms and nanoparticles further maximizes the utilization of redox active sites while mitigating volume changes. Furthermore, the authors provide a comprehensive understanding of the mechanism through a combination of electrochemical analysis and theoretical calculations, and validate its good performance in both model and practical water systems. In summary, this work highlights a rational and multifunctional design strategy that combines interfacial chemistry with ion transport optimization for application in the field of capacitive deionization. The manuscript could be considered for publication after addressing the following comments.

1. The authors repeatedly emphasize that the designed Ag/MXene architecture can effectively mitigate the severe volumetric expansion of Ag during the Ag/AgCl conversion process. However, there appears to be a lack of direct experimental evidence to substantiate this claim. For example, no in situ or ex situ characterization is provided to explicitly demonstrate the suppression of volume expansion. Therefore, it would be important to include more direct evidence to support this key advantage of the proposed design.
2. How can the authors further quantitatively distinguish the actual capacity contribution from the Ag/AgCl conversion reaction? In particular, is it possible that a "pseudo-capacity amplification" effect, similar to that observed in conversion-type electrodes, leads to an overestimation of the faradaic contribution?
3. The authors attribute the significantly reduced Rct to the synergistic effect of the Ag–HMX interface. However, EIS parameters can also be strongly affected by structural factors, such as pore structure, film thickness, and electrode architecture. Therefore, it is not clear whether the reduced Rct mainly originates from interfacial electronic coupling or from structural optimization. Additional evidence or control experiments would be helpful to distinguish between these contributions.
4. The enhanced dechlorination performance is attributed to "electron-deficient Ag facilitating faradaic dechlorination." However, based on the CDI results, the system still exhibits typical capacitive behavior. How do the authors distinguish and quantitatively determine the respective contributions of faradaic reactions and electric double-layer adsorption in Cl^- removal? Are there in situ or ex situ analyses to support this mechanism?
5. The cycling stability was evaluated over only 50 cycles, which appears relatively limited. Could the authors provide longer-term cycling data to better assess the durability and practical reliability of the electrode?
6. Considering the relatively high cost of Ag, could the authors provide an evaluation of the economic feasibility of this material? In addition, how does the performance and cost-effectiveness compare with conventional carbon-based CDI electrodes?

Version 1:

Reviewer comments:

Reviewer #1

(Remarks to the Author)
This revised manuscript should be accepted by Nature Communications.

Reviewer #2

(Remarks to the Author)

The manuscript has been well revised. I recommend publishing.

Reviewer #3

(Remarks to the Author)
The authors have addressed the comments raised in my previous review. I have no further comments.

Open Access This Peer Review File is licensed under a Creative Commons Attribution 4.0 International License, which permits use, sharing, adaptation, distribution and reproduction in any medium or format, as long as you give appropriate credit to the original author(s) and the source, provide a link to the Creative Commons license, and indicate if changes were made.
In cases where reviewers are anonymous, credit should be given to 'Anonymous Referee' and the source.
The images or other third party material in this Peer Review File are included in the article's Creative Commons license, unless indicated otherwise in a credit line to the material. If material is not included in the article's Creative Commons license and your intended use is not permitted by statutory regulation or exceeds the permitted use, you will need to obtain permission directly from the copyright holder.
To view a copy of this license, visit https://creativecommons.org/licenses/by/4.0/

Response to Referees' Comments and List of Changes

(Manuscript number: NCOMMS-26-021303)

Reviewer 1#:

General comments:

This manuscript presents an in situ etching-and-reduction strategy for constructing covalently linked Ag single-atom/nanoparticle–holey MXene heterointerfaces. The authors provide comprehensive structural and spectroscopic characterization and demonstrate impressive dechlorination performance in both simulated and actual complex saline solution. The study is interesting in its precise regulation of covalent crosslinking between Ag and holey MXene to alter charge distribution at the heterointerface. The combination of experimental techniques with DFT calculations also offers valuable mechanistic insights. Overall, this work represents a meaningful advance in the design of high-performance faradaic electrodes for electrochemical dechlorination. I believe the manuscript could be suitable for publication after minor revision and addressing the issues listed below.

Response: We sincerely thank Reviewer 1# for the positive and encouraging assessment of our manuscript. In response to Reviewer 1#'s specific comments, we have revised the manuscript accordingly, clarified key descriptions and comparisons, and provided a detailed point-by-point response as follows.

Comment 1: The authors should elaborate on the distinction between their in situ method and other non-physical mixing approaches for MXene/Ag composites. A brief comparison would help readers more clearly appreciate the uniqueness of the covalent bridging strategy.

Response: We thank Reviewer 1# for this valuable suggestion. Indeed, several articles have explored non-physical synthesis methods for MXene/Ag composites in the field of CDI. To better highlight the uniqueness of our covalent bridging strategy, we have added a brief comparison with representative non-physical mixing approaches (*e.g., Adv. Sci.,* ***2020****, 7, 2000621; Nano Energy,* ***2025****, 141, 111135*). The key distinctions are summarized as follows: **(1) MXene state and porosity**. The reported studies typically start from conventional multilayered or partially exfoliated MXene. In contrast, our synthesis begins with monolayer MXene nanosheets. The use of monolayer MXene not only ensures a more uniform and controllable *in situ* etching-and-reduction

process, but also allows us to precisely engineer in-plane holes and vacancies. Therefore, we simultaneously generated abundant Ti vacancies and in-plane nanopores within the MXene basal plane, yielding a holey MXene (HMX) framework. This porous structure provides additional ion-transport highways and increases the accessibility of active sites. The monolayer nature of HMX also facilitates mechanistic studies (*e.g.*, HAADF-STEM observation of single Ag atoms and DFT modeling) because the 2D lattice is fully exposed without interference from layer stacking. **(2) Ag species and presence of AgCl**. In both Li's and Liang's works, the final composites contain a significant amount of AgCl in addition to metallic Ag. For example, Li *et al.* report a Ag/AgCl heterostructure with a calculated Ag:AgCl mass ratio of 2:1, and Liang *et al.* observe residual AgCl in their samples even after short reaction times. By contrast, our optimized i-HMX@Ag_{20} contains predominantly metallic Ag with a small fraction of monoatomic state Ag, and no detectable AgCl phase remains (XRD, Figure 1f). This "Ag-rich" state is beneficial for the reversible Ag to AgCl conversion during dechlorination. **(3) Interface bonding mode**. Importantly, both literature reports describe the Ag nanoparticles as "confined" or "decorated" on MXene surfaces, but they do not provide evidence of a covalent linkage. In our system, XPS (Ti 2p negative shift, Ag 3d splitting) and EXAFS (Ag−C/O coordination peak at ~1.66 Å) directly demonstrate the formation of covalent Ag−C and Ag−O bonds. Moreover, DFT calculations show that substituting a Ti atom with Ag introduces a strong covalent crosslink, which is confirmed by a much more negative formation energy (−4.80 eV) compared to non-covalent contact (−2.97 eV). Therefore, our strategy not only achieves *in situ* growth of Ag but also uniquely combines covalent interfacial bonding, a holey structure derived from monolayer MXene, and the absence of AgCl residue, which together contribute to the significantly enhanced dechlorination performance and stability. This comparison has been added in the revised manuscript.

Page 6 line 8, "Distinct from conventional Ag/MXene composites that often rely on multilayered or non-porous MXene and contain appreciable AgCl residues[30,31], our approach yields a HMX framework from monolayer nanosheets, creates covalent Ag−C/O interfacial bonds, and leaves no detectable AgCl residue, thereby ensuring superior dechlorination performance."

Comment 2: Is there any fundamental difference between in situ etching with Ag^+ and conventional H_2O_2 etching? Will different perforation methods affect the structure of holey MXene?

Response: We thank Reviewer 1# for the insightful comments. Both Ag^+ and H_2O_2 can etch MXene through oxidation of Ti species. In both cases, the oxidant reacts with Ti atoms, creating

Ti vacancies and gradually enlarging them into in-plane holes. The etching also removes some surface terminal groups (*e.g.*, −OH, −O). By-products such as TiO_2-type impurities can be removed by subsequent acid washing, leaving a clean HMX surface. The main differences lie in the reaction kinetics and in whether new species are introduced. H_2O_2 reacts more aggressively. It creates holes relatively fast and introduces no additional metallic species, but its strong oxidation can also remove more functional groups and may lead to a slightly higher concentration of Ti vacancies under the same conditions. By contrast, Ag^+ reacts more mildly and in a more controllable manner. The reduction of Ag^+ by low-valence Ti species deposits Ag atoms directly onto the HMX surface, and these Ag species form covalent bonds (Ag−C/O) with the lattice. This simultaneous etching-reduction process is critical for constructing the covalent heterointerface. Importantly, by carefully controlling the reaction time and concentration, we were able to produce HMX (*via* H_2O_2) and i-HMX@Ag_x (*via* Ag^+) with comparable in-plane pore size and porosity, as demonstrated by TEM (Supplementary Fig. 5 and Supplementary Fig. 7). This structural similarity allows us to attribute the performance differences primarily to the interfacial chemistry rather than to pore architecture. We have clarified these points in the revised manuscript.

Page 8 line 6, "The HMX framework was produced *ex situ via* a H_2O_2-assisted etching treatment[33]. It should be noted that both Ag^+ and H_2O_2 create in-plane holes in MXene through selectively oxidative etching of Ti species, accompanied by the removal of surface terminal groups, but their reaction kinetics differ. By fine-tuning the etching conditions of H_2O_2, the produced HMX exhibits in-plane porosity and thickness comparable to that of i-HMX@Ag_{20} (Supplementary Fig. 7)."

Comment 3: During the in situ etching and reduction process, the low-valent Ti species (Ti^{2+}/Ti^{3+}) are expected to participate in redox chemistry. Could this process lead to the formation of Ti-based impurities (e.g., TiO_2), that may affect the purity of the resulting i-HMX@Ag_x materials? Additional clarification or supporting evidence would be helpful.

Response: We thank Reviewer 1# for raising this concern. During the *in situ* etching-and-reduction process, partial oxidation of low-valence Ti species (Ti^{2+}/Ti^{3+}) can indeed occur, potentially generating TiO_2-type impurities. Taking this into consideration, we have carried out an acid washing process after etching to remove such impurities. To verify this, we have supplemented a more detailed characterization of the i-HMX@Ag_{20} sample before the L(+)-tartaric acid washing. As shown in Supplementary Fig. 3a, the TEM image reveals the presence of spindle-like TiO_2

nanoparticles, and the Raman spectrum (Supplementary Fig. 3b) exhibits a characteristic band at approximately 150 cm^{-1}, both of which are consistent with the formation of TiO_2. However, these TiO_2 impurities can be effectively removed by the subsequent L(+)-tartaric acid washing step. After washing, the TEM and HAADF-STEM images (Fig. 1b, c) of the final i-HMX@Ag$_{20}$ sample exhibit a clean and holey surface without impurities, and the XRD pattern (Fig. 1f) shows no detectable TiO_2 diffraction peaks. These results confirm that the generated TiO_2 by-products are completely eliminated, leaving a clean HMX surface. Therefore, the purity of the i-HMX@Ag$_x$ materials is not compromised. A brief discussion has been added to the revised manuscript.

Page 6 line 17, "Of note, the oxidation of low-valence Ti species during the *in situ* etching-and-reduction process inevitably produces TiO_2-type by-products, as demonstrated by the spindle-like TiO_2 in transmission electron microscopy (TEM) image and a characteristic band at approximately 150 cm^{-1} in Raman spectrum (Supplementary Fig. 3). Therefore, L(+)-tartaric acid was employed as a mild scavenger to eliminate these impurities and ensure the purity of the i-HMX@Ag$_x$. In contrast, the TEM image of i-HMX@Ag$_{20}$ (Fig. 1b) shows numerous in-plane nanoholes with Ag NPs firmly attached to the clean surface of HMX after the acid washing process."

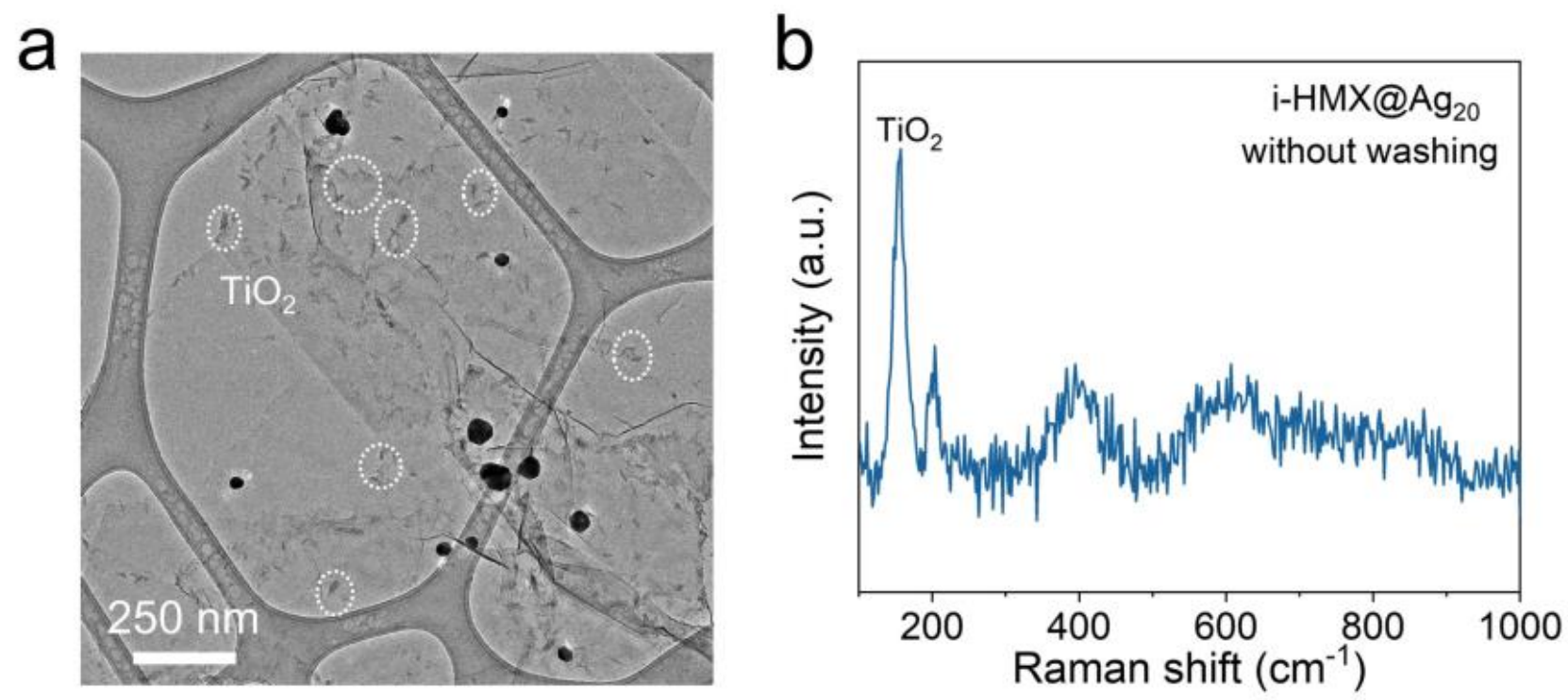

Supplementary Fig. 3. (a) TEM image and **(b)** Raman spectrum of i-HMX@Ag$_{20}$ before washing with L(+)-tartaric acid.

Comment 4: The current sample notation (i-HMX@Ag$_x$) is based on the volume of $AgNO_3$ added during synthesis. For ease of comparison with prior reports, it would be helpful to provide the actual Ag loading (for example, wt% from ICP-OES or another quantitative method) for representative samples.

Response: We appreciate Reviewer 1# for this valuable suggestion. We agree that the actual Ag content in different samples does not necessarily equal to the added dosage. Accurate determination

of the Ag loading amount is of great significance for conducting comparisons. In response to this helpful comment, we have now performed ICP-OES analysis after digesting the i-HMX@Ag_x films with nitric acid. The new results summarized in Supplementary Table 1 show that the Ag content increases from 0.22 wt.% in i-HMX@Ag_1 to 13.32 wt.% in i-HMX@Ag_{30}. The lower Ag content observed compared to the theoretical value may be due to incomplete reaction between Ag^+ and MXene and nanoparticle detachment during the washing process. However, this does not affect our core conclusion associated with the gradient Ag content. We have also clarified that the subscript "*x*" in i-HMX@Ag_x refers to mass percentage of the added $AgNO_3$ in relation to MXene, not the actual Ag content, which can intuitively express our gradient experiment design. The detailed experiments and descriptions have been added to the revised manuscript and Supplementary Information.

Page 6 line 6, "Of note, in order to visually represent the design of gradient experiment, the subscript "*x*" in i-HMX@Ag_x indicates the mass percentage of the added $AgNO_3$ in relation to MXene, rather than the actual Ag content."

Page 9 line 4, "Inductively coupled plasma-optical emission spectrometer (ICP-OES) was employed to quantify the actual Ag loading after digesting the i-HMX@Ag_x films with nitric acid. As displayed in Supplementary Table 1, the Ag content gradually increases from 0.22 wt.% in i-HMX@Ag_1 to 13.32 wt.% in i-HMX@Ag_{30} but all remain below the theoretical value, which may be ascribed to the incomplete reaction between Ag^+ and MXene and partial nanoparticle detachment during the washing process."

Page 32 line 5, "Subsequently, the i-HMX@Ag_x (*x* represents the mass percentage of $AgNO_3$ in relation to MXene and is numerically equivalent to the volume of the added $AgNO_3$ solution in mL) dispersion was obtained by scavenging surface impurities with L(+)-tartaric acid solution and rinsing with DI water."

Supplementary Table 1. Theoretical and measured Ag content in different samples.

Samples	Theoretical Ag content (wt.%)	Ag content by ICP-OES (wt.%)
i-HMX@Ag_1	0.64	0.22
i-HMX@Ag_5	3.18	1.35
i-HMX@Ag_{10}	6.35	3.69
i-HMX@Ag_{20}	12.70	8.48
i-HMX@Ag_{30}	19.05	13.32

Comment 5: In Fig. 1e, the peak at 446 nm is assigned to the LSPR of Ag NPs. However, for i-HMX@Ag_1, this peak is not clearly visible. Could the authors comment on the approximate size threshold for Ag NPs to exhibit LSPR in this system?

Response: We thank Reviewer 1# for the constructive comments. Actually, the ultraviolet-visible absorption spectrum of HMX@Ag_1 has a small bulge at 446 nm and a red-shift in the peak at 774 nm compared to MXene. The change gradually intensifies as the size of Ag NPs increases. Following the suggestion, we have measured the mean size of the Ag NPs in HMX@Ag_1 based on the TEM image, which is approximately 20 nm. Such Ag NPs of several tens of nanometers are sufficient to induce localized surface plasmon resonance (LSPR), which has been confirmed in previous work (*e.g., Carbon*, ***2025***, *235, 120025; Phys. Rev. B*, ***2014***, *90, 125417*). The relevant descriptions have been added to the revised manuscript.

Page 7 line 21, "In the ultraviolet-visible (UV-Vis) absorption spectra (Fig. 1e), the progressive attenuation of the absorption peak at 322 nm, characteristic of MXene's interband transitions, indicates a gradual modulation of the MXene electronic structure driven by charge redistribution upon interfacial covalent bonding. Concurrently, a gradually noticeable bulge peak appears at 446 nm, signaling the onset of Ag NPs formation. The mean size of the Ag NPs in HMX@Ag_1 was measured to be approximately 20 nm, which is sufficient to induce localized surface plasmon resonance[38]."

Comment 6: In Fig. 2a, the Ti 2p negative shift observed for i-HMX@Ag_{20} is absent for HMX/Ag. A brief statement clarifying why this shift is not seen in the physically mixed control would reinforce that the electronic modulation arises from covalent bonding.

Response: We thank Reviewer 1# for the valuable comment. In the physically mixed HMX/Ag control sample, there is no covalent bonding between Ag and HMX; thus no electron transfer occurs between Ag and HMX. Consequently, the electron density around Ti atoms does not increase, and the Ti 2p binding energy remains unchanged. We have added a clear statement in the revised manuscript.

Page 14 line 5, "However, no peak shift was observed for HMX/Ag, revealing that no interfacial electron transfer occurred between Ag and HMX. Therefore, the electron density around Ti atoms does not increase and the Ti 2p binding energy remains unchanged in physically mixed HMX/Ag. This result demonstrates that the *in situ* etching-and-reduction process induced the

electronic redistribution at the heterogeneous interface."

Comment 7: The Zeta potential values (Supplementary Fig. S9) for all samples are highly negative (> −40 mV). The authors should discuss whether the presence of Ag NPs or the holey structure influences the surface charge, and whether this has any implication for the colloidal stability during film fabrication.

Response: We appreciate Reviewer 1# for this helpful suggestion and have added a dedicated description regarding Zeta potential values. Briefly, the etching process removed a large number of unsaturated Ti atoms at the edges of MXene, thereby further reducing the Zeta potential. Since the Zeta potential values of all dispersions were lower than −40.0 mV, they were able to maintain excellent colloidal stability throughout the process of film preparation. The corresponding statements have been added to the revised manuscript.

Page 8 line 21, "All prepared samples exhibit highly negative Zeta potentials (more negative than −40.0 mV) in a neutral medium (Supplementary Fig. 10) due to the abundant −OH and −O functional groups on MXene. Notably, the Zeta potentials of i-HMX@Ag_x and HMX/Ag are slightly lower than that of pure MXene (−42.2 ± 2.0 mV) due to the etching of the unsaturated Ti atoms at the edges[33]. As the etching intensity increases, this value gradually decreases from −43.6 ± 0.9 mV of i-HMX@Ag_1 to −48.5 ± 3.3 mV of i-HMX@Ag_{30}. Overall, the high absolute zeta potential values of all dispersions provide strong electrostatic and colloidal stabilization against aggregation, thus ensuring the successful fabrication of uniform, crack-free films."

Comment 8: The long-term cycling stability (Fig. 4f) shows > 90% retention over 50 cycles with negligible Ag leaching (6.2 μg/L). Whether any structural changes were observed after prolonged cycling? Post-cycling analysis would be informative to support the robustness of the covalent interface.

Response: We thank Reviewer 1# for the valuable comment. To further evaluate the structural robustness after prolonged cycling, we extended the cycling test to 100 cycles and performed post-cycling characterizations. As shown in Supplementary Fig. 35, the electrode retains 78.6% of its initial capacity after 100 cycles. The surface SEM (Supplementary Fig. 36a) shows that Ag NPs remain well covered by the HMX nanosheets, similar to the fresh electrode (Fig. 1h). The XRD pattern (Supplementary Fig. 36b) confirms that the characteristic peaks of MXene and metallic Ag are still present, although weak AgCl peaks appear, indicating a minor degree of irreversible phase

transformation after extended operation. Critically, the cross-sectional SEM image and EDS mapping (Supplementary Fig. 36c) demonstrate that the layered film structure and the uniform distribution of Ti, Ag, and C elements are well preserved. The Ag^{+} leaching concentration after 50 cycles (6.2 μg L^{-1}) and after 100 cycles (7.1 μg L^{-1}) are both far below the Chinese drinking water safety limit (0.05 mg L^{-1}, GB 5749-2022) and WHO guideline (100 μg L^{-1}). These results provide direct evidence that the i-HMX@Ag_{20} film electrode remains structurally robust even after 100 cycles. We have supplemented these statements to the revised manuscript.

Page 23 line 8, "Even extending the cycling test to 100 cycles, the i-HMX@Ag_{20} electrode can retain 78.6% of its initial capacity (Supplementary Fig. 35). Post-cycling characterization was carried out to examine its structural changes. As shown in Supplementary Fig. 36a, the Ag NPs remain uniformly covered by the HMX nanosheets, with a surface morphology similar to the fresh electrode (Fig. 1h). In addition to the peaks of MXene and metallic Ag observed in the XRD spectrum (Supplementary Fig. 36b), relatively weak AgCl diffraction peaks were also detected, indicating a minor degree of irreversible phase transformation after prolonged cycling. Nevertheless, its layered film structure remains intact, with homogeneous distribution of Ti, Ag, and C elements throughout the cross-section (Supplementary Fig. 36c). The Ag^{+} leaching concentration detected in the solution after 50 cycles was only 6.2 μg L^{-1}, and after 100 cycles it remained as low as 7.1 μg L^{-1}. Both values are far below the safety limit of 50 μg L^{-1} stipulated by the standards for drinking water quality (GB 5749-2022, China) and are well within the WHO guideline of 100 μg L^{-1}. This extremely low Ag loss confirms that the covalent Ag−HMX interface not only anchors Ag species firmly but also prevents undesirable dissolution, thereby supporting both the structural stability and favorable environmental safety of the i-HMX@Ag_{20} electrode in long-term use."

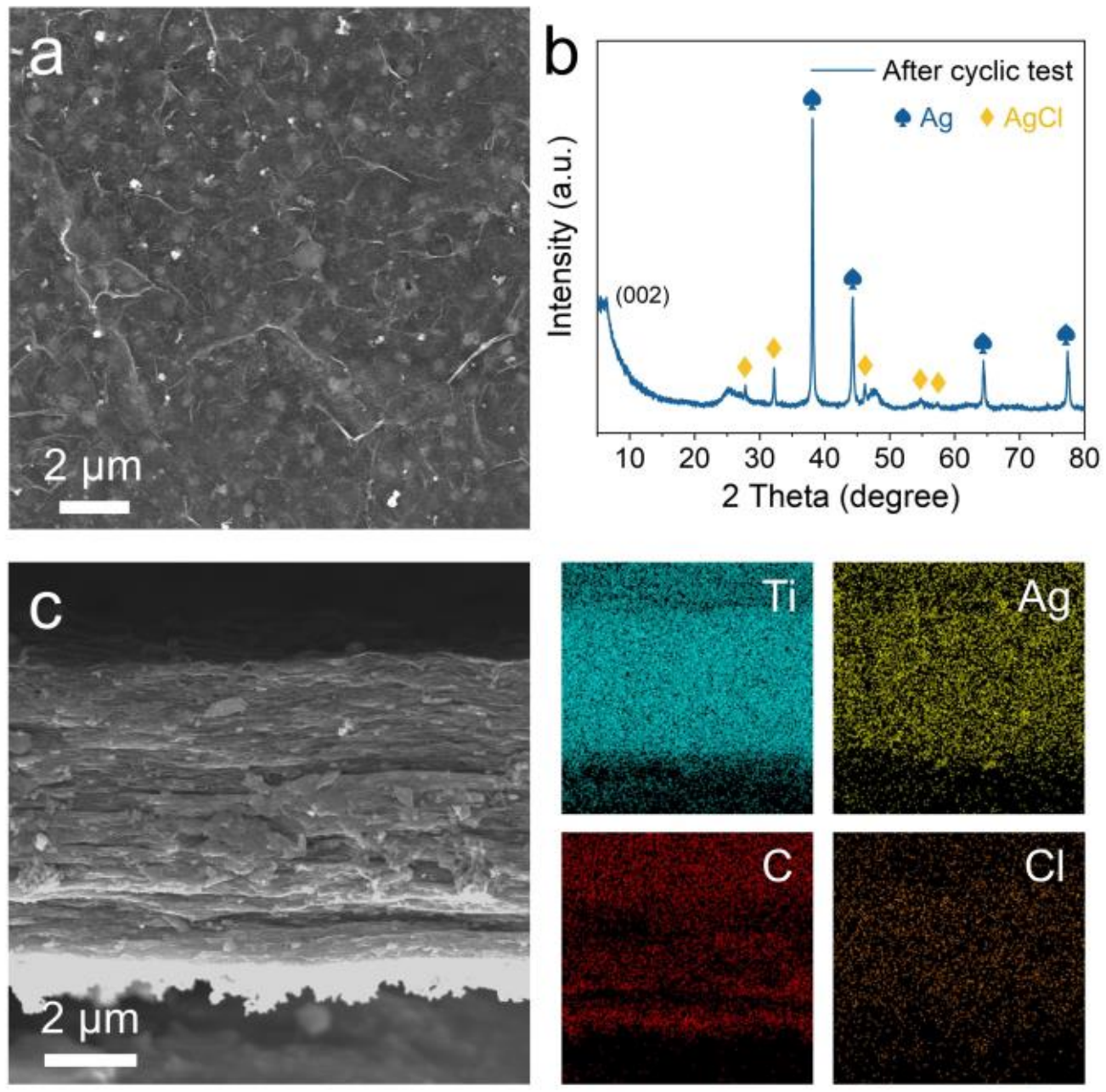

Supplementary Fig. 36. (a) Surface SEM image, **(b)** XRD pattern, and **(c)** cross-sectional SEM image and corresponding EDS elemental mapping of i-HMX@Ag_{20} film after cyclic test.

Comment 9: For the circulating cooling water, the initial Cl^- concentration is reported as 863.3 mg/L. Providing additional details on the water composition (e.g., other coexisting ions, pH) would help readers assess practical applicability.

Response: We thank Reviewer 1# for raising this important point. The circulating cooling water used in this study was configured by dissolving 0.83 g of NaCl, 0.46 g of $CaCl_2$, 0.19 g of $MgCl_2 \cdot 6H_2O$, and 0.27 g $NaHCO_3$ in 1 L of deionized water. Specifically, NaCl serves as the main source of Cl^-, Ca^{2+} and Mg^{2+} provide the hardness of water, and $NaHCO_3$ is buffer to maintain the pH value of water at a slightly alkaline level. The final water composition exhibits the typical characteristics of circulating cooling water and can be utilized to explore the practical application potential of the electrodes. Similar research methods regarding practical applicability have also been reported in previous literature (*e.g., Angew. Chem. Int. Ed.*, ***2025***, *64*, *e202504775*). We have provided additional details on the water composition in the revised manuscript.

Page 34 line 13, "In the practical applicability experiments, the tap water was directly supplied by the municipal source. The circulating cooling water was simulated by dissolving 0.83 g of NaCl, 0.46 g of $CaCl_2$, 0.19 g of $MgCl_2 \cdot 6H_2O$, and 0.27 g $NaHCO_3$ in 1 L of DI water, where NaCl is the main source of Cl^-, Ca^{2+} and Mg^{2+} provide the hardness of water, and $NaHCO_3$ is buffer to maintain the pH value of water at a slightly alkaline level."

Comment 10: Can the covalent interfacial bridging strategy be extended to be applicable to other MXene-based composite systems besides Ag? A more explicit summary of the key design principles at the end of the Discussion section is needed.

Response: We thank Reviewer 1# for this constructive suggestion. The key design principle of our *in situ* etching-and-reduction strategy relies on the spontaneous redox reaction between noble metal cations and the low-valence Ti species (Ti^{2+}/Ti^{3+}) on MXene. Therefore, metal ions that can be reduced by Ti^{2+}/Ti^{3+} (*e.g.*, Pd^{2+}, Au^{3+}) are in principle applicable to this strategy. As a proof-of-concept, we replaced $AgNO_3$ with palladium chloride ($PdCl_2$) under otherwise identical conditions and successfully prepared a MXene/Pd-based composite material, denoted as i-HMX@Pd. As shown in Supplementary Fig. 19a, the TEM image reveals that Pd nanoparticles are uniformly anchored on the surface of holey MXene nanosheets. The high-resolution TEM (HRTEM) clearly shows lattice fringes corresponding to metallic Pd (Supplementary Fig. 19b). The XRD pattern (Supplementary Fig. 19c) exhibits distinct diffraction peaks of Pd along with the (002) peak of MXene, analogous to the i-HMX@Ag case. Moreover, HAADF-STEM and corresponding EDS elemental mapping (Supplementary Fig. 19d-i) demonstrate homogeneous distribution of Ti, C, O, F, and Pd across the entire nanosheet. These results collectively confirm that our covalent interfacial bridging strategy is not limited to Ag but can be extended to other noble metals such as Pd. We have added a brief statement on the generalizability of this strategy in the revised manuscript.

Page 13 line 12, "The generality of this strategy was further explored by replacing Ag^{+} with Pd^{2+} under identical conditions. As shown in Supplementary Fig. 19a, the TEM image of the prepared i-HMX@Pd reveals that numerous Pd NPs are attached to the HMX. The high-resolution TEM (HRTEM) clearly shows lattice fringes corresponding to metallic Pd (Supplementary Fig. 19b). Moreover, the XRD pattern of i-HMX@Pd (Supplementary Fig. 19c) exhibits distinct diffraction peaks of Pd along with the (002) peak of MXene, which is similar to the i-HMX@Ag_x samples. The HAADF-STEM and corresponding EDS elemental mapping (Supplementary Fig. 19d-i) further demonstrate the uniform distribution of Pd across the entire nanosheet. Collectively, these results demonstrate that this *in situ* etching-and-reduction strategy can be extended to other noble metals, such as Pd, that can undergo spontaneous redox reactions with the low-valence Ti species on MXene, which offers a general platform for constructing covalently linked metal/MXene heterointerfaces for diverse electrochemical applications."

Page 30 line 21, "This strategy relies on the spontaneous redox reaction between metal cations and the low-valence Ti species on MXene, and can be extended to other noble metals, such as Pd."

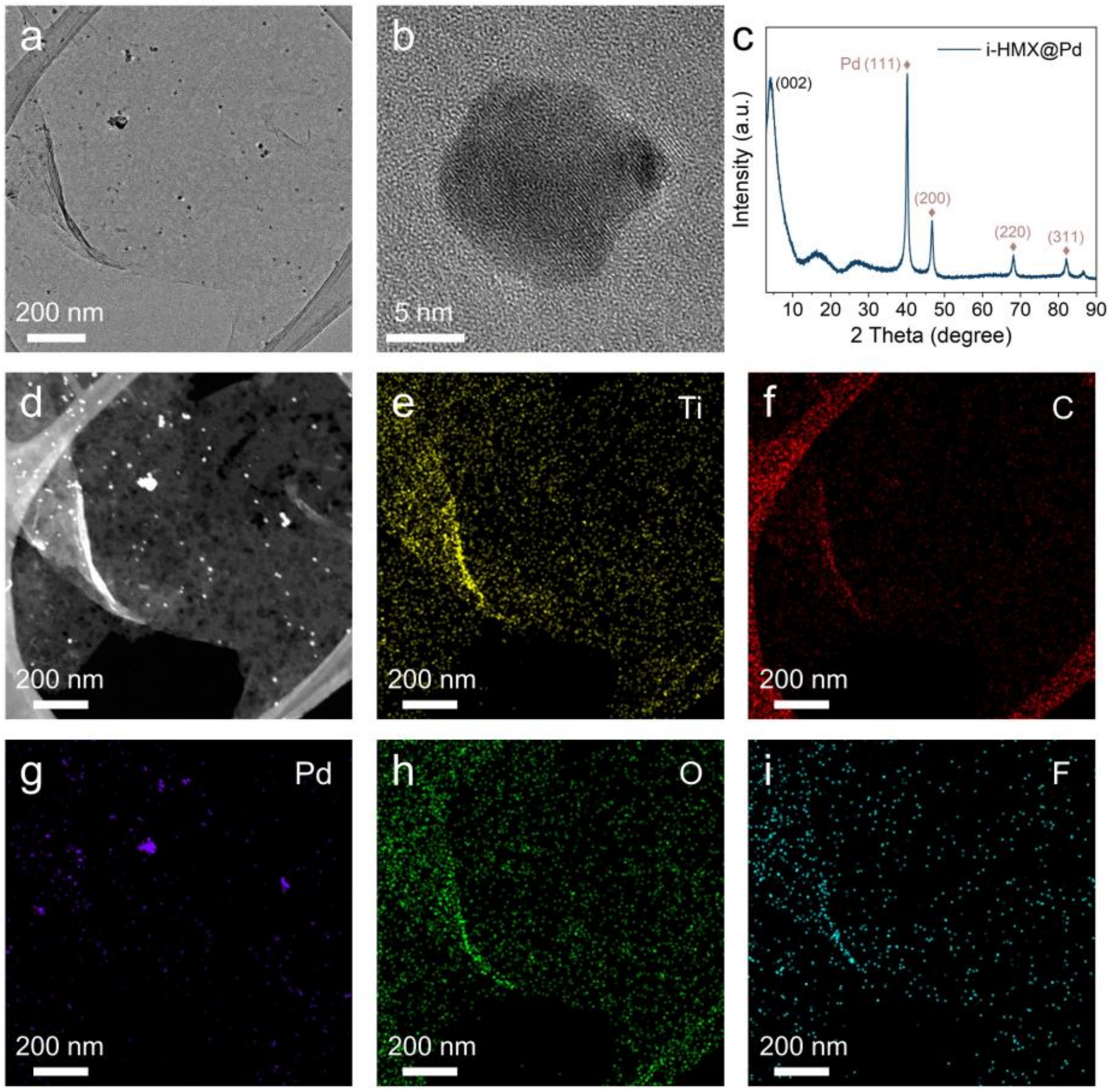

Supplementary Fig. 19. (a) TEM image, **(b)** HRTEM image, **(c)** XRD pattern, **(d-i)** HAADF-STEM and corresponding EDS elemental mapping images of i-HMX@Pd.

Reviewer 2#:

General comments:

This work proposes an in-situ etching reduction strategy to construct a hybrid structure of Ag single atoms coexisting with nanoparticles on MXene, emphasizing the formation of a covalent bonding interface between Ag and MXene. The author systematically characterized the material structure, chemical state, electrochemical performance, and CDI dechlorination performance, and combined DFT calculations to explore the enhanced mechanism of interface electronic structure on Cl^- adsorption. The overall experimental design is relatively complete, the data is abundant, and some results have certain novelty and application potential. A Major Revision is recommended:

Response: We sincerely appreciate Reviewer 2# for the positive evaluation of our manuscript and for recognizing the systematic design, novelty, and potential practical relevance of this study. We greatly appreciate the reviewer's constructive comments and suggestions, which have helped us further improve the quality and clarity of the manuscript. In response to Reviewer 2#'s specific comments, we have carefully revised the manuscript accordingly and provided our detailed point-by-point response below.

Comment 1: The article points out that Ag^+ undergoes spontaneous redox reactions with low-priced Ti on the surface of MXene, leading to the formation of Ti defects and pores. Similar phenomena have been reported in existing literature, such as MXene reducing metal ions and forming defects. The author should provide a clearer explanation of the irreplaceability of this work in mechanism control, structural regulation, or performance enhancement. Suggest adding comparative experiments, such as using chemical reducing agent ($NaBH_4$) to prepare Ag/MXene, to compare its interface structure and electrochemical behavior.

Response: We appreciate Reviewer 2# for the constructive suggestion. We fully agree that the spontaneous redox reaction between Ag^+ and MXene has been reported previously. The irreplaceability of our work, however, does not lie in the discovery of this reaction itself, but rather in the deliberate kinetic control of the reaction to achieve covalent interfacial bonding between Ag and HMX, which is fundamentally different from simple physical deposition or weak adsorption. The covalent Ag–HMX interface induces charge redistribution and creates an electron-deficient Ag surface, which favors the faradaic dechlorination reaction ($Ag + Cl^- \rightleftharpoons AgCl + e^-$).

To further substantiate the unique role of our covalent bridging strategy, we carried out a control experiment by adding a strong chemical reductant ($NaBH_4$) to the synthesis system of i-HMX@Ag_{20}, while keeping all other conditions identical. The results clearly show that external chemical reduction disrupts the formation of the desired covalent interface. As shown in Supplementary Fig. 32a, b, the TEM images of i-HMX@Ag_{20}-$NaBH_4$ reveal Ag NPs and some in-plane holes on MXene, indicating that the spontaneous redox reaction between Ag^+ and MXene still occurs to some extent. However, the UV-Vis absorption spectra (Supplementary Fig. 32c) show that the peak at 774 nm shifts only to 779 nm, which is much smaller than that observed for i-HMX@Ag_{20} (to 800 nm), indicating a much weaker interfacial interaction between Ag and MXene. The XRD pattern (Supplementary Fig. 32d) confirms the presence of both Ag and MXene (002) peaks, further verifying the successful formation of Ag NPs. However, the Ti 2p XPS

spectrum (Supplementary Fig. 32e) shows that the negative shift of the peaks is much smaller compared to that of the i-HMX@Ag_{20}. The proportion of Ag^+ species in Ag 3d XPS spectrum of i-HMX@Ag_{20}-$NaBH_4$ is also much lower (Supplementary Fig. 32f). These results indicate that most Ag^+ ions are rapidly reduced by $NaBH_4$ into Ag NPs, which subsequently attach to the MXene surface mainly through physical interactions rather than covalent bonding. Consequently, the $NaBH_4$-treated sample exhibits inferior electrochemical performance (smaller CV peak currents, lower specific capacitance, shorter GCD discharge times) compared to i-HMX@Ag_{20} (Supplementary Fig. 32g-i).

These results collectively demonstrate that our *in situ* etching-and-reduction strategy, without any external reductant, uniquely promotes the formation of covalent Ag−C/Ag−O bonds at the heterointerface, which cannot be achieved by simply adding a chemical reductant. This controlled interfacial chemistry is the key to the superior performance of our electrode. Following the reviewer's suggestion, we have added the corresponding discussion and data in the revised manuscript and Supplementary Information.

Page 20 line 3, "To further verify the critical role of our *in situ* etching-and-reduction strategy in boosting electrochemical performance, we set up a control sample by introducing $NaBH_4$, a strong reducing agent, into the synthesis of i-HMX@Ag_{20} while keeping all other parameters unchanged. Interestingly, TEM images of the $NaBH_4$-treated sample (Supplementary Fig. 32a, b) still exhibit Ag NPs and some in-plane holes, suggesting that the redox reaction between Ag^+ and MXene still occurs. However, the UV-Vis absorption peak at 774 nm shifts only to 779 nm (Supplementary Fig. 32c), compared to 800 nm for i-HMX@Ag_{20}, indicating a much weaker interfacial interaction between Ag and MXene. The XRD pattern exhibits characteristic peaks of both metallic Ag and MXene (Supplementary Fig. 32d), indicating the successful formation of Ag NPs under the combined reduction effects of MXene and $NaBH_4$. Notably, the Ti 2p XPS spectrum (Supplementary Fig. 32e) shows that the negative shift of the peaks is much smaller than that of the i-HMX@Ag_{20} (Fig. 2a). Moreover, the proportion of Ag^+ species in Ag 3d XPS spectrum of the $NaBH_4$-treated sample is also much lower (Supplementary Fig. 32f). These results suggest that most Ag^+ are rapidly reduced by $NaBH_4$ into Ag NPs that physically adsorb onto the MXene surface, rather than forming covalent bonds. As a result, the $NaBH_4$-treated sample exhibits smaller CV peak currents, lower specific capacitance (196.4 F g^{-1} at 1 mV s^{-1}), and shorter GCD discharge times than i-HMX@Ag_{20} (Supplementary Fig. 32g-i). These observations collectively demonstrate that our *in situ* etching-and-reduction strategy uniquely promotes the formation of covalent

Ag−C/O bonds at the heterointerface, which cannot be achieved by simply adding an external chemical reductant. The controlled interfacial chemistry is essential for the superior electrochemical performance of the i-HMX@Ag_{20} electrode."

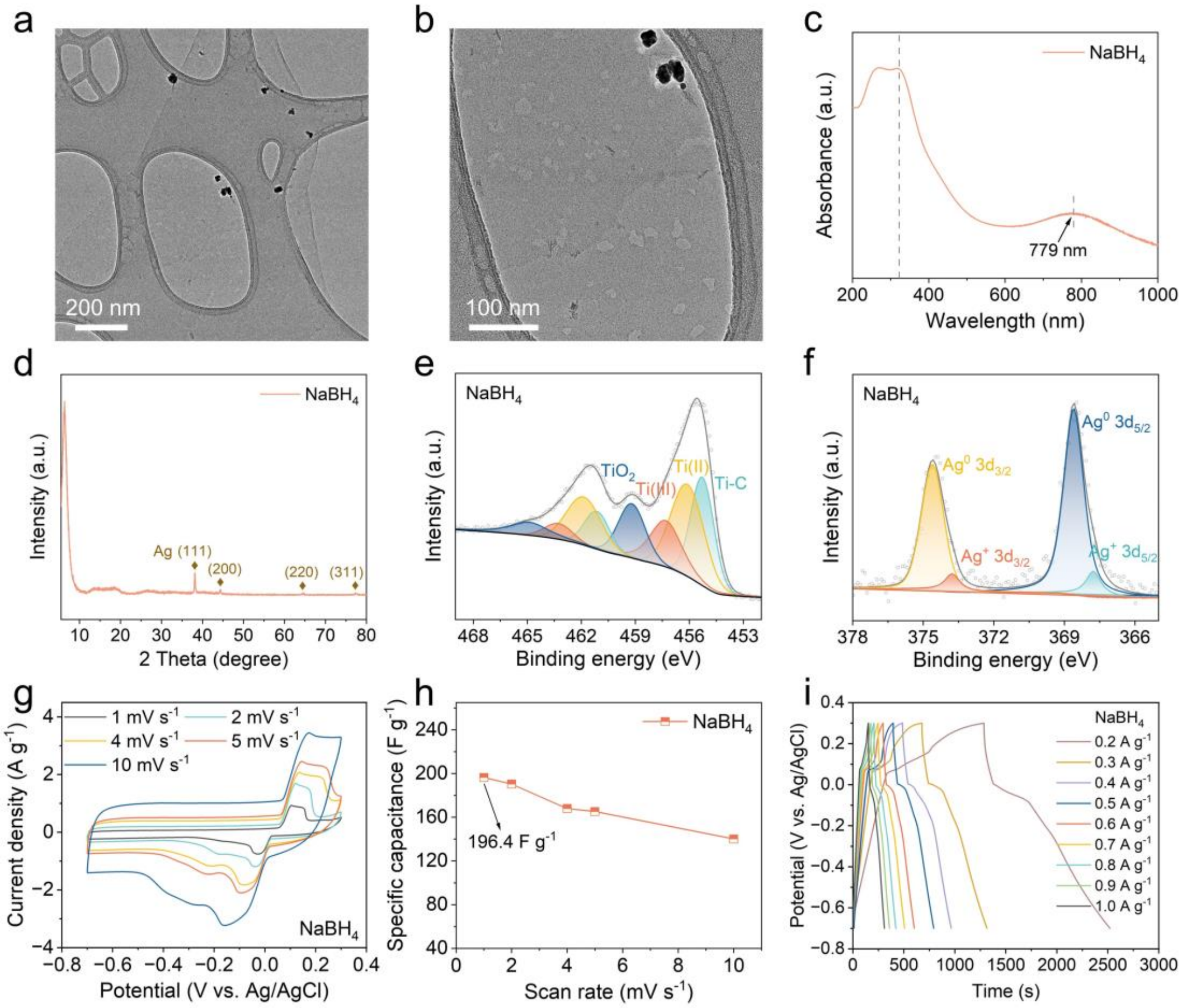

Supplementary Fig. 32. (a, b) TEM images, **(c)** UV-Vis spectrum, **(d)** XRD pattern, **(e)** Ti 2p, and **(f)** Ag 3d XPS spectra of Ag−MXene prepared with $NaBH_4$. **(g)** CV curves and **(h)** corresponding specific capacitances at different scan rates. **(i)** GCD profiles at different current densities.

Comment 2: The term "Ag single atom as growth fulcrum" needs to be more cautious. Although scattered bright spots can be seen in the HAADF-STEM image, the EXAFS fitting results show that the Ag Ag coordination number reaches 5.0, indicating that Ag mainly exists in the form of nanoparticles. There is still a lack of evidence from time-resolved or in situ nucleation processes to determine whether single atoms truly play a role in guiding growth. Suggest the authors to appropriately weaken this statement or supplement the structural evolution data when controlling for extremely low Ag loading.

Response: We thank Reviewer 2# for the valuable and constructive comment. We agree that the phrase "Ag single atoms as growth fulcrums" should be used more cautiously, given the current level of evidence. In the original manuscript, this interpretation was mainly based on the gradual evolution of the UV-Vis absorption peaks and the XRD patterns with the increase in Ag^+ dosage, where the signals of Ag nanoparticles gradual intensified from i-HMX@Ag_1 to i-HMX@Ag_{30}. Following the reviewer's suggestion, we have prepared a transition sample with an extremely low Ag loading (termed i-HMX@$Ag_{0.1}$). As shown in Supplementary Fig. 12a, the HAADF-STEM image of i-HMX@$Ag_{0.1}$ shows several isolated bright spots corresponding to atomically dispersed Ag single atoms, along with nanoscale defects on MXene surface. This indicates that Ag^+ is preferentially reduced and immobilized in a monatomic form. More importantly, no characteristic peaks of metallic Ag are observed in the XRD pattern of i-HMX@$Ag_{0.1}$ (Supplementary Fig. 12b), demonstrating that at a low Ag loading, Ag remains predominantly in a highly dispersed state rather than growing into detectable nanoparticles. Therefore, it can be reasonably inferred that Ag^+ was first *in situ* reduced by the unsaturated Ti species and deposited on the surface of MXene, and then gradually grew into nanoparticles as the Ag^+ concentration increases, which is consistent with the statement in our manuscript. Nevertheless, in light of the reviewer's comment, we have supplemented these structural evolution data to the revised manuscript and provided more appropriate expressions for the related contents to avoid overinterpretation.

Page 9 line 17, "To further explore the growth process of Ag NPs, a transition sample with a very low Ag dosage was prepared by adding $AgNO_3$ and MXene at a mass percentage of 0.1% (termed i-HMX@$Ag_{0.1}$). As exhibited in Supplementary Fig. 12a, the HAADF-STEM image of i-HMX@$Ag_{0.1}$ reveals several scattered bright spots, ascribing to the immobilized atomically dispersed Ag SAs through *in situ* reduction. This reaction process also led to some nanoscale defects on the surface of MXene. It is worth noting that no characteristic peaks related to metallic Ag were observed in the XRD spectrum of i-HMX@$Ag_{0.1}$ (Supplementary Fig. 12b), suggesting that these small amounts of Ag could not form NPs. These results further indicate that a small amount of Ag^+ is preferentially reduced and fixed as monatomic form on the surface of MXene, and then gradually grows into NPs as the concentration of added Ag^+ increases."

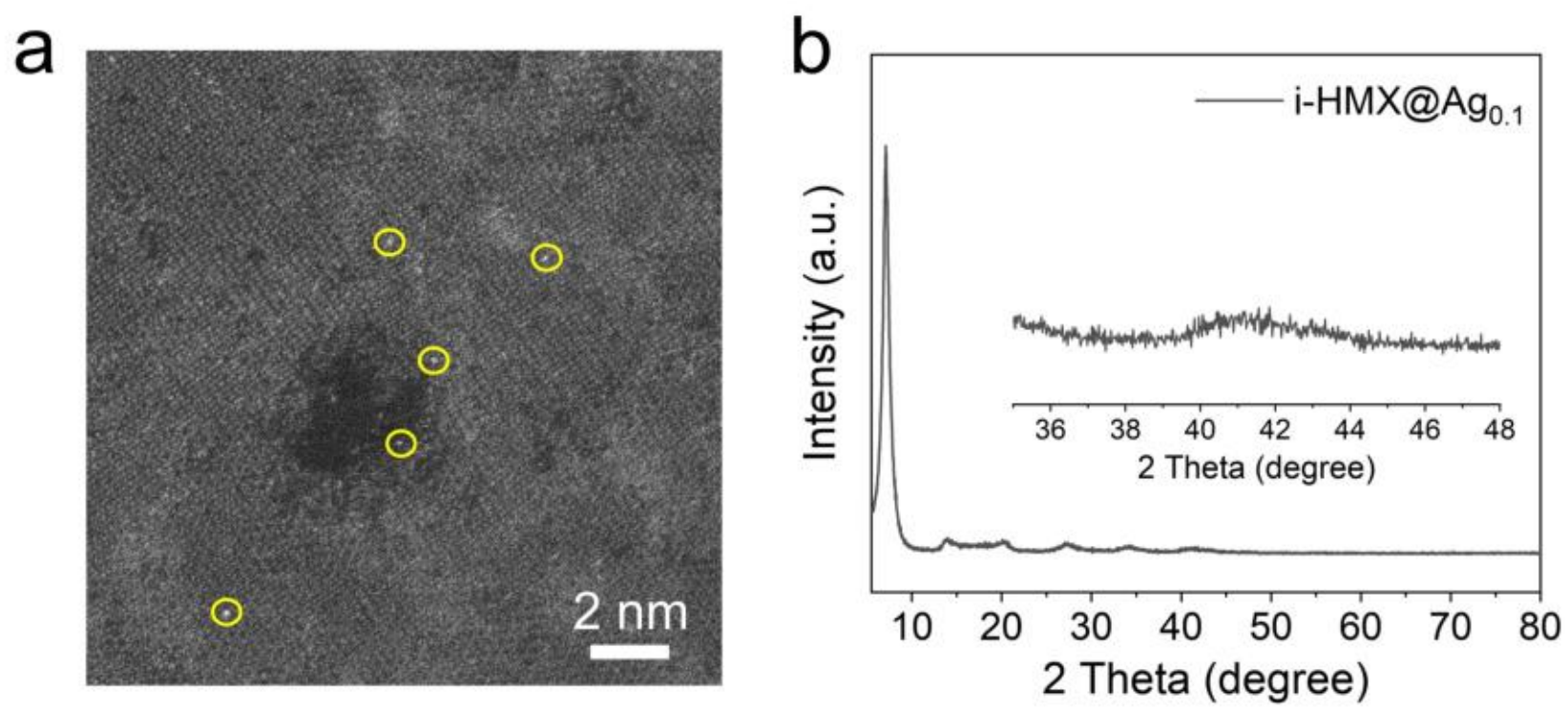

Supplementary Fig. 12. (a) HAADF-STEM image and **(b)** XRD pattern of i-HMX@$Ag_{0.1}$.

Comment 3: Figure 4f only shows a capacity retention rate of > 90% after 50 cycles, but does not provide structural characterization after cycling (such as SEM, XRD, Ag leaching). Suggest supplementing the morphology, crystal structure, and Ag^{+} leaching concentration of the electrode after cycling (6.2 μg L^{-1} is already available, and its comparative significance with environmental standards can be further discussed).

Response: We thank Reviewer 2# for this valuable comment. To better evaluate the structural robustness of the electrode, we have now extended the cycling test to 100 cycles and performed comprehensive post-cycling characterizations. The capacity retention of i-HMX@Ag_{20} electrode after 100 cycles can reach 78.6% (Supplementary Fig. 35), suggesting good cyclic stability. The post-cycling surface SEM image (Supplementary Fig. 36a) shows that the Ag NPs are still uniformly covered by the HMX nanosheets, with morphology similar to the initial state (Fig. 1h). The XRD analysis (Supplementary Fig. 36b) reveals that the characteristic peaks of MXene and metallic Ag remain clearly visible after cycling. Weak AgCl peaks are also detected, suggesting a small fraction of irreversible conversion after prolonged cycling. Nevertheless, the cross-sectional SEM image and EDS mapping (Supplementary Fig. 36c) confirm the intact layered architecture and homogeneous elemental distribution. Regarding Ag leaching, the Ag^{+} concentration after 50 cycles was 6.2 μg L^{-1}, and after 100 cycles it remained as low as 7.1 μg L^{-1}. Both values are well below the limit specified in the Chinese drinking water standard (50 μg L^{-1}, GB 5749-2022) and are well within the WHO guideline of 100 μg L^{-1}, demonstrating the excellent environmental safety and structural stability of the electrode. Following the reviewer's suggestion, these data have been added to the revised manuscript and Supplementary Information.

Page 23 line 8, "Even extending the cycling test to 100 cycles, the i-HMX@Ag_{20} electrode

can retain 78.6% of its initial capacity (Supplementary Fig. 35). Post-cycling characterization was carried out to examine its structural changes. As shown in Supplementary Fig. 36a, the Ag NPs remain uniformly covered by the HMX nanosheets, with a surface morphology similar to the fresh electrode (Fig. 1h). In addition to the peaks of MXene and metallic Ag observed in the XRD spectrum (Supplementary Fig. 36b), relatively weak AgCl diffraction peaks were also detected, indicating a minor degree of irreversible phase transformation after prolonged cycling. Nevertheless, its layered film structure remains intact, with homogeneous distribution of Ti, Ag, and C elements throughout the cross-section (Supplementary Fig. 36c). The Ag^+ leaching concentration detected in the solution after 50 cycles was only 6.2 μg L^{-1}, and after 100 cycles it remained as low as 7.1 μg L^{-1}. Both values are far below the safety limit of 50 μg L^{-1} stipulated by the standards for drinking water quality (GB 5749-2022, China) and are well within the WHO guideline of 100 μg L^{-1}. This extremely low Ag loss confirms that the covalent Ag–HMX interface not only anchors Ag species firmly but also prevents undesirable dissolution, thereby supporting both the structural stability and favorable environmental safety of the i-HMX@Ag_{20} electrode in long-term use."

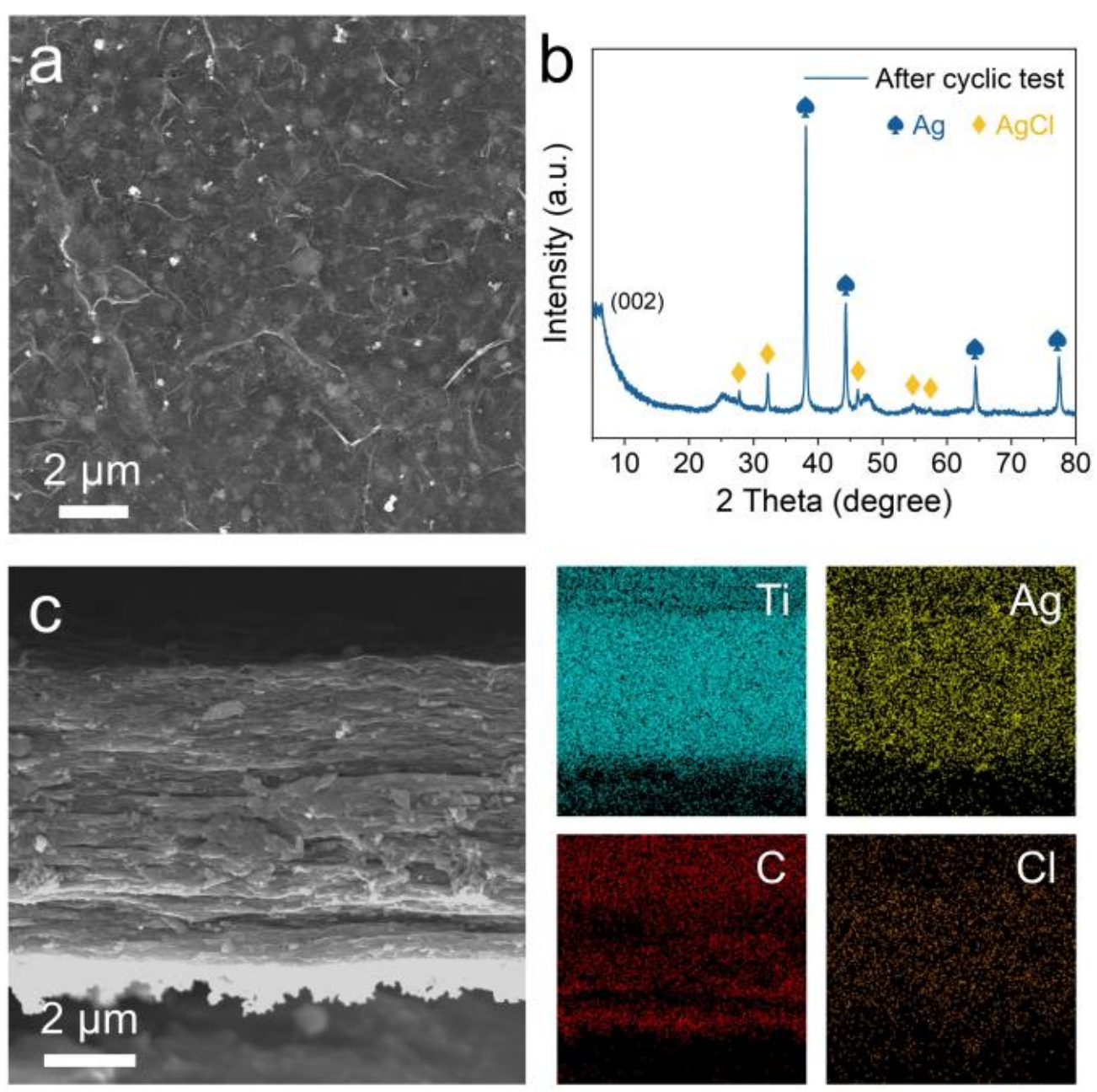

Supplementary Fig. 36. (a) Surface SEM image, **(b)** XRD pattern, and **(c)** cross-sectional SEM image and corresponding EDS elemental mapping of i-HMX@Ag_{20} film after cyclic test.

<u>Comment 4: When treating actual tap water and circulating cooling water in Figure 4h and i, it does not indicate whether pretreatment is carried out (such as filtration, pH adjustment, removal</u>

of organic matter, etc.), nor does it indicate the type and aging state of IEM. It is suggested to supplement this information to improve the reproducibility of the results.

Response: We appreciate Reviewer 2# for the careful reading and for the constructive comment regarding the practical applicability section. In the revised manuscript, we have now clarified these points as follows. First, the actual tap water was supplied by the municipal source, which was directly pumped into the multichannel series-connected CDI system for deionization without any pretreatment. Second, considering that the circulating cooling water in factories of different industries varies, we prepared a multicomponent solution to simulate its main water quality characteristics referring to previous study (*e.g., Angew. Chem. Int. Ed.,* ***2024****, 63, e202401972; Angew. Chem. Int. Ed.,* ***2025****, 64, e202504775*). Specifically, we used NaCl as the main source of Cl^-, Ca^{2+} and Mg^{2+} as the components of water hardness, and $NaHCO_3$ as the buffer to maintain the pH value of water at a slightly alkaline level. Overall, we did not undertake any additional preprocessing for the incoming water, which better demonstrates the practicality of our materials. Moreover, the cation exchange membrane (CMI-7000S) and the anion exchange membrane (AMI-7001S) were both purchased from Membrane International Inc. (USA), which were soaked in a 3~5 wt.% NaCl solution for 24 hours to fully hydrate and swell before use. The corresponding information have been supplemented in the revised manuscript.

Page 25 line 15, "The incoming water was directly pumped into the series-connected CDI system for processing without any additional pretreatment."

Page 34 line 3, "Specifically, the cation exchange membrane (CMI-7000S) and the anion exchange membrane (AMI-7001S) were purchased from Membrane International Inc. in USA. Before use, both of these IEMs needed to be immersed in a 3~5 wt.% NaCl solution for 24 h for hydration and swelling."

Page 34 line 13, "In the practical applicability experiments, the tap water was directly supplied by the municipal source. The circulating cooling water was simulated by dissolving 0.83 g of NaCl, 0.46 g of $CaCl_2$, 0.19 g of $MgCl_2 \cdot 6H_2O$, and 0.27 g $NaHCO_3$ in 1 L of DI water, where NaCl is the main source of Cl^-, Ca^{2+} and Mg^{2+} provide the hardness of water, and $NaHCO_3$ is buffer to maintain the pH value of water at a slightly alkaline level."

Comment 5: More experimental evidence is needed to verify the existence of interface electric fields, such as Kelvin probe force microscopy (KPFM).

Response: We thank Reviewer 2# for this valuable and insightful suggestion. We agree that Kelvin probe force microscopy (KPFM) can provide useful information on local surface potential variations and spatial charge separation behaviors at heterogeneous interfaces. In response to this valuable comment, we have now performed KPFM tests to probe the surface potentials of i-HMX@Ag_{20} and HMX/Ag using silicon wafers as conductive substrate. As shown in Supplementary Fig. 23, the surface potential differences between HMX and Ag nanoparticles in i-HMX@Ag_{20} and HMX/Ag are measured to be *ca.* 3.2 and 7.4 μV, respectively. The larger surface potential difference in HMX/Ag indicates the less electron transfer between HMX and Ag nanoparticles due to the severe interfacial resistance at the physical contact point. In contrast, the elaborate covalent link of i-HMX@Ag_{20} facilitates free electron flow between HMX and Ag and thus reduces the surface potential difference. These KPFM results are also consistent with the XPS results, jointly revealing the formation of interface electric field in i-HMX@Ag_{20}. The detailed experiments and descriptions have been added to the revised manuscript and Supplementary Information.

Page 14 line 12, "These results indicate that electrons are transferred between Ag NPs and the HMX support, likely through the covalently linked Ag−HMX interface, resulting in an interfacial built-in electric field and an active Ag surface."

Page 14 line 18, "To further explore the charge separation behaviors between Ag and HMX, Kelvin probe force microscopy (KPFM) was performed on i-HMX@Ag_{20} and HMX/Ag using silicon wafers as conductive substrate. As shown in Supplementary Fig. 23, the surface potential difference between HMX and Ag in i-HMX@Ag_{20} is *ca.* 3.2 μV, which is markedly smaller than that of HMX/Ag (*ca.* 7.4 μV). The larger potential difference in the physically mixed HMX/Ag sample indicates hindered electron transfer due to the large interfacial resistance at the point contacts. In contrast, the covalent Ag−HMX interface in i-HMX@Ag_{20} facilitates efficient electron flow, thereby reducing the potential difference, consistent with the XPS analyses."

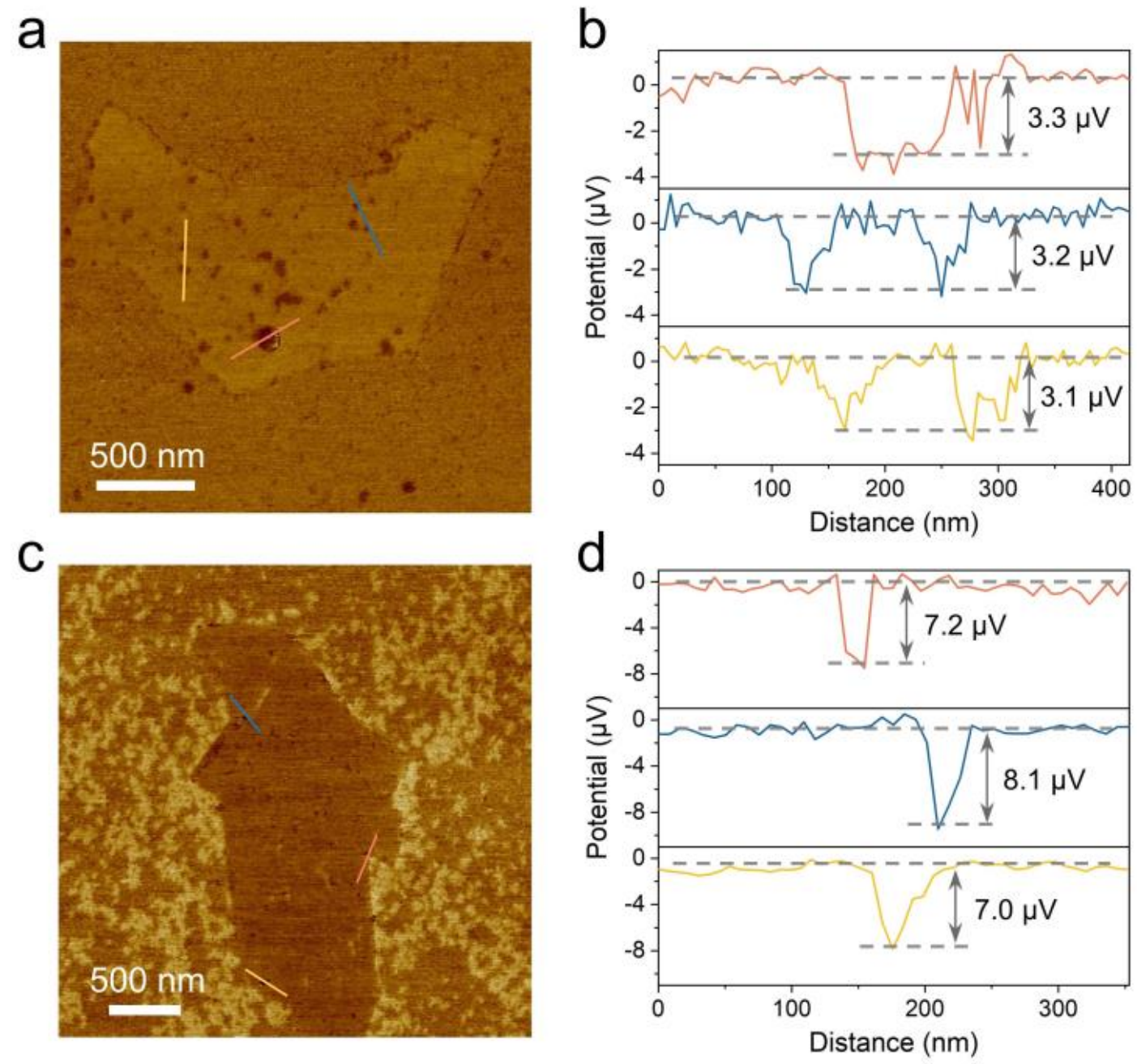

Supplementary Fig. 23. Kelvin probe force microscopy (KPFM) images and corresponding surface potentials of **(a, b)** i-HMX@Ag_{20} and **(c, d)** HMX/Ag.

Comment 6: Please provide the b-value and proportion of pseudocapacitance for the comparative sample.

Response: We thank Reviewer 2# for this helpful comment. Following this suggestion, we have calculated the b values and the capacitive contribution ratios for MXene and HMX/Ag using the same method as for i-HMX@Ag_{20}. As shown in Supplementary Fig. 31a, the b values of pristine MXene are around 0.95, indicative of predominantly surface-controlled capacitive behavior. The capacitive contribution at 5 mV s^{-1} reaches 84.0% (Supplementary Fig. 31b) and increases from 72.5% to 90.2% as the scan rate rises from 1 to 10 mV s^{-1} (Supplementary Fig. 31c), which is much higher than that of i-HMX@Ag_{20}. For HMX/Ag, the b values for the anodic and cathodic peaks are 0.66 and 0.82, respectively (Supplementary Fig. 31d). The capacitive contribution at 5 mV s^{-1} is 75.1% (Supplementary Fig. 31e), which is comparable to that of i-HMX@Ag_{20} (72.5% at the same scan rate). The capacitive contribution of HMX/Ag increases from 66.5% to 83.7% as the scan rate rises from 1 to 10 mV s^{-1} (Supplementary Fig. 31f). These results indicates that both HMX/Ag and i-HMX@Ag_{20} exhibit a mixed capacitive and diffusion charge-storage behavior. We have added the corresponding data and descriptions in the revised manuscript.

Page 19 line 17, "For comparison, we also evaluated the electrochemical kinetic behavior of

pristine MXene and HMX/Ag (Supplementary Fig. 31). Pristine MXene exhibits b values of *ca.* 0.95 and a high capacitive contribution of 84.0% at 5 mV s^{-1}, indicating predominantly surface-controlled charge-storage behavior. In contrast, HMX/Ag shows b values of 0.66 (anodic) and 0.82 (cathodic), with a capacitive contribution of 75.1% at 5 mV s^{-1}, which is comparable to that of i-HMX@Ag_{20}. These results indicate that both HMX/Ag and i-HMX@Ag_{20} display a mixed capacitive and diffusion-controlled behavior."

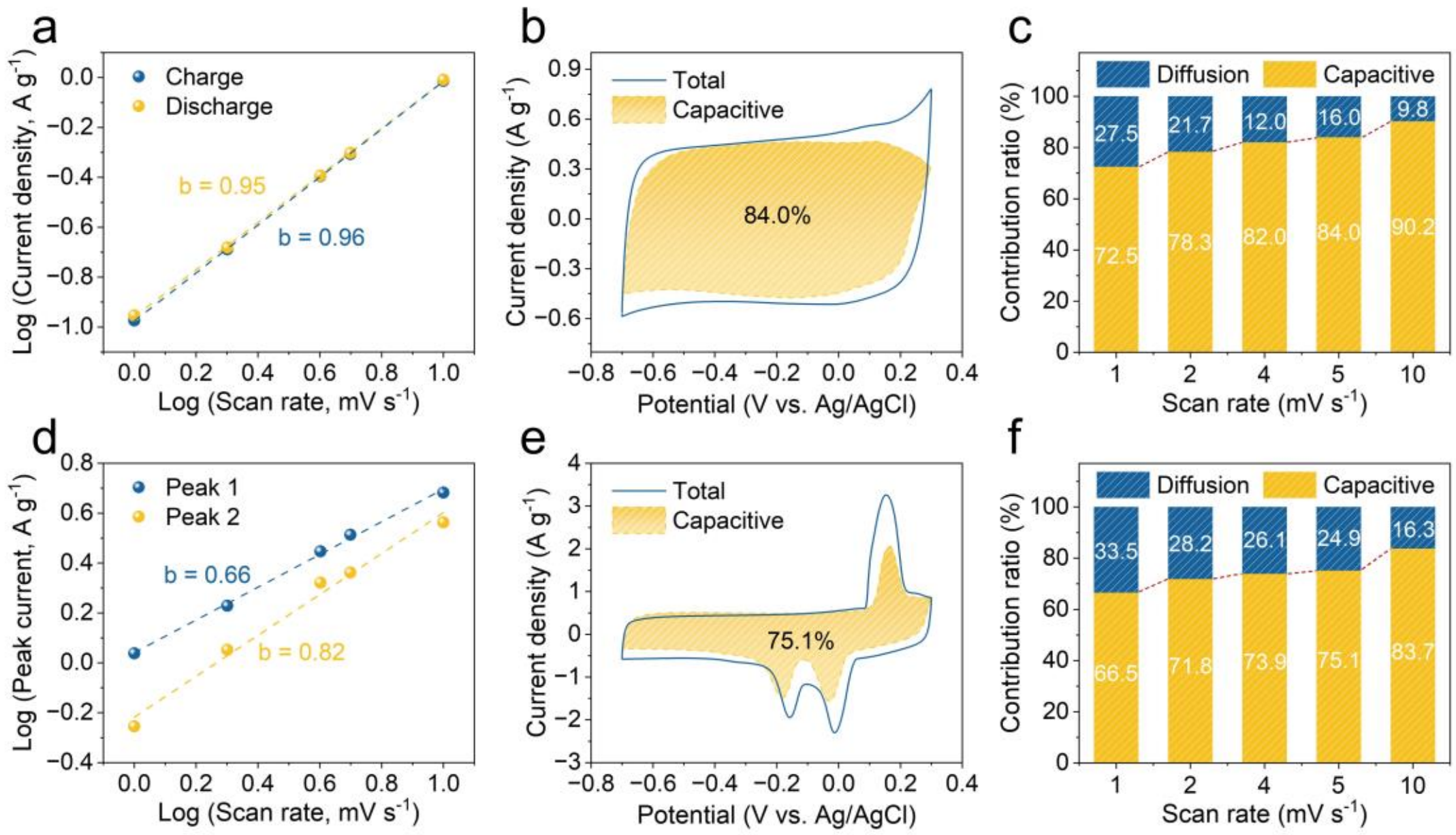

Supplementary Fig. 31. Determination of b-value, the capacitive contribution at 5 mV s^{-1}, and the contribution ratios of capacitive and diffusion at various scan rates of **(a-c)** MXene film and **(d-f)** HMX/Ag film.

Reviewer 3#:

General comments:

This work utilizes Ag single atoms as growth fulcrums to construct covalently linked Ag/MXene hybrid structures. The in situ etching–reduction strategy enables strong interfacial coupling between Ag species and MXene while simultaneously generating a porous MXene framework with enhanced ion transport pathways. The combination of Ag single atoms and nanoparticles further maximizes the utilization of redox active sites while mitigating volume changes. Furthermore, the authors provide a comprehensive understanding of the mechanism through a combination of electrochemical analysis and theoretical calculations, and validate its good performance in both

model and practical water systems. In summary, this work highlights a rational and multifunctional design strategy that combines interfacial chemistry with ion transport optimization for application in the field of capacitive deionization. The manuscript could be considered for publication after addressing the following comments.

Response: We sincerely thank Reviewer 3# for the pertinent and encouraging assessment of our manuscript. The reviewer's constructive comments have been very helpful in further improving the quality and clarity of the manuscript. In response to Reviewer 3#'s specific comments, we have revised the manuscript accordingly and provided a detailed point-by-point response below.

Comment 1: The authors repeatedly emphasize that the designed Ag/MXene architecture can effectively mitigate the severe volumetric expansion of Ag during the Ag/AgCl conversion process. However, there appears to be a lack of direct experimental evidence to substantiate this claim. For example, no in situ or ex situ characterization is provided to explicitly demonstrate the suppression of volume expansion. Therefore, it would be important to include more direct evidence to support this key advantage of the proposed design.

Response: We appreciate Reviewer 3# for raising this important point. To determine the suppression effect of MXene on the volume expansion of Ag, we have now compared the thickness change of i-HMX@Ag_{20} film electrode after adsorption and desorption using cross-sectional SEM analysis. The initial thickness of the freestanding i-HMX@Ag_{20} film is approximately 4.70 µm (Fig. 1g). After Cl^- adsorption (charging to 1.2 V in 500 mg L^{-1} NaCl solution), the film thickness increases to about 5.43 µm (Supplementary Fig. 37), corresponding to a volume expansion of approximately 15.5%. After desorption, the thickness nearly returns to its original value (4.83 µm), indicating its excellent structural reversibility. The significantly suppressed expansion observed in i-HMX@Ag_{20} electrode directly demonstrates that the porous and mechanically robust HMX network effectively buffers the volume change of Ag nanoparticles, preserving structural integrity and ensuring stable cycling performance. The corresponding data and statements have been added to the revised manuscript.

Page 23 line 23, "Moreover, the thickness of i-HMX@Ag_{20} film increased from 4.70 µm to 5.43 µm after Cl^- adsorption and recovered to 4.83 µm after desorption (Supplementary Fig. 37), demonstrating its excellent volumetric reversibility."

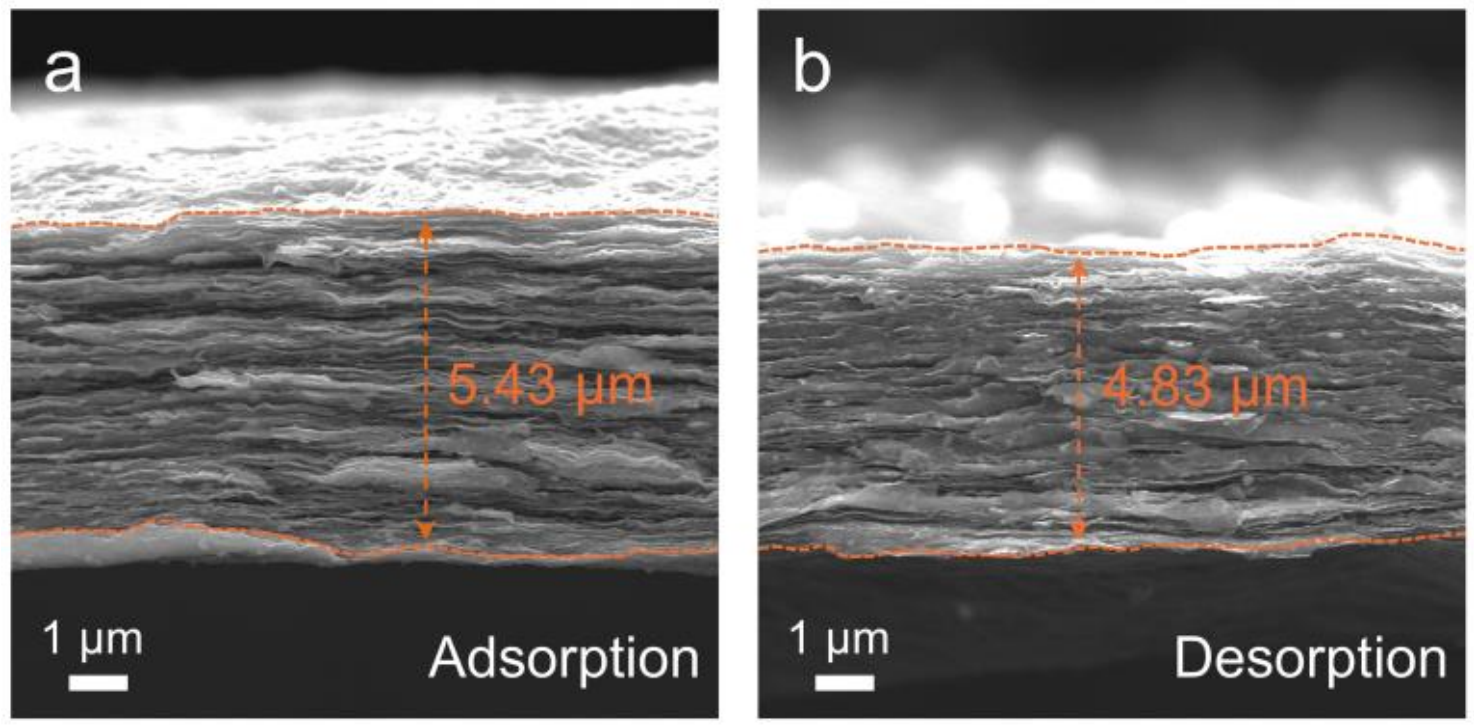

Supplementary Fig. 37. Cross-sectional SEM images of i-HMX@Ag_{20} film after **(a)** adsorption and **(b)** desorption.

Comment 2: How can the authors further quantitatively distinguish the actual capacity contribution from the Ag/AgCl conversion reaction? In particular, is it possible that a "pseudo-capacity amplification" effect, similar to that observed in conversion-type electrodes, leads to an overestimation of the faradaic contribution?

Response: We thank Reviewer 3# for the insightful comment. The reviewer raises the concept of "pseudo-capacity amplification", which is well documented in the lithium-ion battery literature for conversion-type electrodes (*e.g.*, Co_3O_4, Fe_2O_3). In those systems, the measured specific capacity often exceeds the theoretical value due to the formation of a gel-like polymer film from electrolyte decomposition at the electrode/electrolyte interface, which contributes additional charge storage (*Nat. Mater.*, ***2013***, *12, 1130-1136; Crystals,* ***2025***, *15, 832; Electrochim. Acta,* ***2012***, *61, 13-18*). However, this phenomenon is unlikely to be significant in the present Ag/AgCl-based CDI system. First, our CDI system uses an aqueous NaCl solution, not an organic electrolyte. The decomposition of water into a stable gel-like film on the electrode surface is negligible. Second, the reaction $Ag + Cl^{-} \rightleftharpoons AgCl + e^{-}$ is a well-defined, solid-to-solid transformation with a sharp interface between Ag and AgCl. We do not observe evidence for substantial parasitic interfacial products that could contribute extra capacity. Nevertheless, we have carefully quantified the faradaic contribution from the Ag/AgCl conversion using multiple independent methods based on our existing data.

(1) Kinetic analysis (b value and capacitive contribution). Utilizing the commonly used power-law relation ($i = av^b$), the b values for i-HMX@Ag_{20} were determined to be 0.62 for anodic peak and 0.75 for cathodic peak. These values lie between 0.5 (diffusion-limited battery behavior) and 1.0 (pure capacitive behavior) (Fig. 3g), indicating a mixed diffusion-controlled and surface-

controlled process. By contrast, the b values of pure MXene are 0.96 (charge) and 0.95 (discharge), suggesting its almost capacitive-controlled behavior (Supplementary Fig. 31). Notably, the CV peak of i-HMX@Ag_{20} is approximately equal to the rectangular-like shape of MXene plus the redox transformation peaks of Ag/AgCl (Fig. 3a). Moreover, the pure Ag/AgCl redox couple often exhibits sole sharp peaks without the rectangular-like part in aqueous NaCl solution (*Nat. Rev. Mater.*, ***2020***, *5*, *517-538*). Collectively, it can be reasonably inferred that the i-HMX@Ag_{20} electrode possesses a genuine faradaic Ag/AgCl conversion coupled with a moderate capacitive contribution from the MXene support without any overestimation.

(2) *Ex situ* XRD evidence. We performed *ex situ* XRD analysis on the i-HMX@Ag_{20} electrode at different stages of charging/discharging (Fig. 5a-c). During charging (Cl^- capture), the Ag diffraction peaks intensity progressively decreases, while the AgCl peaks intensity increases. During discharging (Cl^- release), the opposite trend is observed, and the XRD patterns almost completely recover to their initial state. Such reversible, stoichiometric phase transformation is direct evidence of a faradaic conversion reaction. If pseudo-capacity amplification were dominant, we would not observe such clear, reversible diffraction peak changes. The XRD data unequivocally support that the main charge storage mechanism is the faradaic reaction, not an artifact.

(3) Charge efficiency and Ag^+ leaching. The high charge efficiency (up to 90%) and the extremely low Ag^+ leaching (7.1 μg L^{-1} after 100 cycles) further rule out significant parasitic reactions that could lead to pseudo-capacity amplification. Any substantial electrolyte decomposition would likely lower the charge efficiency and increase Ag^+ release.

Based on the above multi-faceted evidence, we conclude that the observed Cl^- removal capacity is dominated by the faradaic Ag/AgCl conversion reaction, and there is no significant overestimation due to a "pseudo-capacity amplification" effect. The fraction of capacitive contribution comes mainly from the MXene/HMX support, which is intrinsic to the hybrid design and beneficial for rate capability. We thank the reviewer again for this valuable opportunity to clarify the mechanism. We have added a brief clarification of this point in the revised manuscript.

Page 19 line 10, "Given that the CV curve of i-HMX@Ag_{20} is approximately equal to the rectangular-like shape of MXene plus the redox transformation peaks of Ag/AgCl (Fig. 3a), this mixed electrochemical behavior originates from the genuine faradaic Ag/AgCl conversion coupled with the intrinsic capacitive contribution of the MXene support, rather than from any overestimation such as pseudo-capacity amplification."

Page 19 line 17, "For comparison, we also evaluated the electrochemical kinetic behavior of

pristine MXene and HMX/Ag (Supplementary Fig. 31). Pristine MXene exhibits b values of *ca.* 0.95 and a high capacitive contribution of 84.0% at 5 mV s^{-1}, indicating predominantly surface-controlled charge-storage behavior."

Page 26 line 20, "Concurrently, the progressive attenuation of the Ag diffraction peaks alongside the growing intensity of the AgCl peaks (Fig. 5b, c) marks the substantial phase transition from Ag to AgCl following Cl^- capture in the i-HMX@Ag_{20} electrode, suggesting the dominated faradaic nature of the charge storage."

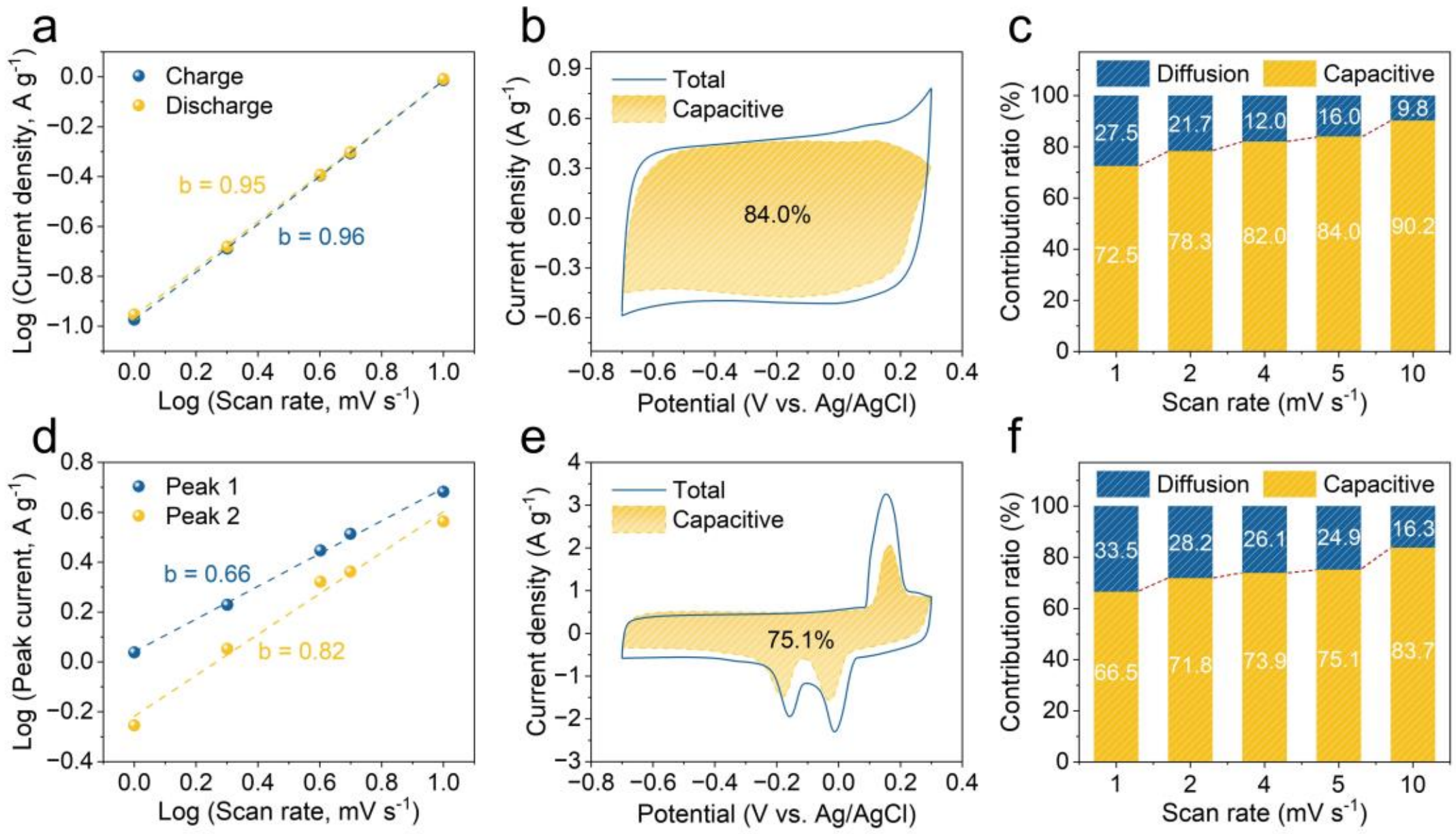

Supplementary Fig. 31. Determination of b-value, the capacitive contribution at 5 mV s^{-1}, and the contribution ratios of capacitive and diffusion at various scan rates of **(a-c)** MXene film and **(d-f)** HMX/Ag film.

Comment 3: The authors attribute the significantly reduced R_{ct} to the synergistic effect of the Ag–HMX interface. However, EIS parameters can also be strongly affected by structural factors, such as pore structure, film thickness, and electrode architecture. Therefore, it is not clear whether the reduced R_{ct} mainly originates from interfacial electronic coupling or from structural optimization. Additional evidence or control experiments would be helpful to distinguish between these contributions.

Response: We appreciate Reviewer 3# for this important comment. We agree that EIS parameters can be influenced by structural factors such as pore architecture, film thickness, and interlayer spacing. The reduced charge transfer resistance (R_{ct}) of i-HMX@Ag_{20} cannot be attributed solely

to the covalent Ag−HMX interface, as structural factors also play a role. First, compared with pristine MXene electrode (non-porous, small interlayer spacing of 12.33 Å, and no Ag decoration), i-HMX@Ag_{20} possesses a well-developed in-plane porous network, an expanded interlayer spacing (12.72 Å), and a highly conductive Ag-bridged architecture. These structural features can facilitate ion/electron transport and lower R_{ct}. To isolate the contribution of the covalent Ag−HMX interface, we deliberately designed the HMX/Ag control sample to have comparable in-plane porosity (*via* H_2O_2 etching) and similar film thickness (*via* identical vacuum filtration conditions). The main structural differences between the two samples are: (i) a larger interlayer spacing in i-HMX@Ag_{20} (12.72 Å *vs.* 12.37 Å for HMX/Ag, as shown in Fig. 1f and Supplementary Fig. 9b), and (ii) the presence of a covalent Ag−HMX interface in i-HMX@Ag_{20}, which is absent in the physically mixed HMX/Ag. Both factors can contribute to the reduced charge transfer resistance. We acknowledge that achieving two samples with identical pore structure and interlayer spacing while differing only in interfacial chemistry is experimentally challenging. The current HMX/Ag is already a very close and comparable control sample that has been optimized by us. Moreover, the interlayer spacing difference between i-HMX@Ag_{20} and HMX/Ag is only 0.35 Å. Considering the hydrated radius of Cl^- is about 3.6 Å, this small spacing variation is unlikely to dominate the ion transport kinetics. Therefore, the significantly lower R_{ct} of i-HMX@Ag_{20} (0.75 Ω *vs.* 2.21 Ω for HMX/Ag) can be reasonably attributed primarily to the covalent Ag−HMX interface, which greatly accelerates electron transfer, while the expanded spacing provides a secondary but beneficial contribution. To maintain scientific rigor, we have added a clarifying statement in the revised manuscript.

Page 8 line 6, "The HMX framework was produced *ex situ via* a H_2O_2-assisted etching treatment[33]. It should be noted that both Ag^+ and H_2O_2 create in-plane holes in MXene through selectively oxidative etching of Ti species, accompanied by the removal of surface terminal groups, but their reaction kinetics differ. By fine-tuning the etching conditions of H_2O_2, the produced HMX exhibits in-plane porosity and thickness comparable to that of i-HMX@Ag_{20} (Supplementary Fig. 7)."

Page 18 line 21, "According to the fitted equivalent electric circuit (inset of Fig. 3e), the combined series resistance (R_s) and the charge transfer resistance (R_{ct}) of i-HMX@Ag_{20} (R_s = 2.39 Ω, R_{ct} = 0.75 Ω) are markedly lower than those of HMX/Ag (R_s = 5.03 Ω, R_{ct} = 2.21 Ω) and MXene (R_s = 3.34 Ω, R_{ct} = 11.39 Ω) (Supplementary Table 3), demonstrating its superior electron transport behavior arising from the synergistic combination of an expanded interlayer spacing, a

well-developed porous network, and the unique interfacial coordination mode[51]."

Comment 4: The enhanced dechlorination performance is attributed to "electron-deficient Ag facilitating faradaic dechlorination." However, based on the CDI results, the system still exhibits typical capacitive behavior. How do the authors distinguish and quantitatively determine the respective contributions of faradaic reactions and electric double-layer adsorption in Cl^- removal? Are there in situ or ex situ analyses to support this mechanism?

Response: We thank Reviewer 3# for the valuable and insightful comment. We acknowledge that strictly separating the faradaic contribution from electric double-layer (EDL) adsorption in a hybrid system is challenging, as both processes occur simultaneously and interact with each other. Nevertheless, multiple independent lines of evidence from our data allow us to qualitatively and semi-quantitatively distinguish the dominant faradaic role. **(1) Dechlorination capacity comparison with reference electrodes**. Briefly, pristine MXene electrode provides a baseline EDL or pseudocapacitive capacity of 40.87 ± 2.7 mg g^{-1} (Fig. 4a). The much higher capacity of i-HMX@Ag_{20} (133.34 ± 4.4 mg g^{-1}) implies that the excess capacity (~92.5 mg g^{-1}) originates from the faradaic Ag/AgCl conversion. This simple subtraction provides a lower-bound estimate of the faradaic contribution. **(2) *Ex situ* XRD evidence of reversible phase transformation**. As shown in Fig. 5a-c, during Cl^- capture the Ag diffraction peaks decrease while AgCl peaks appear; during Cl^- release the opposite occurs. Such a reversible, stoichiometric phase transition is unambiguous evidence that a substantial portion of Cl^- is removed *via* faradaic reaction, not by EDL adsorption. Without this conversion, the capacity would be purely capacitive. **(3) Charge efficiency**. The charge efficiency of i-HMX@Ag_{20} reaches ~90% (Fig. 4c). In an ideal EDL process, charge efficiency is typically lower (60-80%) due to co-ion repulsion, while a faradaic process often gives a higher efficiency because each electron corresponds to one Cl^- captured. Our high efficiency is fully consistent with a faradaic-dominated mechanism. **(4) Kinetic b values and CV shape**. The b values (0.62 for anodic, 0.75 for cathodic) lie between 0.5 (diffusion-controlled battery) and 1.0 (capacitive). The CV curves (Fig. 3a) show distinct Ag/AgCl redox peaks superimposed on a pseudocapacitive background. This mixed behavior indicates that while a capacitive component exists, the faradaic contribution is substantial. **(5) *Ex situ* XPS of Ag species**. The reversible change in the Ag^+/Ag^0 ratio (Fig. 5d) directly confirms the participation of Ag in the faradaic reaction. Overall, although perfect quantification is difficult, the convergence of the above multiple complementary experiments clearly demonstrates that the majority of Cl^- removal is achieved *via*

the faradaic Ag/AgCl conversion, with EDL adsorption playing a minor, auxiliary role. We have added a concise discussion in the revised manuscript.

Page 19 line 10, "Given that the CV curve of i-HMX@Ag_{20} is approximately equal to the rectangular-like shape of MXene plus the redox transformation peaks of Ag/AgCl (Fig. 3a), this mixed electrochemical behavior originates from the genuine faradaic Ag/AgCl conversion coupled with the intrinsic capacitive contribution of the MXene support, rather than from any overestimation such as pseudo-capacity amplification."

Page 26 line 20, "Concurrently, the progressive attenuation of the Ag diffraction peaks alongside the growing intensity of the AgCl peaks (Fig. 5b, c) marks the substantial phase transition from Ag to AgCl following Cl^- capture in the i-HMX@Ag_{20} electrode, suggesting the dominated faradaic nature of the charge storage."

Page 27 line 14, "It should be noted that while completely decoupling the faradaic and capacitive contributions is difficult, the much higher capacity of i-HMX@Ag_{20} than pristine MXene, the reversible Ag/AgCl phase transition observed by *ex situ* XRD (Fig. 5b, c), and the high charge efficiency (~90%) collectively confirm that the faradaic reaction is the dominant Cl^- removal pathway."

Comment 5: The cycling stability was evaluated over only 50 cycles, which appears relatively limited. Could the authors provide longer-term cycling data to better assess the durability and practical reliability of the electrode?

Response: We thank Reviewer 3# for the constructive comment. In response to this pertinent suggestion, we have extended the cycling test to 100 cycles to better evaluate the long-term durability of the i-HMX@Ag_{20} electrode. As shown in Supplementary Fig. 35, the electrode retains 78.6% of its initial dechlorination capacity after 100 cycles, confirming satisfactory long-term stability. Moreover, we conducted post-cycling characterization analyses to further trace its structural changes (Supplementary Fig. 36). The surface SEM image reveals that the HMX nanosheets still effectively cover and confine the Ag NPs. XRD shows that the Ag and MXene phases are well preserved, with minor AgCl peaks likely accounting for the gradual capacity loss. The cross-sectional SEM image indicates that the layered film architecture remains intact, with Ti, Ag, and C elements homogeneously distributing throughout the cross-section. In addition, the leached Ag^+ in the post-cycling solution was as low as 7.1 μg L^{-1} after 100 cycles, far below the

Chinese drinking water standard (50 μg L^{-1}, GB 5749-2022) and the WHO guideline (100 μg L^{-1}). These results indicate that the i-HMX@Ag_{20} electrode can sustain extended operation with good structural stability and environmental safety. We have added these data and descriptions to the revised manuscript and Supplementary Information.

Page 23 line 8, "Even extending the cycling test to 100 cycles, the i-HMX@Ag_{20} electrode can retain 78.6% of its initial capacity (Supplementary Fig. 35). Post-cycling characterization was carried out to examine its structural changes. As shown in Supplementary Fig. 36a, the Ag NPs remain uniformly covered by the HMX nanosheets, with a surface morphology similar to the fresh electrode (Fig. 1h). In addition to the peaks of MXene and metallic Ag observed in the XRD spectrum (Supplementary Fig. 36b), relatively weak AgCl diffraction peaks were also detected, indicating a minor degree of irreversible phase transformation after prolonged cycling. Nevertheless, its layered film structure remains intact, with homogeneous distribution of Ti, Ag, and C elements throughout the cross-section (Supplementary Fig. 36c). The Ag^+ leaching concentration detected in the solution after 50 cycles was only 6.2 μg L^{-1}, and after 100 cycles it remained as low as 7.1 μg L^{-1}. Both values are far below the safety limit of 50 μg L^{-1} stipulated by the standards for drinking water quality (GB 5749-2022, China) and are well within the WHO guideline of 100 μg L^{-1}. This extremely low Ag loss confirms that the covalent Ag−HMX interface not only anchors Ag species firmly but also prevents undesirable dissolution, thereby supporting both the structural stability and favorable environmental safety of the i-HMX@Ag_{20} electrode in long-term use."

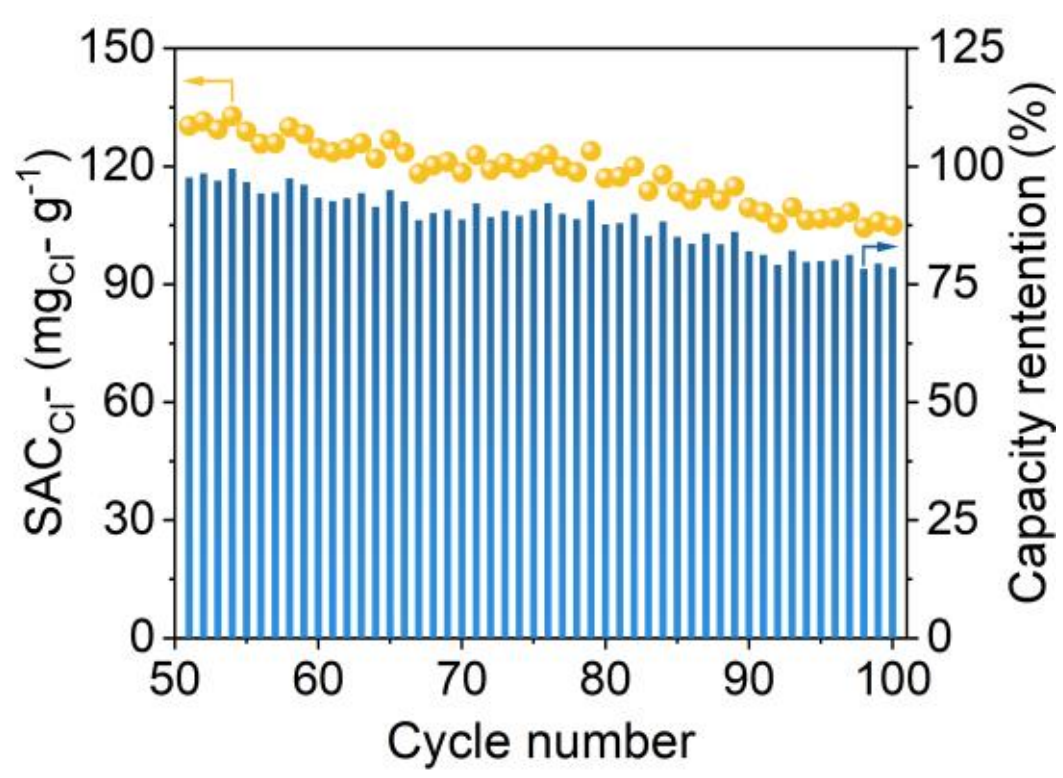

Supplementary Fig. 35. Cyclic stability of i-HMX@Ag_{20} electrode over 51 to 100 cycles.

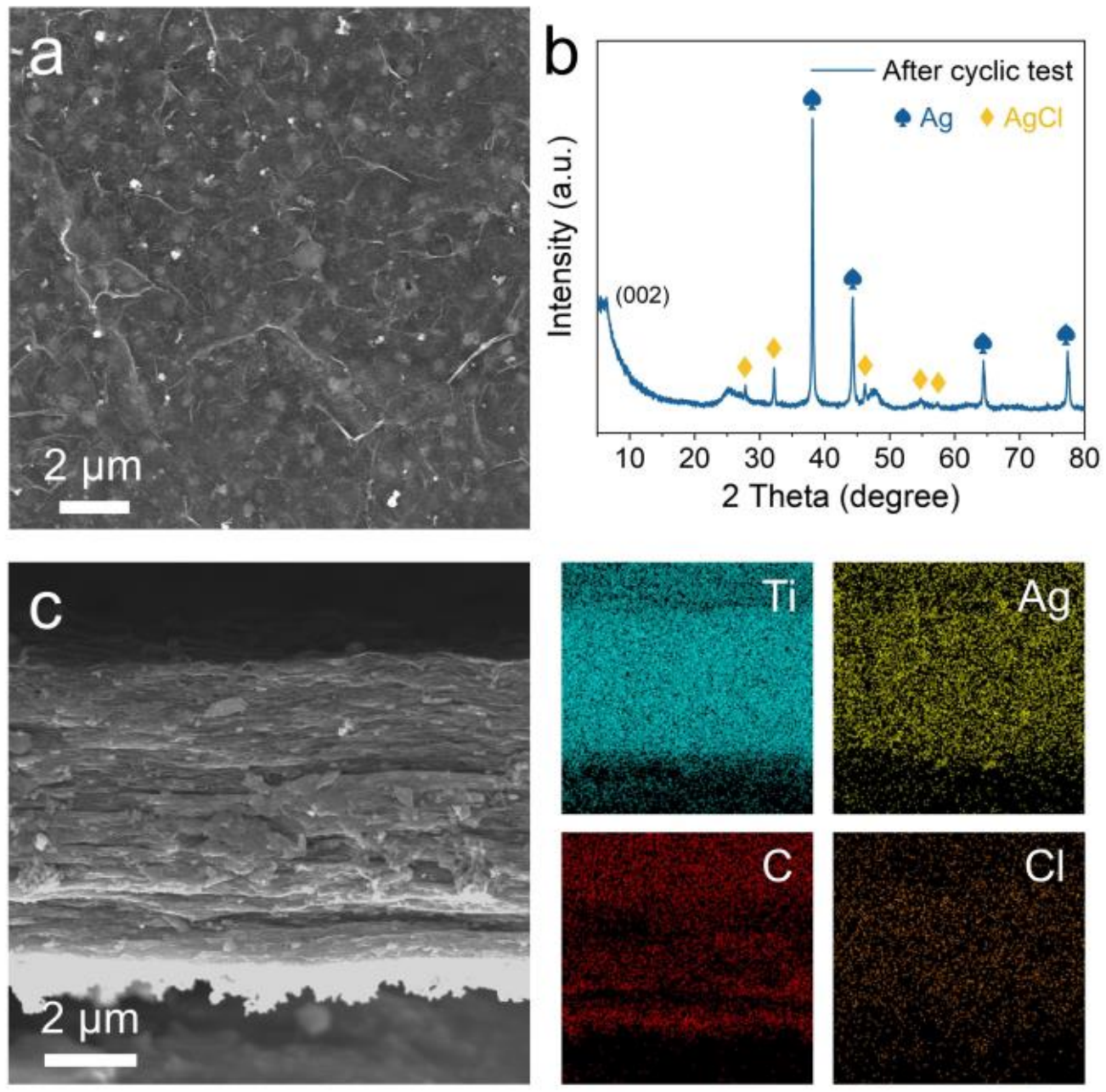

Supplementary Fig. 36. (a) Surface SEM image, **(b)** XRD pattern, and **(c)** cross-sectional SEM image and corresponding EDS elemental mapping of i-HMX@Ag_{20} film after cyclic test.

Comment 6: Considering the relatively high cost of Ag, could the authors provide an evaluation of the economic feasibility of this material? In addition, how does the performance and cost-effectiveness compare with conventional carbon-based CDI electrodes?

Response: We thank Reviewer 3# for raising the important issue of economic feasibility. Based on the ICP-OES result, the actual Ag content in the i-HMX@Ag_{20} electrode is 8.48 wt.% (Supplementary Table 1). Using the recent market price of Ag (18765 CNY kg^{-1} as of May 19, 2026, Shanghai Metals Market), the raw material cost of Ag per gram of electrode is approximately 1.59 CNY g^{-1}. For comparison, the bulk price of industrial-grade activated carbon is approximately 2500 CNY ton^{-1} for 500-iodine grade (approximately 0.0025 CNY g^{-1}), while higher-quality activated carbon (*e.g.*, with iodine value of 800-1150 mg g^{-1}) can range from 9500 to 13000 CNY ton^{-1}. We acknowledge that the use of Ag inevitably increases the material cost compared with carbon-based electrodes. However, three points are worth noting. **First**, the i-HMX@Ag_{20} electrode achieves a high dechlorination capacity of 133.34 ± 4.4 mg g^{-1} at 1.2 V in 500 mg L^{-1} NaCl solution (Fig. 4a), which is 5-6 times higher than that of typical activated carbon (~25 mg g^{-1}). Moreover, the electrode retains 78.6% of its initial capacity after 100 cycles (Supplementary Fig. 35) and exhibits an extremely low Ag^+ leaching of only 7.1 μg L^{-1} after 100 cycles. This superior durability significantly extends the service life, thereby reducing the long-term

replacement frequency and total operational cost. **Second**, Ag/AgCl is a well-established chloride-capture system in the CDI field, widely used for its high theoretical capacity (~250 mAh g^{-1}), excellent Cl^- selectivity, and low redox potential. The use of Ag in faradaic dechlorination electrodes is therefore a mature and recognized research direction, not an arbitrary choice. **Third**, and most importantly, the primary contribution of this work is the design strategy (the covalent Ag−HMX interface and the *in situ* pore engineering) rather than the specific use of Ag itself. This design principle can be extended to other metals that are reducible by the low-valence Ti species on MXene, including more abundant and lower-cost alternatives. The covalent interfacial bridging concept offers a general platform for constructing high-performance hybrid electrodes. With this design guidance, future efforts can focus on replacing Ag with more cost-effective active components while retaining the interfacial engineering benefits, thereby achieving a balance between performance and economic viability. We thank the reviewer again for this constructive comment, which has helped us better articulate the broader impact of our work. We have added a brief discussion of this economic perspective and the design generality in the revised manuscript.

Page 30 line 14, "Economically, although the use of Ag inevitably raises the material cost, our interfacial design strategy is inherently extensible to other hybrid systems, thereby providing a general design principle for developing high-performance yet cost-effective hybrid electrodes."
